# Evaluation and optimization of SPE pre-processing combined with V-ITEX-GC-MS versus direct V-ITEX-GC-MS for urinary volatilome profiling

**DOI:** 10.1007/s00216-026-06469-3

**Published:** 2026-03-31

**Authors:** Lucie K. Tintrop, Doris Zbinden, Marie-Claire Risse, Pascal Fuchsmann

**Affiliations:** Agroscope, Schwarzenburgstrasse 161, 3003 Bern, Switzerland

**Keywords:** Metabolomics, Urine, Vacuum in-tube extraction, Microextraction, Solid-phase extraction

## Abstract

Volatile urinary metabolites offer valuable insights into nutritional status and metabolic function. However, the extraction of these metabolites remains analytically challenging due to the complexity and variability of the urine matrix. This study compares and optimizes two extraction techniques, solid-phase extraction followed by vacuum in-tube extraction (SPE+V-ITEX), and direct V-ITEX for the untargeted gas chromatography-mass spectrometry analysis of urinary volatiles. Using design of experiment (DoE) approaches, critical parameters were systematically optimized for both methods. The identified compounds were analyzed with respect to origin, extraction efficiency, and chemical class specificity. Method greenness was assessed using the AGREE and AGREEprep tools. Both SPE+V-ITEX and direct V-ITEX enabled broad metabolite coverage, yielding 1198 detected features, with sufficient repeatability, as 76% of evaluated responses showed RSD values below 20%. SPE+V-ITEX achieved higher total signal intensity for several compounds and compound groups (e.g., alcohols, caprolactam, hetero aromats), and both methods demonstrated sufficient repeatability. Temporal trends in pooled real samples confirmed both methods are applicable for metabolite profiling. Based on AGREE and AGREEprep assessments, V-ITEX emerged as a more sustainable and operationally efficient workflow, aligning closely with green analytical chemistry principles. Desorption flow, trap material, extraction temperature, and time significantly influenced direct V-ITEX performance. Compound-specific trends were observed, but no single method was optimal for all metabolite classes. These findings present direct V-ITEX as a robust, green, and efficient alternative to SPE+V-ITEX for urinary volatilomics and a method particularly well-suited for high-throughput analytical workflows with minimal manual steps.

**Graphical abstract:**

**Figure:**
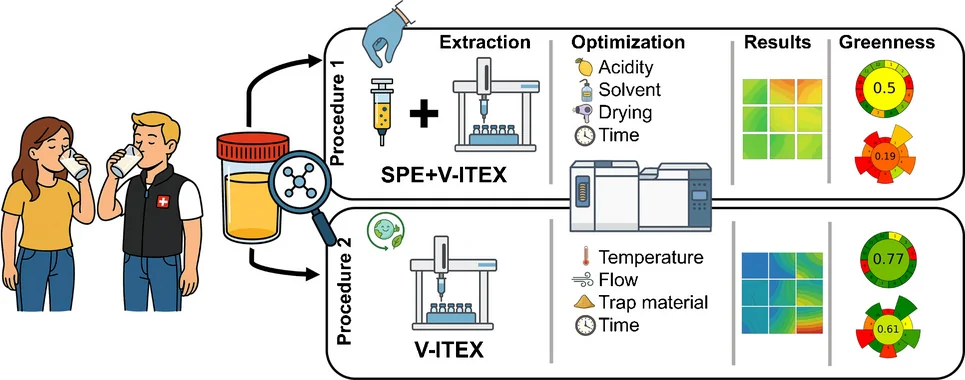

**Supplementary Information:**

The online version contains supplementary material available at https://doi.org/10.1007/s00216-026-06469-3.

## Introduction

Human nutrition is a topic of growing importance in both research and public health. Diet profoundly affects metabolic health and overall well-being. However, the metabolic pathways through which food is processed in the human body are not yet fully understood. A multitude of individual factors, including sex [1, 2], age [3], and health conditions [4], significantly influence the metabolic response to food intake. In the context of rising interest in personalized nutrition, understanding how individuals metabolize specific dietary components is essential for optimizing dietary recommendations, developing functional foods, and advancing precision nutrition [5]. To capture these complex and personalized nutritional dynamics, metabolomics has emerged as a key analytical approach. It enables the characterization of physiological states and the identification of diet-induced metabolic shifts [4, 6]. Nutritional metabolomics investigates how foods, nutrients, and dietary patterns influence the human metabolome, offering powerful insights into food-host interactions.

Among the biofluids used in metabolomic studies, urine is particularly favored. It can be collected non-invasively, at home, and in large quantities. It is nearly free of proteins and lipids but is characterized by a high chemical diversity, including a substantial fraction of conjugated metabolites that may complicate downstream analysis. Although the urinary metabolome represents a subset of the serum metabolome, kidney function concentrates metabolites to such an extent that compounds often remain below the limit of detection (LOD) in serum but are very well detectable in urine [6, 7]. Urinary metabolites originate from different sources within the body, including blood plasma filtration, cell sloughing, apoptosis, proteolytic cleavage of cell surface proteins, the secretion of exosomes by epithelial cells, and microbial metabolism [6–8].

A comparative study of analytical platforms has shown that proton nuclear magnetic resonance (^1^H NMR) spectroscopy, high-performance liquid chromatography-mass spectrometry (HPLC-MS), and gas chromatography-mass spectrometry (GC-MS) each provide complementary coverage of urinary metabolites [6]. HPLC-MS enables detection of compounds not accessible by GC-MS or NMR, such as isoflavones, and is generally more sensitive, whereas GC-MS is particularly effective for the analysis of volatile and semi-volatile compounds. GC-MS of derivatized non-volatiles yields results comparable to NMR, while GC-MS analysis of volatiles has been shown to offer additional information with minimal overlap in identified compounds [6]. As such, volatile urine metabolite profiling by GC-MS offers unique insights and remains a promising yet underexplored approach in nutritional metabolomics.

A variety of extraction methods are employed to isolate metabolites from complex biological matrices. Solid-phase extraction (SPE) is widely utilized due to its efficacy. However, concerns about solvent use and the avoidance of manual steps in the sample preparation procedure have led to increased interest in solvent-free microextraction methods [9]. In recent years, techniques such as solid-phase microextraction (SPME) [10], in-tube extraction (ITEX) [11], and stir-bar sorptive extraction (SBSE) [12] have gained popularity. Vacuum in-tube extraction (V-ITEX), introduced by Fuchsmann et al. [13], builds upon classical ITEX by incorporating vacuum assistance, which accelerates the gas-phase transition of analytes. This enhances extraction efficiency and expands the range to aqueous samples by improving the volatilization of polar compounds [13].

In previous studies, SPE+V-ITEX was successfully applied for the extraction of urinary volatiles, offering high analytical sensitivity but requiring multiple preparation steps and organic solvents [3, 14, 15]. While direct V-ITEX represents a greener, fully automated alternative, its applicability to urine has been questioned in the literature due to concerns regarding insufficient sensitivity compared to SPE+V-ITEX [14]. In this study, we systematically optimize the parameters of both methods and compare their performances. This allows evaluation of their potential as a simplified, solvent-free, and sustainable alternative.

Despite these technological advances, there is a continued need for comprehensive comparative studies of extraction techniques, particularly in the context of volatile urinary metabolite analysis related to food intake. This study addresses this gap by optimizing both methods, evaluating compound-class-specific performance, and comparing their analytical and environmental characteristics.

## Materials and methods

### Samples and solutions

Hexane, acetone, ethyl acetate, dichloromethane, acetonitrile, alkane mix, and 85% H_3_PO_4_ were purchased from Merck (Buchs, Switzerland). Milli-Q Water was withdrawn from a Milli-Q System (IQ 7000, 18.2 MΩ, 1.8 ppb TOC, Merck, Buchs, Switzerland).

The urine samples were collected from four healthy, voluntary male individuals during screening experiments for the FermentoMilk project after consumption of 250 mL of whole milk. The FermentoMilk project investigates the effect of dairy and fermented food diet on the urinary metabolome of young and older men. The individuals for these screening experiments had a body mass index of 19–27 and were born between 1962 and 1982 (42–63 years old). Urine samples were taken before milk intake (t_0_), during 3 h after milk intake (t_3_), and during 3–6 h after milk intake (t_6_). If multiple urine samples from the same person were available during the same time period, they were combined. For the extraction optimization presented in this study, a pool sample was prepared from all collected samples and stored at −40 °C.

### GC-MS analysis

For GC-MS analysis, a gas chromatograph (GC) 7890 B coupled to a mass spectrometer (MS) 5977 B with a high-efficiency source (HES) was used, both of which were purchased from Agilent (Basel, Switzerland). Analyte separation was performed through an Optima 5-MS Accent capillary column (30 m × 250 µm × 0.25 µm, Machery-Nagel, Reinach, Switzerland) with a 5% diphenyl-95% dimethylpolysiloxane, nonpolar-low polar GC phase, operated with a column flow of 0.8 mL/min (velocity of 32.424 cm/s) and helium as the carrier gas (Carbagas, Gümligen, Switzerland). The oven temperature program was as follows: held for 5 min at 40 °C, raised at a rate of 10 °C/min to 280 °C, and held for 5 min. Thermal desorption and injection were performed for 4 min using a cooled injection system (CIS) fitted with a Tenax TA-packed liner (both purchased by Gerstel, Sursee, Switzerland) and operated with a homemade cooling system based on compressed air (6 bar) and a Julabo cryostat, model CP 800 F (Huberlabs, Aesch, Switzerland). The cryostat was operated at a temperature of −20 °C. The CIS operated in solvent vent mode with a purge flow to split vent of 130 mL/min and a temperature of 10 °C for desorption and ramping with a rate of 12 °C/s to 300 °C after complete desorption. The ion source, quadrupole, and transfer line were set to 230 °C, 150 °C, and 280 °C, respectively. The MS scanned a mass range of 35–400 m/z. Compound identification was performed using MassHunter Workstation Unknowns Analysis (version 11.1, Agilent, Basel, Switzerland) with a match factor threshold of > 80%, based on spectral comparison of the NIST database (NIST23, National Institute of Standards and Technology, Gaithersburg, USA) and the comparison of the NIST database retention indices (RIs) with experimentally determined RIs (allowed deviation ± 15). Identification levels [1–4] were defined according to the requirements of the Chemical Analysis Working Group of the Metabolomics Standards Initiative [16, 17].

### V-ITEX GC-MS: general workflow

V-ITEX was first described by Fuchsmann et al. [13]. A multipurpose sampler (MPS, Gerstel, Sursee, Switzerland) was equipped with an ITEX tool and syringe, connected with a modified valve to a vacuum pump (V-300, Büchi, Flawil, Switzerland) operated at 5 mbar [13]. The general workflow of the autosampler was as follows: the sample was retrieved from the cooled rack (4 °C) and placed into the heated agitator for a 10-min incubation. The metabolites were then extracted from the vials’ headspace and thermally desorbed with a trap temperature of 300 °C. After desorption, the ITEX trap was thermally cleaned at 300 °C for 15 min. Other extraction parameters were varied during method optimization and are described in the following section. V-ITEX was applied for the direct analysis of both the urine samples and the SPE eluates.

### Optimized V-ITEX procedure

Table 1 provides an overview of the optimized parameters for different compound classes and should be used as a guide for adapting the extraction conditions to specific analytical targets. Figure S1 in the Electronic Supplementary Material (ESM) shows the workflow of the procedure. Based on the DoE optimization, the final direct V-ITEX procedure was defined as follows: 200 µL of urine were transferred into a 20-mL headspace vial and incubated for 10 min at the extraction temperature. Extraction was performed under vacuum using a Tenax TA trap for 10 min at 90 °C, followed by thermal desorption at 300 °C with a desorption flow of 400 mL/min. The extracted analytes were cryofocused in the CIS and analyzed by GC-MS under the conditions described above. A water removal strategy was performed for the V-ITEX system to avoid entering water from the urine samples in the GC-MS system. After extraction, the ITEX trap was purged for 2 min with N_2_ to remove the remaining water from the trap material at 35 °C. The tubes were purged for 5 min with N_2_.

**Table 1:** Optimal values for the different groups of the optimization of SPE and ITEX procedure determined by DoE

	SPE procedure				V-ITEX procedure			
	Drying proc	Extr. time (min)	Solvent	Acid conc. (v/v%)	Extr. time (min)	Extr. temp. (°C)	Des. flow (mL/min)	Trap material
**All**	Air/Vac	10	Dichloromethane	6	10	90	400	Tenax TA
**Alcohols**	N_2_	0.5	Acetone	6	10	90	400	Tenax TA
**Aldehydes**	Air/Vac	10	Dichloromethane	6	10	90	400	Tenax TA
**Aliphatic hydrocarbons**	Air/Vac	10	Dichloromethane	0	0.5	40	400	Tenax TA/Carboxen
**Aromatic hydrocarbons**	Air/Vac	10	Dichloromethane	5.4	10	90	400	Tenax TA
**Azines (1,3,5-triazine-2,4,6(1H,3H,5H)-trione, 1,3,5-tri-2-propen-1-yl-)**	Air/Vac	10	Dichloromethane	0	0.5	90	400	Tenax TA
**Cyclic hydrocarbons**	Air/Vac	10	Dichloromethane	6	10	90	20	Carbosieve SIII
**Esters**	Air/Vac.	10	Ethyl acetate	0	10	90	400	Tenax TA
**Hetero aromats**	N_2_	0.5	Acetonitrile	6	0.5	40	40	Carboxen
**Ketones**	N_2_	0.5	Acetone	0	10	90	400	Tenax TA
**Lactams (caprolactam)**	Air/Vac.	0.5	Ethyl acetate	0.04	0.5	90	400	Tenax TA
**Lactones**	Air/Vac	10	Dichloromethane	6	10	90	400	Tenax TA
**Nitriles**	Air/Vac	10	Acetone	0	10	90	400	Tenax TA
**Phthalates**	Air/Vac	0.5	Dichloromethane	0	0.5	40	20	Carboxen
**Substituted aromats**	N_2_	0.5	Acetonitrile	6	10	90	400	Tenax TA
**Sulfones (dimethyl sulfone)**	Air/Vac.	0.5	Dichloromethane	6	0.5	40	20	Carboxen
**Terpenes**	N_2_	0.5	Acetone	6	10	90	400	Tenax TA

### Optimized SPE+V-ITEX GC-MS procedure

1258 µL of urine sample was pre-concentrated using spherical, hydrophobic polystyrene-divinylbenzene copolymer SPE cartridges (Chromabond HR-X, 45 µm, 1 mL, 30 mg) mounted on a 24-position vacuum manifold (Chromabond, Machery-Nagel, Oensingen, Switzerland). Cartridges were preconditioned with three sequential rinses of 1 mL each of hexane, acetonitrile, Milli-Q water, and 4% H_3_PO_4_, all drawn through the sorbent using vacuum. For the optimized SPE+V-ITEX workflow based on DoE optimization, urine samples were pre-concentrated using dichloromethane as the elution solvent and airflow drying. Urine samples were acidified to 6% v/v with 85% v/v H_3_PO_4_ and left for 10 min in contact with the SPE cartridge material for extraction before applying suction. SPE eluates were subsequently analyzed by V-ITEX GC-MS using the same instrumental settings as for direct V-ITEX, except for the water removal step. Differences between workflows were limited to sample preparation and are summarized in Table 1 and Figure S1 in ESM.

### Design of experiment (DoE)

DoEs planning and evaluation were conducted with MODDE Pro® (version 13.0.2.34314, Sartorius, Tagelswangen, Switzerland). Two separate DoEs were developed, one for optimizing the direct V-ITEX of urine samples and the other for the SPE+V-ITEX extraction of urine samples. The design for the V-ITEX was a D-Optimal design with interactions, 29 runs, and 4 degrees of freedom. The design for the SPE+V-ITEX was also a D-Optimal design with interactions, 25 runs, and 4 degrees of freedom. The DoE approach was designed to efficiently screen parameter effects, and therefore did not include systematic replicates for each experimental condition. The corresponding DoE tables are provided in the ESM in Table S1 and Table S2. Optimal parameter settings were identified based on the mean values across all measurements as well as for the major compound groups. The Pearson correlation coefficient was calculated to analyze the relationships between the factors examined and the response variables. A *t*-test with a 95% confidence interval was used to assess statistical significance. A *p*-value < 0.05 was considered significant. Qualitative variables were first coded as binary variables using dummy coding so that they could be included in the correlation analysis. Each instance of a categorical factor was represented by a separate column with the values 0 (not present) or 1 (present).

### Optimization of V-ITEX

The first extraction approach evaluated was the direct extraction of urinary metabolites using V-ITEX. Four different critical parameters were optimized: the extraction time, extraction temperature, trap material, and desorption flow rate. The extraction time was tested at 0.5, 5.25, and 10 min. The extraction temperature was varied by 40, 65, and 90 °C, and the desorption flow rate was varied by 20, 200, and 400 mL/min. Five different commercial ITEX traps were tested (BGB Analytik AG, Böckten, Switzerland): Tenax TA/Carbosieve SIII (Tenax TA 80/100 mesh (2/3 bottom), Carbosieve SIII (1/3 top), 60/80 mesh), Tenax TA (80/100), Tenax TA/Carboxen (Silconert 2000, Tenax TA 50% bottom, Carboxen 1000 50% top), Carbosieve SIII (Silconert 2000, Carbosieve SIII 80/100 mesh), and Carboxen (Silconert 2000, Carbosieve SIII 80/100 mesh). A total of 200 µL of the urine pool sample was pipetted into a 20-mL headspace vial and sealed with a crimp cap.

### Optimization of SPE

Several critical parameters were optimized via DoE: the concentration of acid mixed with the samples (acidification of samples), the drying technique of the loaded SPE cartridges, the SPE extraction time (contact time before vacuum application), and the elution solvent. The sample acidification was varied to a final amount of 0%, 3%, and 6% v/v H_3_PO_4_ in the samples; therefore, two acid solutions were prepared (50 v/v% H_3_PO_4_ and 85 v/v% H_3_PO_4_) for mixing 1258 µL urine sample with 95 µL acid solution. For the 0% acid concentration, 95 µL of Milli-Q water was added instead. Two drying techniques were tested. In the first approach, the fume hood was closed, and the cartridges remained mounted on the vacuum manifold while lab air was drawn through them for 20 min. In the second method, the manifold was sealed using clamps and connected to a nitrogen gas line. Proper N_2_ flow was verified by observing bubble formation in an empty SPE cartridge filled with solvent (see Figure S2 in the ESM). The SPE extraction time, defined as the duration of sample-sorbent contact before vacuum was applied, was varied: 0.5, 5.25, and 10 min. Four elution solvents were evaluated: acetonitrile, acetone, ethyl acetate, and dichloromethane. A volume of 600 µL of each solvent was used to elute the sorbed analytes, and the eluates were stored at −80 °C. For analysis, 100 µL of eluate was pipetted into 20-mL headspace vials, sealed with a crimp cap, and kept in a cooled autosampler tray holder (4 °C). V-ITEX was performed on the eluates with a Tenax TA trap and constant values of 10 min incubation, 10 min extraction at 80 °C temperature, and 400 mL/min desorption flow with the carrier gas (remaining parameters please take from the V-ITEX section). We use the term SPE+V-ITEX to refer to the complete extraction workflow, whereas SPE refers exclusively to the solid-phase extraction step.

### Determination of limits of detection and limits of quantification

Limits of detection (LOD) and quantification (LOQ) were determined for ten representative compounds covering different chemical classes (benzene, 1,3-dimethyl-; styrene; heptanal; d-limonene; 1-octanol; 2-decanone; dodecane; 2-coumaranone; dodecanal; diethyl phthalate). The QC was aliquoted into seven 10-mL volumetric flasks and spiked with a standard mixture to obtain final concentrations of 0, 0.01, 0.03, 0.1, 0.3, 1, and 3 mg/L. The standard mixture was prepared from individual stock solutions (1 g/L) to yield a combined working solution of 50 mg/L. For each concentration level, aliquots were subjected either to direct V-ITEX analysis or to SPE+V-ITEX, as described above. Each of the seven calibration levels was determined in five replicates.

Peak areas of the spiked samples were subtracted by the corresponding unspiked QC signal to obtain net responses. Calibration curves were constructed considering only data points with signals exceeding the background level. LOD and LOQ were estimated based on the standard deviation of the response at the lowest calibration level and the slope of the calibration curve according to: $\mathrm{LOD}= \frac{3\sigma }{S}$ and $\mathrm{LOQ}= \frac{10\sigma }{S}$

where σ represents the standard deviation of replicate responses at the lowest concentration level and S the slope of the calibration curve.

### Greenness evaluation of the methods

The greenness of the analytical methods was evaluated using the Analytical Greenness Metric (AGREE) [18] and the Analytical Greenness Metric for Sample Preparation (AGREEprep) [19]. Both tools were applied using default weighting. The method-specific parameters and assumptions used for the evaluation are summarized in Table S3 in the ESM. The evaluation was performed based on each method’s optimized parameters, as determined through the DoE.

## Results and discussion

### Optimization of SPE

#### Elution solvent

**Fig. 1:**
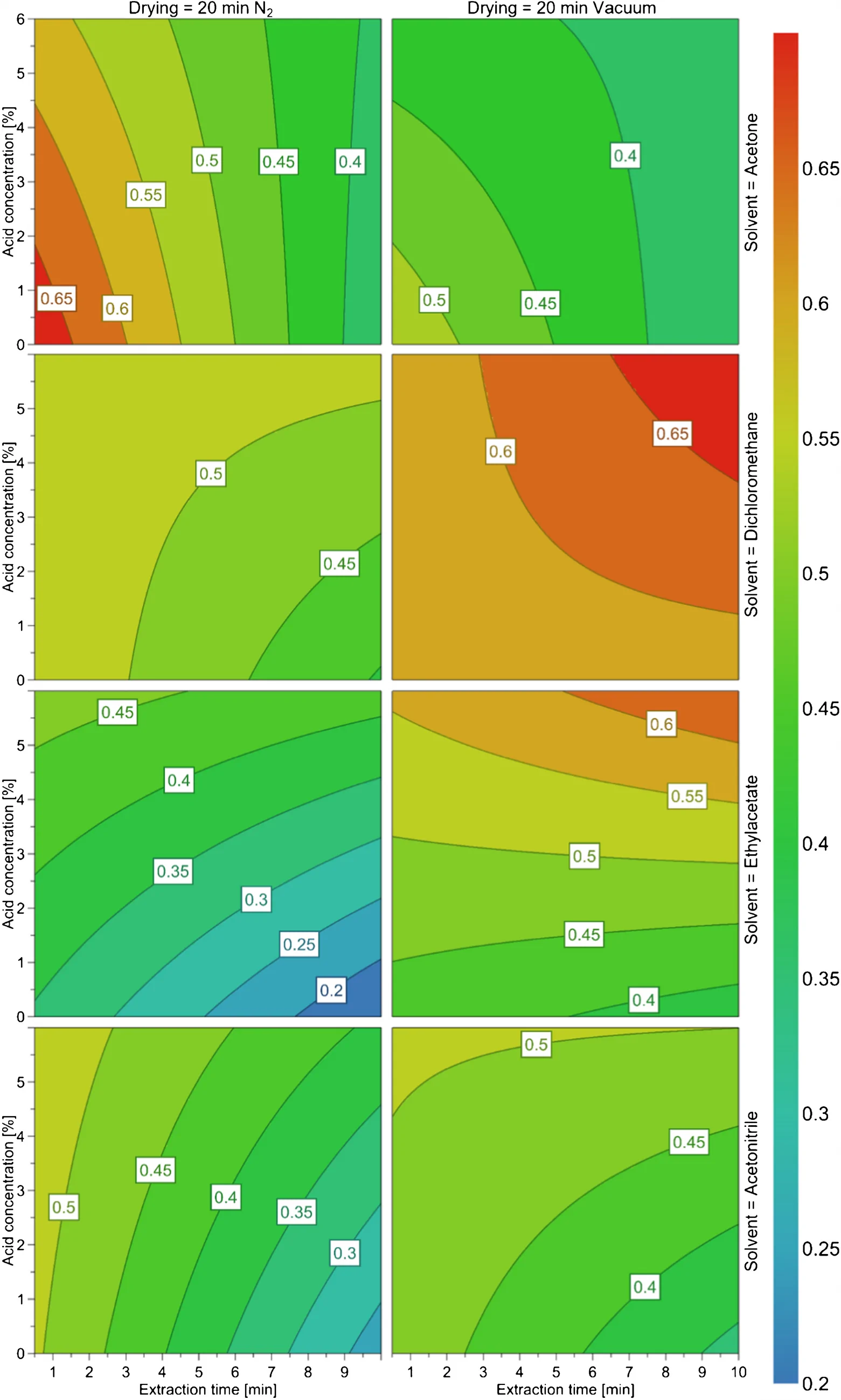
Contour plots of SPE procedure optimization determined by DoE. The summed peak area of all compounds is presented

Based on the DoE-based optimization of the SPE procedure, contour plots were generated to illustrate the interdependence of the investigated parameters. The contour plot showing the summed peak area of all identified compounds is presented in Fig. 1, while plots for individual compound groups are provided in the ESM (Figures S3 to S18). The optimal parameter settings are summarized in Table 1. In solid-phase extraction, the elution solvent plays a crucial role in removing the retained metabolites from the sorbent material, significantly influencing extraction efficiency and overall analytical performance. For the DoE, four elution solvents with varying polarities were evaluated as reflected by their log(*K*_ow_) values: −0.34 for acetonitrile, −0.23 for acetone, 0.68 for ethyl acetate, and 1.25 for dichloromethane [20]. Among the solvents tested, dichloromethane exhibited the best overall extraction performance when used with HR-X SPE cartridges, both in terms of the total response of all identified metabolites and for 50% of the individual compound groups. This finding is consistent with previous studies, where dichloromethane was also identified as the most efficient solvent for SPE-based extraction of aroma compounds from alcoholic beverages [21]. However, the optimal solvent choice varied depending on the compound group, which is evident in the contour plots provided in the ESM (Figures S3–S18). For instance, alcohols, ketones, nitriles, and terpenes were most efficiently extracted using acetone; esters and lactones with ethyl acetate; and hetero aromats and substituted aromats with acetonitrile. These preferences can be explained by structural similarity or polarity alignment between the solvent and the target analytes. Table 1 provides a practical reference for selecting optimal SPE conditions in future studies targeting specific compound groups.

#### Drying procedure

When working with aqueous samples in the SPE procedure, it is essential to dry the cartridges after sample loading due to the polymer-based resin used (manufacturers’ recommendations). In this study, two drying methods were evaluated over a 20-min period: nitrogen (N_2_) stream and airflow drying using vacuum, as detailed in the “Materials and methods” section. The goal was to assess whether airflow drying significantly alters the number or intensity of extracted compounds compared to nitrogen drying.

Airflow drying consistently yielded higher overall signal intensities. Interestingly, for specific compound groups, a pattern emerged suggesting a correlation between the drying method and the elution solvent used subsequently. N_2_ drying was never optimal when combined with dichloromethane or ethyl acetate but proved more effective when paired with acetonitrile or acetone. A plausible explanation for this lies in the drying efficiency, as airflow drying, due to reduced pressure, likely removes more residual water from the SPE cartridges. By contrast, nitrogen drying, operating at atmospheric pressure or slight overpressure, is less effective in eliminating water. This difference becomes crucial depending on the polarity of the elution solvent. Acetonitrile and acetone are miscible with water, allowing efficient extraction, even though some moisture may remain. Conversely, dichloromethane has poor water solubility, and ethyl acetate is only moderately water soluble; thus, residual water on the sorbent can interfere with the interaction between these more hydrophobic solvents and the sorbed analytes, ultimately reducing extraction efficiency [22]. However, the correlation testing results showed no significant correlation between any solvent and any drying technique (range of *r* = −0.0144 to 0.1938; range of *p* = 0.353–0.946).

#### SPE extraction time

The SPE extraction time refers to the duration during which the solvent is in contact with the cartridge material before applying the vacuum. While the optimal extraction time was determined to be 10 min, individual compound groups showed varying preferences, ranging from the minimum tested time of 0.5 min to the maximum of 10 min. The reason for this cannot be explained based on the experiments conducted and may instead be the result of an interaction effect. As the extraction time can also depend on the elution solvent, a correlation was tested, but no significant correlation was found between the extraction time and the elution solvent (range of *r* = −0.0374 to 0.0419; range of *p* = 0.800–0.859).

#### Acid concentration

The addition of acid to the sample was done based on literature precedence to protonate carboxylic acids and enhance their volatility in headspace analysis [23]. The HR-X SPE material is stable over a wide pH range from pH 1–14 (manufacturers’ recommendations). The optimal acid concentration in the samples was 6% v/v. For the compound groups, it ranged from 0 to 6% v/v. The strongest significant negative correlation (*r* = −0.53, *p* = 0.0069) was observed between acidification and caprolactam, indicating that higher acid concentrations are associated with lower caprolactam levels. This finding is consistent with the known instability of lactams, as they can undergo hydrolysis under acidic conditions [24]. The only significant positive correlation (*r* = 0.44, *p* = 0.029) was found for substituted aromats, with four phenolic compounds likely contributing the most to this relationship. Phenols are protonated at higher acid concentrations, which facilitates their transfer into the gas phase.

### Optimization of V-ITEX

#### ITEX material

**Fig. 2:**
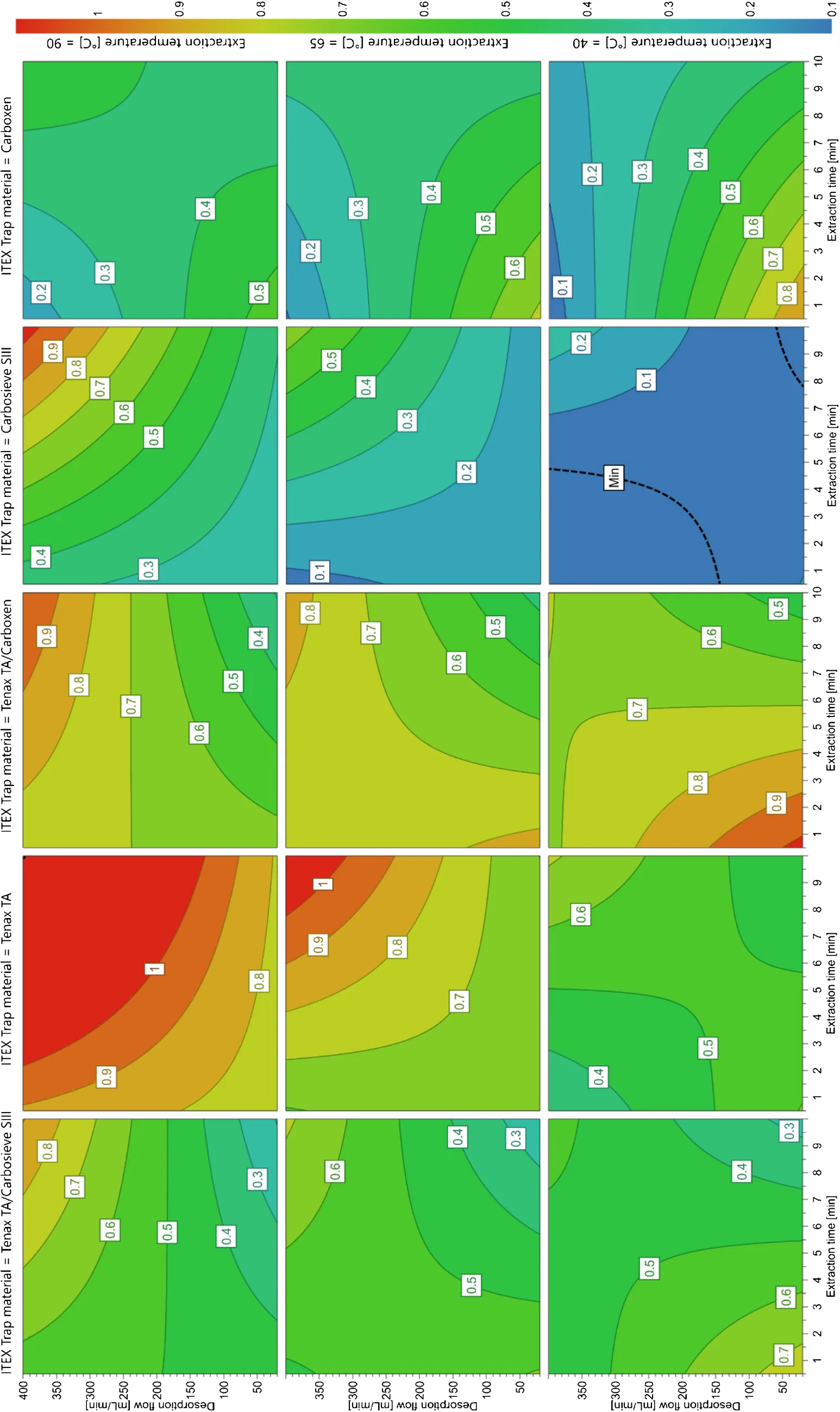
Contour plots of V-ITEX procedure optimization determined by DoE. The summed peak area of all compounds is presented

The contour plots of the DoE V-ITEX optimization are shown in Fig. 2, which displays the sum of all identified compounds. The contour plots of the groups are presented in the ESM (Figures S19–S34). Table 1 shows the optimal values for the different compound groups. Tenax TA, a non-polar polymer with low water affinity, demonstrated broad applicability, as it was optimal for 69% of the compound groups. By contrast, highly adsorptive materials such as Carboxen (found optimal for hetero aromats, phthalates, and dimethyl sulfone) and Carbosieve SIII (found optimal for cyclic hydrocarbons), although offering high surface areas, showed selective advantages only for specific polar or small cyclic compounds, which limits their general applicability. The observed unsuitability of the Tenax TA/Carbosieve SIII combination may be attributed to competitive adsorption and inconsistent desorption efficiency.

#### Extraction time and temperature

The optimal extraction conditions were 10 min and 90 °C. Most compounds and groups benefited from the longest time and highest temperature; however, aliphatic hydrocarbons, azines (1,3,5-tri-2-propen-1-yl-1,3,5-triazine-2,4,6(1H,3H,5H)-trione), heteroaromatics, caprolactam, phthalates, and dimethyl sulfone showed optimal extraction at the shortest time (0.5 min), and several of these also at the lowest temperature (40 °C). No clear pattern among these compound types was identified. These mild conditions may result from trap saturation, analyte breakthrough, or vacuum effects (5 mbar), particularly affecting dimethyl sulfone (see Figure S33 in the ESM). Contour plots (ESM Figures S19–S34 and Table 1) show a strong influence of trap material: Tenax TA and Carbosieve SIII favored 90 °C and 10 min, while Carboxen and Tenax TA/Carboxen consistently performed best at 40 °C and 0.5 min. The preference of Carboxen-based traps for low temperature and short extraction time may be attributed to their high adsorption capacity and microporous structure, which leads to early saturation, competitive adsorption, and potential analyte breakthrough under extended extraction or elevated temperatures [25]. Additionally, increased water vapor at higher temperatures may block active sites, especially in moisture-sensitive Carboxen materials [26]. These combined effects could explain the observed behavior, although further studies are required for full mechanistic clarification.

#### Desorption flow

The desorption flow in the V-ITEX procedure is a critical parameter that influences not only the desorption efficiency of the analytes but also the quality of subsequent chromatographic separation. The overall optimal desorption flow was found to be 400 mL min^−1^. However, for certain compound groups, namely cyclic hydrocarbons, hetero aromats, phthalates, and dimethyl sulfone, lower desorption flows in the range of 20–40 mL min^−1^ were optimal. A possible relationship between desorption flow and trap material was observed. For Tenax TA and Tenax TA/Carboxen, the optimal desorption flow was the maximal value 400 mL min^−1^, whereas for Carboxen and Carbosieve III, the value ranged from 20 to 40 mL min^−1^. Despite this trend, no statistically significant correlation between any trap material and desorption flow was confirmed (range of *r* = −0.0794 to 0.1158; range of *p* = 0.550–0.933).

### Identified compounds and possible metabolic origins

The analysis of volatile metabolites detected in the urine of healthy males revealed a chemically diverse set of compounds with both endogenous and exogenous origins. This study focused exclusively on compounds with confidence levels of 1 and 2 in accordance with the criteria defined by the Chemical Analysis Working Group of the Metabolomics Standards Initiative [16, 17], thereby focusing on the most substantiated findings. Most compounds were identified with a confidence level of 2, and 28 compounds were assigned to level 1 due to confirmation with a standard mixture. A comprehensive overview of the putatively annotated compounds and their key analytical parameters is presented in Table 2, and an overview of the compounds’ possible sources according to the Human Metabolome Database [27] is presented in Table S4 in the ESM. Compounds with both endogenous and exogenous origins are particularly common among major chemical classes, such as aldehydes, alcohols, and ketones. These volatiles can arise from internal metabolic processes, such as lipid peroxidation and amino acid degradation, as well as from external sources, such as food, beverages, and environmental exposure [28–30]. Representative examples include 1-octanol, hexanal, and 4-heptanone which exemplify this biochemical overlap. Notably, compounds such as phenol and 4-ethylphenol, which were also detected, are recognized as microbial fermentation products, likely derived from gut microbiota. By contrast, certain compounds, such as terpenes (e.g., limonene, cadalene), appear to be exclusively of exogenous origin, typically linked to the consumption of herbs, spices, and citrus fruits [31]. Another important subgroup includes ketones (e.g., 2-pentanone and 3-heptanone), which have been observed to reflect endogenous lipid metabolism, particularly ketogenesis. The presence of these compounds in healthy individuals indicates normal baseline production. However, altered levels could serve as indicators of metabolic shifts, such as starvation, a ketogenic diet, or prolonged physical exercise [30]. Ketones can also originate from exogenous sources, including but not limited to animal- or plant-based foods such as vegetables, fruits, cereals, herbs, beverages, processed foods, and synthetic sources (see Table S4 in the ESM). A total of 55 compounds could not be matched to existing HMDB entries, although they were structurally assigned with identification level 1 or 2 according to the Metabolomics Standards Initiative. The absence of HMDB annotation indicates missing metabolic context rather than unknown chemical identity and is likely related to the limited coverage of volatile metabolites in predominantly LC-MS-based databases.

**Table 2:** List of putatively annotated compounds (confidence levels 1 and 2) with their most important analytical parameters. For the compounds identified with level 1, an analytical standard was measured for confirmation. ^a^Compound not listed in Human Metabolome Database; *RT*, retention time in min; *CAS no.*, Chemical Abstracts Service number; *RI sample*, retention index obtained in measurements of real samples; *RI std*, retention index measured for analytical standard; *RI NIST*, retention index obtained from NIST database for a non-polar column type; *Quant. m/z*, quantifier ion; *Qual. m/z*, qualifier ion; *MF NIST*, match factor from NIST database in %; *Id. Lvl.*, identification level according to the Metabolomics Standards Initiative [16, 17]

Group	NIST name	RT	CAS no.	RI sample	RI std	RI NIST	Quant. m/z	Qual. m/z	MF NIST	Id. Lvl.
Alcohols	1-Hexanol, 2-ethyl-	11.94	104-76-7	1029	nd	1030	57	70	97	2
	Benzyl alcohol	12.08	100-51-6	1037	nd	1036	79	108	98	2
	1-Octanol	12.73	111-87-5	1073	1075	1070	56	84	96	1
	2-Phenyl-2-propanol^a^	13.00	617-94-7	1088	nd	1090	121	118	95	2
	Ethanol, 2-phenoxy-	15.14	122-99-6	1222	nd	1225	94	138	96	2
Aldehydes	Hexanal	6.89	66-25-1	801	nd	801	56	72	95	2
	Furfural	7.68	98-01-1	833	835	833	96	95	92	1
	Heptanal	9.37	111-71-7	902	902	901	70	55	98	1
	Benzaldehyde	10.66	100-52-7	964	nd	962	77	106	99	2
	Octanal	11.49	124-13-0	1004	1003	1003	43	84	99	1
	Benzeneacetaldehyde	12.25	122-78-1	1046	1047	1045	91	120	95	1
	Nonanal	13.29	124-19-6	1104	1105	1104	57	98	99	1
	Benzaldehyde, 2-ethyl-^a^	14.00	22927−13-5	1149	nd	1164	133	105	97	2
	2-Nonenal, (E)-	14.19	18829-56-6	1161	nd	1162	41	70	94	2
	Decanal	14.90	112-31-2	1206	nd	1206	43	70	99	2
	Benzaldehyde, 2,4-dimethyl-	15.13	15764-16-6	1221	nd	1208	133	105	98	2
	Undecanal	16.38	112-44-7	1307	nd	1307	57	82	95	2
	Dodecanal	17.77	112-54-9	1409	1410	1409	57	82	99	1
	Tetradecanal	20.30	124-25-4	1614	nd	1613	57	82	98	2
	3,5-di-tert-Butyl-4-hydroxybenzaldehyde	22.00	1620-98-0	1766	nd	1772	219	191	87	2
Aliphatic hydrocarbons	1-Octene	6.66	111-66-0	792	nd	789	55	70	96	2
	3-Heptene, 2,2,4,6,6-pentamethyl-	11.55	123-48-8	1007	nd	1006	57	97	87	2
	Nonane, 2,5-dimethyl-^a^	11.77	17302-27-1	1020	nd	1021	57	85	92	2
	Octane, 2,3,6,7-tetramethyl-^a^	13.09	52670-34-5	1093	nd	1107	71	121	93	2
	Undecane	13.22	1120-21-4	1100	1100	1100	57	71	96	1
	Undecane, 3-methyl-^a^	14.35	1002-43-3	1171	nd	1170	57	71	93	2
	Dodecane	14.82	112-40-3	1200	1200	1200	57	71	98	1
	Dodecane, 2-methyl-	15.77	1560-97-0	1265	nd	1264	57	71	87	2
	Dodecane, 3-methyl-^a^	15.87	17312-57-1	1272	nd	1271	57	71	84	2
	Tridecane	16.28	629-50-5	1300	1300	1300	57	71	98	1
	Dodecane, 4,6-dimethyl-^a^	16.58	61141-72-8	1322	nd	1325	57	71	95	2
	Tridecane, 3-methyl-^a^	17.25	6418-41-3	1371	nd	1371	57	71	87	2
	Tetradecane	17.64	629-59-4	1399	1400	1400	57	71	98	1
	Cyclopentane, nonyl-^a^	18.33	2882-98-6	1453	nd	1452	69	196	92	2
	5,5-Diethyltridecane^a^	20.15	5925-55-3	1601	nd	1605	57	85	82	2
	Heptadecane	21.28	629-78-7	1700	1700	1700	57	71	93	1
Aromatic hydrocarbons	Toluene	6.03	108-88-3	767	nd	764	91	65	98	2
	Ethylbenzene	8.40	100-41-4	863	nd	855	91	106	93	2
	Benzene, 1,3-dimethyl-^a^	8.63	108-38-3	872	871	866	91	106	95	1
	Styrene	9.13	100-42-5	892	892	893	104	78	98	1
	Benzene, 1-methyl-3-(1-methylethenyl)-	13.08	1124-20-5	1093	nd	1082	132	117	94	2
	Benzene, 1,2,4,5-tetramethyl-^a^	13.58	95-93-2	1123	nd	1115	119	134	96	2
	Benzene, pentyl-^a^	14.18	538-68-1	1160	nd	1157	91	148	85	2
	Naphthalene	14.71	91-20-3	1193	nd	1182	128	102	99	2
	Benzene, 1,2,4-triethyl-^a^	15.09	877-44-1	1219	nd	1207	147	133	94	2
	1H-Indene, 2,3-dihydro-1,1,3-trimethyl-^a^	15.25	2613-76-5	1230	nd	1242	145	160	92	2
	Benzene, 1,3-bis(1,1-dimethylethyl)-^a^	15.57	1014-60-4	1251	nd	1249	175	57	87	2
	Naphthalene, 2-methyl-^a^	16.38	91-57-6	1307	nd	1297	142	115	88	2
	Naphthalene, 1,4-dimethyl-^a^	18.12	571-58-4	1437	nd	1436	156	141	94	2
	Benzene, 1-(1,5-dimethyl-4-hexenyl)−4-methyl-^a^	18.75	644-30-4	1486	nd	1483	119	202	88	2
	1H-Indene, 2,3-dihydro-1,1,3-trimethyl-3-phenyl-^a^	21.54	3910-35-8	1724	nd	1714	221	143	91	2
	2,6-Diisopropylnaphthalene	21.63	24157-81-1	1732	1737	1728	197	155	90	1
	Benzene, (1-butyloctyl)-^a^	21.65	2719-63-3	1734	nd	1730	91	147	87	2
	Benzene, (1-pentyloctyl)-^a^	22.65	4534-49-0	1828	nd	1819	91	105	84	2
Azine	1,3,5-Triazine-2,4,6(1H,3H,5H)-trione, 1,3,5-tri-2-propen-1-yl-^a^	20.82	1025-15-6	1660	nd	1661	83	249	94	2
Cyclic hydrocarbons	2-Bornene^a^	9.38	464-17-5	903	nd	908	93	121	93	2
	3-Thujene	10.08	3917-48-4	936	nd	929	93	77	92	2
Esters	3-Methylcyclopentyl acetate^a^	9.33	24070-70-0	901	nd	905	72	84	89	2
	n-Butyl methacrylate	11.00	97-88-1	980	nd	989	69	87	90	2
	2-Propenoic acid, 2-methyl-, 2-hydroxypropyl ester^a^	12.17	923-26-2	1042	nd	1051	69	100	87	2
	Benzoic acid, ethyl ester	14.40	93-89-0	1174	nd	1171	105	122	92	2
	Methyl salicylate	14.77	119-36-8	1197	1197	1192	120	152	94	1
	Methacrylic acid, tetrahydrofurfuryl ester^a^	15.53	2455-24-5	1248	nd	1254	71	84	97	2
	Isopentyl methacrylate^a^	16.96	30421-56-8	1350	nd	1353	109	124	96	2
	3-Hydroxy-2,2,4-trimethylpentyl isobutyrate^a^	17.33	25265-77-4	1377	nd	1374	71	89	99	2
	Exo-1,7,7-trimethylbicyclo[2.2.1]hept-2-yl isobutyrate^a^	17.42	85586-67-0	1383	nd	1390	95	136	99	2
	2,2,4-Trimethyl-1,3-pentanediol diisobutyrate	20.02	6846-50-0	1590	1590	1588	71	243	94	1
	2-Ethylhexyl salicylate^a^	22.48	118-60-5	1811	nd	1807	120	138	97	2
	Isopropyl myristate^a^	22.59	110-27-0	1822	nd	1827	102	60	98	2
	1,2-Benzenedicarboxylic acid, bis(2-methylpropyl) ester^a^	23.02	84-69-5	1863	nd	1869	149	223	98	2
	Isopropyl palmitate	24.59	142-91-6	2022	nd	2024	256	60	97	2
Hetero aromats	Pyrrole	5.61	109-97-7	751	nd	755	67	41	97	2
	Benzofuran	11.35	271-89-6	997	997	1000	118	90	86	1
	Benzofuran, 2,3-dihydro-	15.05	496-16-2	1216	nd	1224	120	91	88	2
	Coumarin	18.29	91-64-5	1450	nd	1440	118	146	94	2
Ketones	3-Penten-2-one	5.31	625-33-2	739	nd	733	69	84	91	2
	3-Hexanone	6.47	589-38-8	785	784	784	57	71	88	1
	3-Hexanone, 2-methyl-	6.73	7379-12-6	795	nd	784	71	114	87	2
	Cyclopentanone, 3-methyl-^a^	8.12	1757-42-2	851	nd	851	69	98	93	2
	4-Heptanone	8.68	123-19-3	874	nd	872	71	114	98	2
	4-Cyclopentene-1,3-dione^a^	8.85	930-60-9	881	nd	883	96	68	95	2
	2-Heptanone	9.08	110-43-0	890	891	891	58	71	89	1
	2-Heptanone, 4-methyl-^a^	10.13	6137-06-0	939	nd	943	43	84	94	2
	3-Ethylcyclopentanone^a^	10.60	10264-55-8	961	nd	962	83	112	93	2
	2,3-Octanedione	11.11	585-25-1	985	nd	984	43	99	84	2
	Acetophenone	12.66	98-86-2	1069	nd	1066	105	120	98	2
	4-Acetyl-1-methylcyclohexene	13.76	6090-09-1	1134	nd	1137	138	95	89	2
	2,6,6-Trimethyl-2-cyclohexene-1,4-dione	13.96	1125-21-9	1147	nd	1144	68	96	82	2
	Ethanone, 1-(4-methylphenyl)-	14.65	122-00-9	1190	nd	1183	119	91	91	2
	2-Decanone	14.68	693-54-9	1191	1192	1193	58	71	89	1
	2-Methoxyphenylacetone^a^	16.43	5211-62-1	1311	nd	1318	91	121	86	2
	2-Propanone, 1-(4-methoxyphenyl)-	16.44	122-84-9	1312	nd	1318	121	164	87	2
	5,9-Undecadien-2-one, 6,10-dimethyl-	18.27	3796-70-1	1448	nd	1455	43	69	97	2
	Benzophenone	20.63	119-61-9	1643	1641	1635	105	182	92	1
	7-Pentadecanone	20.97	6064-38-6	1672	nd	1658	43	58	92	2
	Ethanone, 2,2-dimethoxy-1,2-diphenyl-^a^	23.28	24650-42-8	1889	nd	1874	151	105	94	2
Lactam	Caprolactam	15.65	24650-42-8	1257	nd	1253	113	84	99	2
Lactones	γ-Crotonolactone	9.50	497-23-4	909	nd	920	55	84	87	2
	γ-Butyrolactone	9.54	96-48-0	911	nd	915	42	86	98	2
	γ-Pentalactone^a^	10.42	108-29-2	953	nd	956	56	85	94	2
	4-Methyl-4-vinylbutyrolactone	12.12	1073-11-6	1039	nd	1043	111	126	94	2
	γ-Hexalactone	12.37	695-06-7	1053	nd	1056	85	70	91	2
	δ-Hexalactone	13.10	823-22-3	1094	nd	1095	70	55	82	2
	2-Coumaranone^a^	15.38	553-86-6	1239	1240	1249	78	134	80	1
	γ-Octalactone	15.67	104-50-7	1258	1259	1261	85	100	88	1
	γ-Nonalactone^a^	17.15	104-61-0	1363	1364	1364	58	128	94	1
	Dodecanolactone^a^	19.52	947-05-7	1549	nd	1540	55	98	82	2
Nitriles	Isoamyl cyanide^a^	7.98	542-54-1	846	nd	847	55	82	89	2
	Allyl Isothiocyanate	8.87	57-06-7	882	nd	885	99	72	94	2
	Benzonitrile^a^	11.13	100-47-0	986	nd	984	103	76	90	2
	Cyclohexane, isothiocyanato-^a^	15.41	1122-82-3	1240	nd	1251	141	83	98	2
	Dodecanenitrile^a^	18.79	2437-25-4	1489	nd	1490	97	110	93	2
Phthalates	Diethyl phthalate	20.01	84-66-2	1590	1590	1594	149	177	92	1
	Dibutyl phthalate	23.98	84-74-2	1959	nd	1965	149	223	98	2
Substituted aromats	Benzene, chloro-	8.04	108-90-7	848	nd	852	112	77	91	2
	Phenol	11.00	108-95-2	980	nd	981	94	66	99	2
	p-Cresol	12.74	106-44-5	1074	nd	1077	107	77	96	2
	Formamide, N-phenyl-^a^	15.16	103-70-8	1223	nd	1221	121	93	90	2
	Phenol, m-tert-butyl-^a^	16.16	585-34-2	1292	nd	1296	135	150	86	2
	2-Methoxy-4-vinylphenol	16.49	7786-61-0	1315	1315	1316	150	135	96	1
	Phenol, 5-ethenyl-2-methoxy-	16.72	621-58-9	1332	nd	1337	135	150	88	2
	Benzene, (2-isothiocyanatoethyl)-	18.58	2257-09-2	1473	nd	1469	91	163	95	2
	2,4-Di-tert-butylphenol	19.01	96-76-4	1507	nd	1514	191	206	99	2
	Phenol, 3,5-bis(1,1-dimethylethyl)-^a^	19.30	1138-52-9	1531	nd	1527	191	206	91	2
Sulfone	Dimethyl sulfone	9.59	67–71-0	913	nd	922	79	94	98	2
Terpenes	Frontaline^a^	10.25	60478-96-8	944	nd	949	43	72	86	2
	Camphene	10.46	79–92-5	954	nd	951	93	121	98	2
	3-Carene	11.62	13466-78-9	1011	nd	1010	93	136	96	2
	p-Cymene	11.91	99-87-6	1027	1027	1025	119	134	97	1
	d-Limonene	12.00	5989-27-5	1032	1032	1031	68	93	91	1
	Eucalyptol	12.07	470-82-6	1036	nd	1032	81	154	90	2
	Camphor	14.08	76-22-2	1154	nd	1144	95	121	98	2
	Terpinen-4-ol	14.59	562-74-3	1186	1183	1182	71	111	89	1
	Carvone	15.54	562-74-3	1249	nd	1246	82	108	97	2
	Piperitone	15.70	89-81-6	1261	nd	1253	82	110	87	2
	Longifolene	17.99	475-20-7	1427	nd	1415	161	91	92	2
	γ-Muurolene	18.75	30021-74-0	1486	nd	1477	161	204	94	2
	10,11-Epoxycalamenene^a^	18.89	143785-42-6	1497	nd	1493	173	158	91	2
	δ-Cadinene	19.27	483-76-1	1528	nd	1524	91	134	92	2
	trans-Calamenene^a^	19.33	73209-42-4	1533	nd	1529	159	202	91	2
	Cadina-1(10),6,8-triene^a^	19.52	1460-96-4	1549	nd	1556	187	159	89	2
	α-Calacorene	19.58	21391-99-1	1554	nd	1542	157	142	92	2
	Cadalene	21.13	483-78-3	1687	nd	1674	183	198	85	2

### SPE+V-ITEX and V-ITEX procedure comparison

#### Method validation and performance evaluation

Method validation was conducted with the optimized methods using a set of selected compounds identified with confidence level 1 (see Table 3). Intra-day repeatability and inter-day repeatability were assessed based on five replicate measurements of pooled QC urine samples. No outliers were removed from the dataset in order to realistically reflect the inherent variability of each method. Prior to statistical evaluation, the data were normalized to the sum of all selected peak areas per sample.

**Table 3:** Results of inter-day and intra-day repeatability for compounds identified with confidence level 1. Repeatability was measured by the standard deviation of each five replicates of pooled QC samples

Compound name	Intra-day rep. (%) SPE+V-ITEX	Inter-day rep. (%) SPE+V-ITEX	Intra-day rep. (%) V-ITEX	Inter-day rep. (%) V-ITEX
3-Hexanone	13	30	12	14
Furfural	14	18	21	23
Benzene, 1,3-dimethyl-	13	12	18	19
2-Heptanone	14	30	16	13
Styrene	15	6	35	20
Heptanal	6	30	10	28
Benzofuran	12	8	9	5
Octanal	8	21	23	18
p-Cymene	6	9	15	12
d-Limonene	2	14	42	16
Benzeneacetaldehyde	6	8	8	13
1-Octanol	31	74	37	35
Nonanal	26	24	45	36
2-Decanone	7	19	13	64
Methyl salicylate	4	8	0	0
2-Coumaranone	4	16	11	12
γ-Octalactone	7	15	15	11
2-Methoxy-4-vinylphenol	10	42	3	6
γ-Nonalactone	6	8	15	4
Dodecanal	3	14	13	19
2,2,4-Trimethyl-1,3-pentanediol diisobutyrate	17	22	13	5
Diethyl Phthalate	10	53	17	11
Benzophenone	17	18	6	9
2,6-Diisopropylnaphthalene	6	12	28	15

The results indicate that 76% of all reported values exhibited a relative standard deviation (RSD) below 20%, while 18% ranged between 20 and 40%, and 6% exceeded 40%. The highest variability was observed for 1-octanol, where an inter-day RSD of 74% was recorded. This pronounced fluctuation may be attributed to compound-specific variability rather than systematic differences between the extraction workflows.

The detection capability of the developed workflows was evaluated by determining LODs and LOQs in a matrix-matched calibration for representative compounds covering different chemical classes. The results are presented in Table 4. The obtained LOD values ranged from 5 to 721 µg/L for direct V-ITEX and from 17 to > 3000 µg/L for SPE+V-ITEX, while corresponding LOQ values ranged from 15 to 2400 µg/L and 56 to > 3000 µg/L, respectively. *R*^2^ values of the linear range ranged from 0.8762 to 0.9432 for direct V-ITEX and from 0.4864 to 0.9309 SPE+V-ITEX.

**Table 4:** Limits of detection (LOD), limits of quantification (LOQ), and regression coefficients of linear range (*R*^2^) for representative compounds obtained by GC-MS with direct V-ITEX and SPE+V-ITEX

Compound	LOD V-ITEX (µg/L)	LOQ V-ITEX (µg/L)	*R*^2^ V-ITEX	LOD SPE+V-ITEX (µg/L)	LOQ SPE+V-ITEX (µg/L)	*R*^2^ SPE+V-ITEX
Benzene, 1,3-dimethyl-	11	35	0.9345	97	325	0.8761
Styrene	30	99	0.9246	26	86	0.8573
Heptanal	17	56	0.9425	17	56	0.8512
d-Limonene	5	15	0.9432	102	339	0.8688
1-Octanol	178	595	0.8762	> 3000	> 3000	0.7388
2-Decanone	16	52	0.8935	37	122	0.9080
Dodecane	6	21	0.9206	1140	> 3000	0.9309
2-Coumaranone	20	65	0.9356	32	107	0.9196
Dodecanal	721	2400	0.9099	> 3000	> 3000	0.4864
Diethyl phthalate	46	155	0.9053	89	297	0.8659

Compound-specific differences were observed, with lower LOD values obtained for analytes exhibiting low or negligible background levels in the QC, such as d-limonene, styrene, heptanal, and 2-decanone. For certain compounds, such as dodecanal and 1-octanol, LOD and LOQ values could not be reliably determined within the investigated concentration range (> 3000 µg/L) and showed insufficient linearity (*R*^2^ < 0.85). This behavior can be attributed to increased signal variability associated with higher background intensities, which directly influences the standard deviation used for LOD estimation and linearity in this matrix-matched approach.

These findings highlight that, in matrix-matched approaches, LODs, LOQs, and linearity are not solely governed by instrumental sensitivity but are also influenced by compound-specific matrix contributions. Despite this, the obtained LODs demonstrate that both workflows are suitable for the detection of low-abundance volatile compounds in urine within the investigated concentration range.

#### Applicability to real samples

**Fig. 3:**
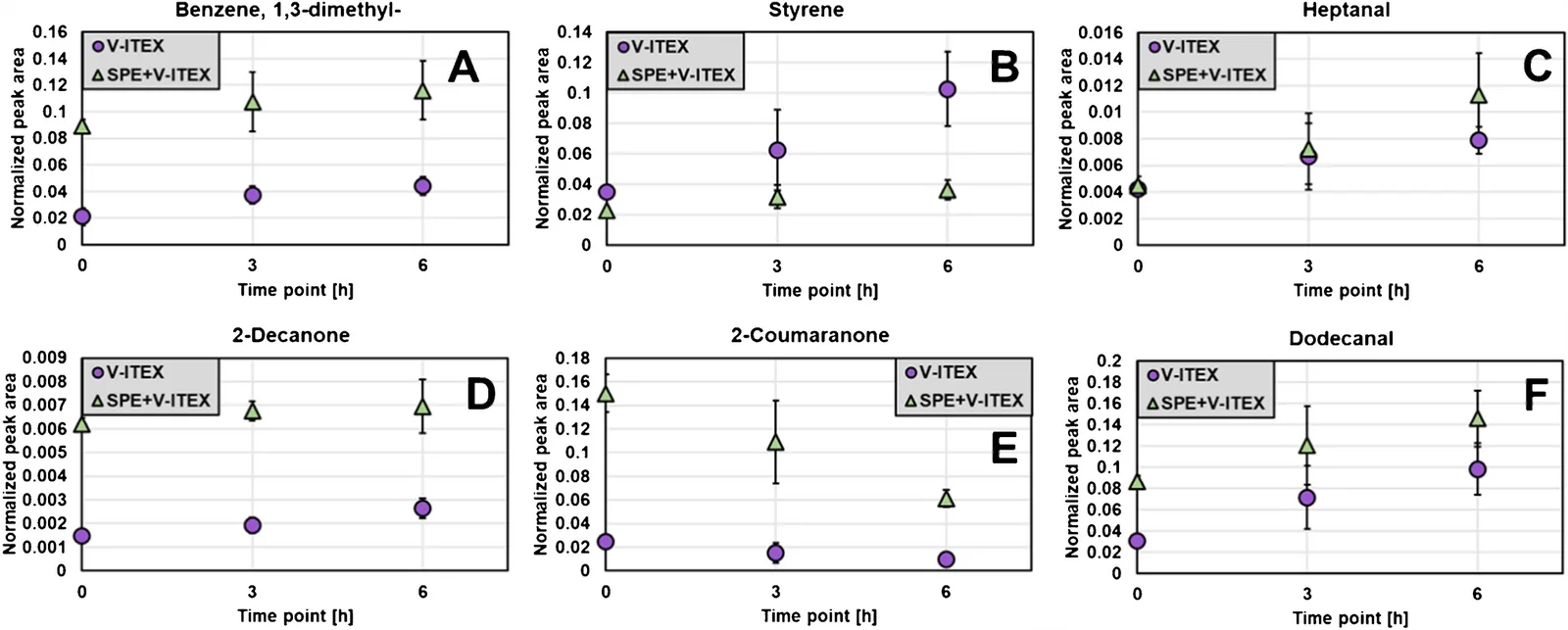
Temporal profiles of six representative volatile urine metabolites measured at three time points (t_0_, t_3_, t_6_) using direct V-ITEX (circles) and SPE+V-ITEX (triangles). Shown are normalized mean peak areas ± standard deviation (*n* = 3 per time point). **A** Benzene, 1,3-dimethyl-; **B** Styrene; **C** Heptanal; **D** 2-Decanone; **E** 2-Coumaranone; **F** Dodecanal. Temporal variation of normalized compound peak area at the three different time points t0, t3, and t6 of pooled samples measured in triplicates with the optimized methods

To assess the applicability of the developed methods to real biological samples, pooled QC urine samples corresponding to the three time points (t_0_, t_3_, and t_6_) were analyzed in triplicate using both workflows. Figure 3 shows six representative compounds that exhibited temporal variation across the intervals. Notably, the standard deviations were broadly comparable between the two methods and the temporal trajectories were similar in most cases, although differences in slope between methods were sometimes evident. For example, for styrene (Fig. 3B), both methods reflect a positive postprandial increase, consistent with previous observations in serum after a high-fat meal [15]. In contrast, for 2-coumaranone (Fig. 3E), concentrations decreased over time; this aligns with literature findings showing no significant postprandial effect after milk intake, although age-related differences have been reported in literature [3]. For 1,3-dimethylbenzene, no dietary association was identified in literature that could explain the increase detected in this study. Ketones such as 2-decanone (Fig. 3D) can originate from both endogenous lipid metabolism (e.g., β-oxidation and ketogenesis) and exogenous sources including dairy products [15, 32]. Finally, aldehydes, which are known to derive from both endogenous lipid peroxidation and exogenous food and environmental sources (e.g., cooking, microbial fermentation) showed coherent time-dependent behavior (Fig. 3C and F) [33]. The fact that both extraction methods detected comparable profile changes demonstrates that they function adequately in real-sample contexts and are capable of tracking volatile metabolite kinetics in urine.

A limitation of this study for deeper metabolic interpretation is the small and homogeneous sample set, comprising urine from four healthy male individuals. However, the primary aim of this work was not biological interpretation or population-wide generalization of urinary metabolite profiles, but the methodological comparison and optimization of two extraction workflows under realistic matrix conditions. Consequently, the conclusions of this study are restricted to the analytical performance of the extraction methods, and not to sex-, age-, or disease-specific metabolic patterns. Future studies applying the optimized workflows to larger and more diverse cohorts will be required to evaluate biological variability and clinical or nutritional relevance.

**Fig. 4:**
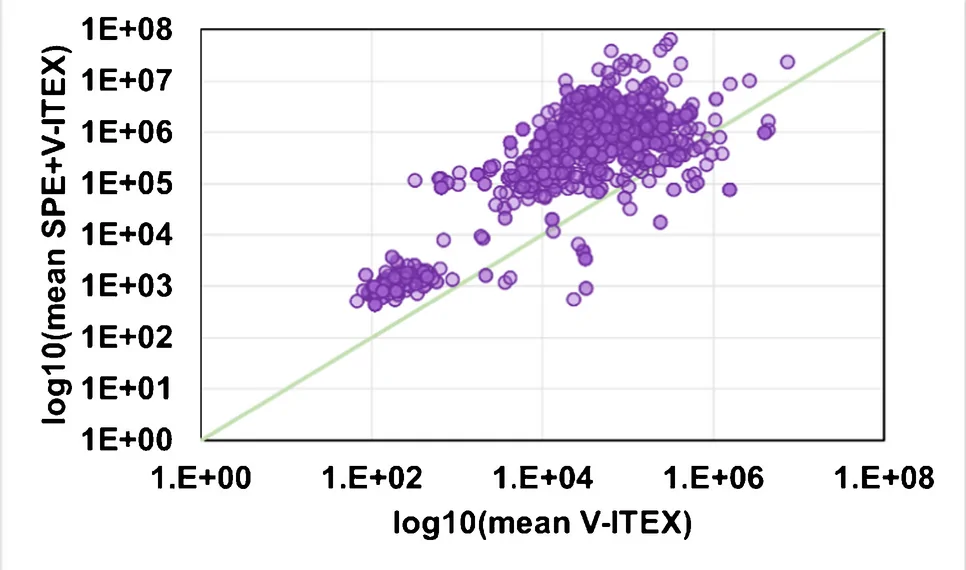
Logarithmic scatter plot comparing mean peak areas (log₁₀-transformed) of 1198 detected features obtained by direct V-ITEX and SPE+V-ITEX. Each point represents the average signal intensity of a feature across replicates. The diagonal line indicates equal response in both methods

Figure 4 illustrates the distribution of signal intensities for all 1198 detected features by Profinder software (version 10.0, Agilent, Basel, Switzerland) based on the average peak area across replicates for both extraction methods. A logarithmic scatter plot (log_10_-transformed mean peak areas) was used to visualize the dynamic range and the relationship between signal intensities obtained with direct V-ITEX and SPE+V-ITEX. Most features clustered within a well-defined linear region along the diagonal, indicating a generally proportional signal response between the two methods. However, a distinct group of features with relatively low intensities in the direct V-ITEX method (log_10_ < 3) but higher signals in SPE+V-ITEX is clearly visible, suggesting enhanced enrichment by the SPE pre-concentration step for this subset. This group likely represents low-abundance or poorly volatile compounds that benefit from solvent extraction. Additionally, it is conceivable that SPE introduces more chemical artifacts originating from sorbent interactions, solvents, or sample handling steps which may contribute to the overall higher number of detected features.

The overall distribution further confirms that while both methods capture the majority of features in a comparable dynamic range, method-specific differences in extraction efficiency and matrix handling can influence the detectability of certain compound classes.

#### Greenness

**Fig. 5:**
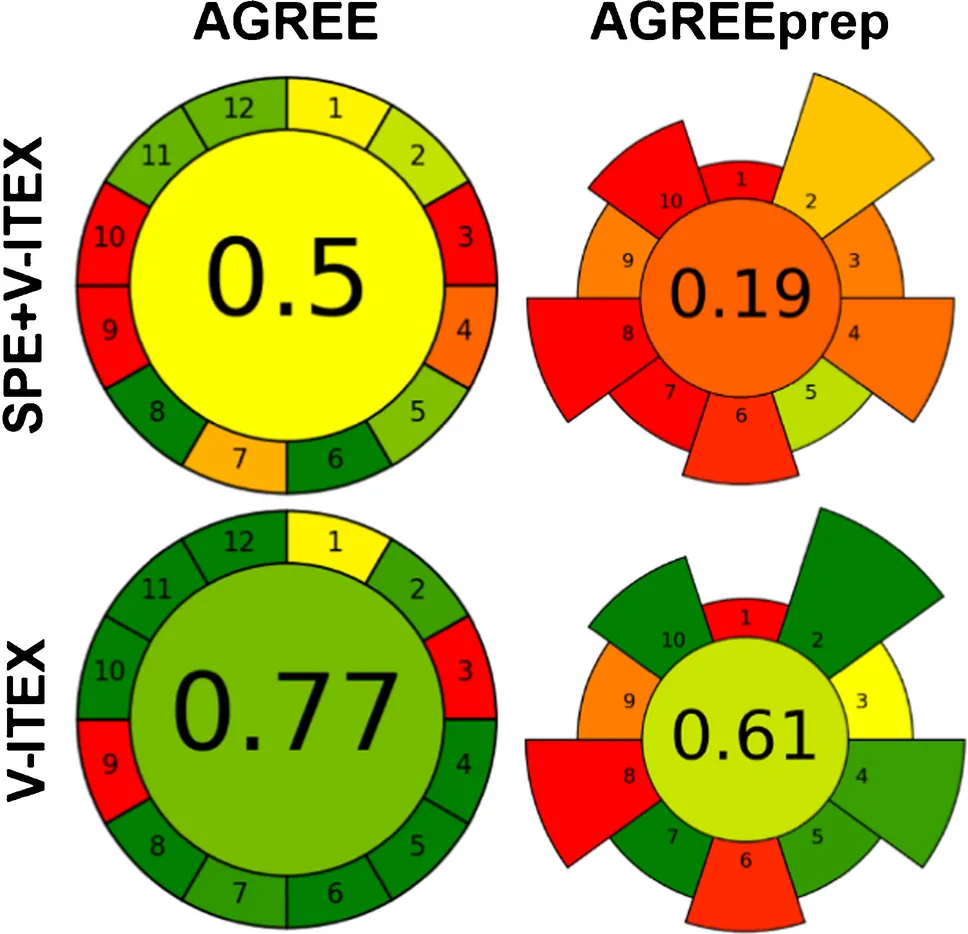
Pictograms of Analytical Greenness Metric (AGREE) and Analytical Greenness Metric for Sample Preparation (AGREEprep) for the evaluation of the greenness of the SPE+V-ITEX and V-ITEX procedures

We evaluated the greenness of the methods using AGREE and AGREEprep using the assumptions displayed in Table S3 in ESM. The solvent-free V-ITEX direct measurement of urine clearly emerges as a more environmentally friendly approach (see Fig. 5). Both assessment tools are based on the principles of green analytical chemistry (GAC), with the individual parameters and their rationale discussed in detail by Peña-Pereira et al. [18] and Wojnowski et al. [19]. They share certain weaknesses that are penalized in the AGREE and AGREEprep evaluations. For instance, ex-situ sampling and off-line analysis are rated poorly (parameters 1 and 3 in both tools), as GAC favors direct, on-line methods with minimal sample preparation. Additionally, the high energy consumption of GC-MS leads to further negative ratings (parameter 9 in AGREE; parameters 8 and 9 in AGREEprep). The low sample throughput of 1.43 samples per hour also results in a poor rating, which is AGREEprep parameter 6. Although AGREEprep does not assess the number of analytes detected, this factor favorably influences the AGREE score of parameter 8, compensating for the throughput limitation.

V-ITEX demonstrates clear environmental and operational advantages over SPE+V-ITEX and should be considered a greener and more sustainable extraction technique for urinary metabolomics. As a fully automated, solvent-free, and direct microextraction technique, it aligns closely with GAC principles. By contrast, the SPE+V-ITEX procedure received only a moderate rating in AGREE and a poor rating in AGREEprep. The main drawbacks include the use of toxic solvents and acid, higher waste generation, and the necessity of numerous manual steps. This evaluation supports method selection based not only on analytical performance but also on environmental and practical sustainability.

In addition to environmental aspects, analytical performance and practical applicability play a decisive role. In the present study, both extraction workflows showed comparable metabolite coverage and repeatability, while SPE+V-ITEX provided higher signal intensities for selected compound classes. In contrast, direct V-ITEX offered clear advantages in terms of automation, reduced manual handling, and workflow simplicity, resulting in higher practical applicability for routine and high-throughput analyses. Taken together, these aspects complement the greenness assessment and support direct V-ITEX as a balanced compromise between performance, practicality, and sustainability.

## Conclusion

This study presents a systematic comparison and optimization of SPE+V-ITEX and V-ITEX for the extraction of volatile urinary metabolites. While SPE+V-ITEX offers high total signal intensity, V-ITEX provides distinct advantages in compound-class-specific sensitivity, automation, and environmental sustainability. The results underline the importance of method selection based on the target analyte class and the intended application. V-ITEX emerges as a promising, greener alternative for large-scale nutritional metabolomics, with performance parameters such as trap material, extraction time, and desorption flow requiring tailored adjustment. This study deepens our understanding of how extraction parameters influence metabolite coverage and supports the refinement of analytical strategies in nutritional metabolomics.

## Supplementary Information

Below is the link to the electronic supplementary material.

Supplementary file1 (PDF 7.37 MB)

## Data Availability

Data will be made available upon reasonable request.

## Declarations

### Source of biological material

The urine samples used in the present methodological evaluation were obtained from four healthy male participants enrolled in the FermentoMilk study. Samples were anonymized prior to analysis and handled in accordance with applicable ethical and data protection regulations.

### Declaration of AI use

The authors acknowledge the use of ChatGPT (OpenAI, ChatGPT 4o, and ChatGPT 5, last accessed November 2025) to assist in the refinement of English grammar, language editing, and stylistic improvements. Certain graphical elements in the graphical abstract were generated using ChatGPT. These elements are original, do not reproduce any third-party copyrighted material, and are released into the public domain. They have been adapted and edited for the purposes of this work, and their use is unrestricted for both scientific and commercial publications.
