# Supplementary material for: Evaluation and optimization of SPE pre-processing combined with V-ITEX-GC-MS versus direct V-ITEX-GC-MS for urinary volatilome profiling
Source: Anal Bioanal Chem. 2026 Mar 31;418(12):3645–63. doi: 10.1007/s00216-026-06469-3 (PMC13652928; doi:10.1007/s00216-026-06469-3)
Supplement: Supplementary file 1 [file 216_2026_6469_MOESM1_ESM.pdf]

Supporting Information to: Evaluation and optimization of SPE pre-processing combined with V-ITEX-GC-MS versus direct V-ITEX-GC-MS for urinary volatilome profiling

Lucie K. Tintrop[1], Doris Zbinden[1], Marie-Claire Risse[1], Pascal Fuchsmann[1]

[1]Agroscope, Schwarzenburgstrasse 161, 3003 Bern, Switzerland

Optimized workflows of the procedures

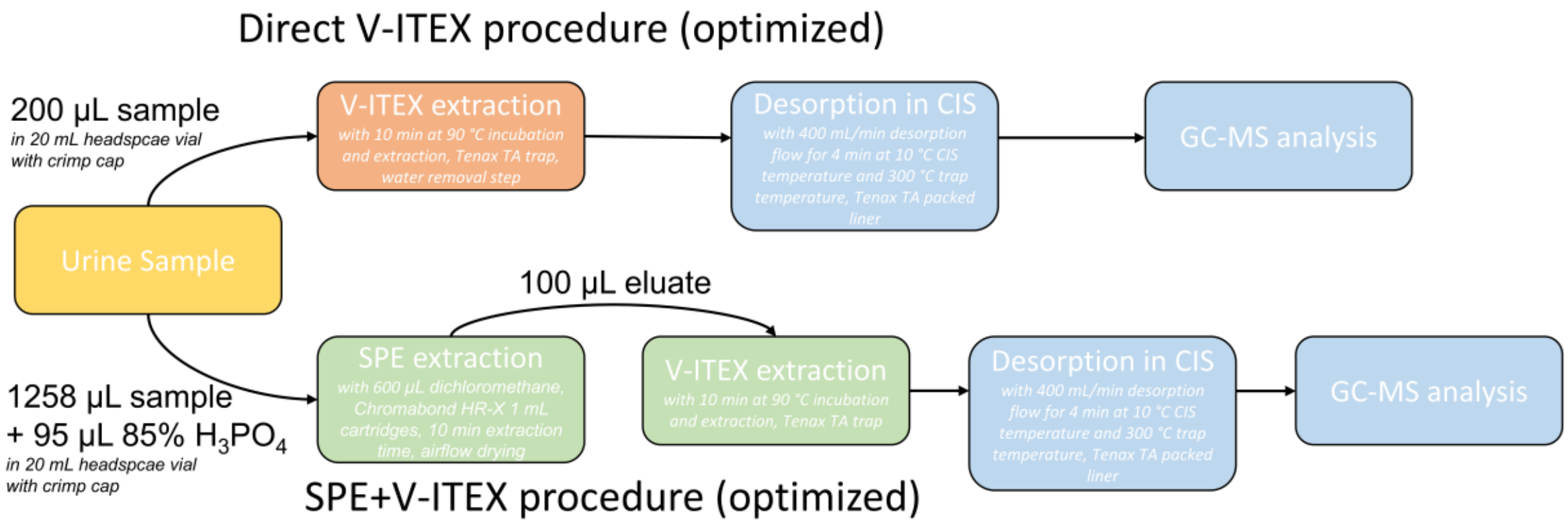

Figure S1 Direct comparison of the two workflows with optimized parameters.

DoE tables

Table S1 DoE table for the optimization of vacuum in-tube extraction with parameter combinations of extraction time, desorption flow, ITEX trap material and extraction temperature.

No.	Extraction time [min]	Desorption flow [mL/min]	ITEX trap material	Extraction temperature [°C]
**1**	10	20	Tenax TA/Carbosieve SIII	40
**2**	10	400	Tenax TA/Carbosieve SIII	40
**3**	0.5	20	Tenax TA/Carbosieve SIII	90
**4**	0.5	400	Tenax TA/Carbosieve SIII	90
**5**	0.5	210	Tenax TA/Carbosieve SIII	40
**6**	10	210	Tenax TA/Carbosieve SIII	90
**7**	0.5	20	Tenax TA	40
**8**	10	400	Tenax TA	40
**9**	0.5	20	Tenax TA	90
**10**	10	20	Tenax TA	90
**11**	0.5	400	Tenax TA	90
**12**	10	20	Tenax TA/Carboxen	40
**13**	0.5	400	Tenax TA/Carboxen	40
**14**	0.5	20	Tenax TA/Carboxen	90
**15**	10	400	Tenax TA/Carboxen	90
**16**	5.25	210	Tenax TA/Carboxen	65
**17**	0.5	20	Carbosieve SIII	40
**18**	10	400	Carbosieve SIII	40
**19**	10	20	Carbosieve SIII	90
**20**	0.5	400	Carbosieve SIII	90
**21**	0.5	20	Carboxen	40
**22**	0.5	400	Carboxen	40

**23**	10	20	Carboxen	90
**24**	10	400	Carboxen	90
**25**	0.5	210	Carboxen	90
**26**	10	210	Carboxen	40
**27**	5.25	210	Carboxen	65
**28**	5.25	210	Carboxen	65
**29**	5.25	210	Carboxen	65

Table S2 DoE table for the optimization of solid-phase extraction with parameter combinations of drying strategy, extraction time, elution solvent, and acid concentration of the sample.

**No.**	**Drying strategy**	**Extraction time [min]**	**Solvent**	**Acid concentration [%]**
**1**	20 min N_2	10	Acetonitril	0
**2**	20 min N_2	10	Acetonitril	6
**3**	20 min N_2	0.5	Acetonitril	3
**4**	20 min air-flow with vacuum	0.5	Acetonitril	0
**5**	20 min air-flow with vacuum	10	Acetonitril	0
**6**	20 min air-flow with vacuum	0.5	Acetonitril	6
**7**	20 min air-flow with vacuum	10	Acetonitril	6
**8**	20 min N_2	0.5	Acetone	0
**9**	20 min N_2	10	Acetone	0
**10**	20 min N_2	0.5	Acetone	6
**11**	20 min N_2	10	Acetone	6
**12**	20 min air-flow with vacuum	10	Acetone	0
**13**	20 min air-flow with vacuum	10	Acetone	6
**14**	20 min air-flow with vacuum	0.5	Acetone	3
**15**	20 min N_2	0.5	Ethylacetat	0
**16**	20 min N_2	10	Ethylacetat	6
**17**	20 min air-flow with vacuum	10	Ethylacetat	0
**18**	20 min air-flow with vacuum	0.5	Ethylacetat	6
**19**	20 min N_2	10	Dichloromethane	0
**20**	20 min N_2	0.5	Dichloromethane	6
**21**	20 min air-flow with vacuum	0.5	Dichloromethane	0
**22**	20 min air-flow with vacuum	10	Dichloromethane	6
**23**	20 min air-flow with vacuum	5.25	Dichloromethane	3
**24**	20 min air-flow with vacuum	5.25	Dichloromethane	3
**25**	20 min air-flow with vacuum	5.25	Dichloromethane	3

SPE N_2 drying strategy

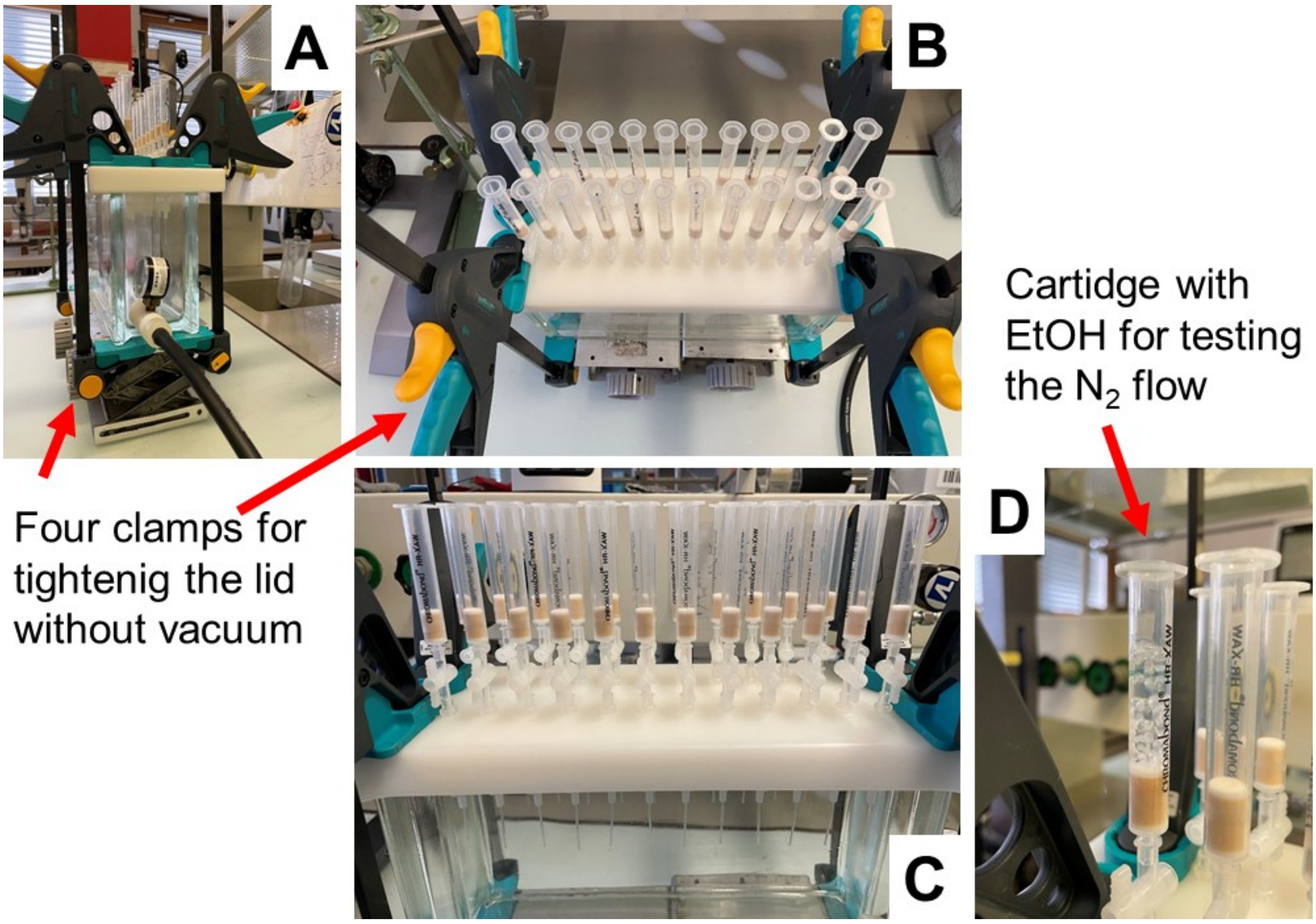

Figure S2 SPE drying setup using N_2. The SPE cartridges (exemplarily shown for HR-XAW cartridges) are fixed on the vacuum manifold holder. The vacuum manifold was connected to a N_2 pipe and the lid was tightened with 4 clamps (one clamp at each corner). One empty cartridge was filled with Ethanol and used for testing the N_2 flow (if there are bubbles, the N_2 was flowing).

Parameters for greenness evaluation metrics

Table S3 List of the parameters and settings in Analytical Greenness metric (AGREE) and Analytical greenness metric for sample preparation (AGREEprep). Specific comments are written in italics.

	**V-ITEX**	**SPE**
**Parameter (weight)**	**Setting**	**Setting**
1. Sampling procedure (2)	off-line analysis	off-line analysis
2. Amount of sample (2)	0.2 mL	1.258 mL
3. Position of analytical device (2)	off-line	off-line
4. Number of steps in sample preparation (2)	3 or fewer<br>(*Pipetting, heating, Extraction*)	7 steps<br>*(SPE Conditioning, Sample pipetting, Acidification, SPE loading, SPE drying, SPE Elution, Elutate pipetting)*
5. Automation and miniaturization (2)	Degree of automation: automatic; Sample preparation: none or miniaturized	semi-automatic, none or miniaturized
6. Derivatization agent (2)	None	None
7. Waste amount (2)	0.2 mL<br>(*sample volume*)	13.353 mL<br>*(Sample+acid = 1353 uL; SPE conditioning = 12 mL; Total = 13.353 mL)*

8. Number of analytes in one run and sample throughput per hour (2)	145 analytes determined in one run; 1.43 samples per hour	145 analytes determined in one run; 1.43 samples per hour
9. Most energy-intensive technique (2)	GC-MS	GC-MS
10. Type of reagents (2)	No reagents	None of the reagents are from biobased sources
11. Use of toxic reagents of solvents (2)	None	Yes; amout: 0.6 mL<br>*(Dichloromethane)*
12. Operators safety; Threats that are not avoided (2)	None	Corrosive<br>*(Phosphoric acid, not possible to state health hazards such as carcinogenic here)*
**AGREEprep**		
	**V-ITEX**	**SPE**
**Parameter (weight)**	**Setting**	**Setting**
1. Sample preparation placement (1)	Ex-situ	Ex-situ
2. Amount of hazardous materials (5)	None	0.695 mL<br>*(DCM 600 µL, 95 µL Acid = 695 µL)*
3. Sustainability, renewability, and reusability of materials (2)	Materials are not sustainable or renewable but are used several times	25-50% of the reagents and materials are renewable<br>*(ITEX, trap, SPE cartridges, partly solvents)*
4. Waste amount (4)	0.2 mL	13.353<br>*(Sample+acid = 1353 µL; SPE conditioning = 12 mL; Total = 13.353 mL)*
5. Sample volume (2)	0.2 mL	1.258 mL
6. Sample throughput (3)	1.43 samples per hour	1.43 samples per hour
7. Integration and automation (2)	2 steps or fewer; fully automated system	6 steps or more; semi-automated system<br>*(SPE conditioning, sample pipetting, acidification, SPE loading, SPE drying, SPE elution, elutate pipetting)*
8. Energy consumption (4)	664 wh/sample<br>*(Estimated: GC oven 250 Wh/h; Injector: 150 W/h; Detector: 150 Wh/h; Vacuum pump: 300 Wh/h; PC: 100 Wh/h = 950 wh/h:1.43 = 664 wh/sample)*	699.3 wh/sample<br>*(Estimated: GC oven 250 Wh/h; Injector: 150 W/h; Detector: 150 Wh/h; Vacuum pump: 300 Wh/h; PC: 100 Wh/h; Vacuum pump SPE Elution; 50 kwh/h = 1000 wh/h:1.43 = 699.3 wh/sample)*
9. Post sample preparation configuration for analysis (2)	LC, GC with quadrupole detection	LC, GC with quadrupole detection
10. Operators safety (3)	No hazards or no exposure	4 or more hazards<br>*(Solvents for conditioning, elution solvent and acid)*

Biological source of identified compounds

Table S4 Putatively annotated compounds and their potential source according to the Human Metabolome Database (HMDB) sorted by their chemical groups. Identification numbers of the HMBD, when available, (HMDB ID) are given in brackets. NA: Data not available.

Group	Compound (HMDB ID)	Source (1)
Alcohols	1-Hexanol, 2-ethyl- (HMDB0031231)	-Endogenous<br>-Exogenous (cereal, tea, fruit, alcoholic beverage, fat and oil)
	Benzyl alcohol (HMDB0003119)	-Exogenous (herb, spice, crop, seed, nut, fruit, vegetable, cereal, pulse, tea, coffee)
	1-Octanol (HMDB0001183)	-Biological location (tissue, cellular substructure, cell, tissue substructure, organ, excreta)<br>-Exogenous (animal origin, fermented beverage, vegetable, pulse, soy, herb and spice, cereal and cereal product, gourd, fruit, tea)
	2-Phenyl-2-propanol	NA
	Ethanol, 2-phenoxy- (HMDB0041607)	-Biological location (non-excretory biofluid, excreta, cellular substructure)<br>-Exogenous (food, personal care products)
Aldehydes	Hexanal (HMDB0005994)	-Biological location (non-excretory biofluid, excreta, cellular substructure,<br>-Endogenous (plant, animal)<br>-Exogenous (animal origin, herb and spice, vegetable, fruit, nut, cereal and cereal product, pulse, gourd, coffee and coffee product, soy, dish, tea, baking good, beverage, aquatic origin, milk and milk product, snack, cocoa and cocoa product)
	Furfural (HMDB0032914)	NA
	Heptanal (HMDB0031475)	-Biological location (non-excretory biofluid, cellular substructure, excreta)<br>-Endogenous (plant, animal)<br>-Exogenous (animal origin, herb and spice, nut, fruit, vegetable, cereal and cereal product, pulse, soy, aquatic origin, gourd, milk and milk product, tea, snack, dish, cocoa and cocoa product)
	Benzaldehyde (HMDB0006115)	-Biological location (non-excretory biofluid, excreta)<br>-Endogenous (plant, animal)<br>-Exogenous (animal origin, herb and spice, vegetable, fruit, nut, cereal and cereal product, pulse, gourd, coffee and coffee product, soy, dish, tea, baking good, beverage, aquatic origin, snack, cocoa and cocoa product, milk and milk product)
	Octanal (HMDB0001140)	-Biological location (cellular substructure, non-excretory biofluid, excreta)<br>-Endogenous (plant, animal)<br>-Exogenous (animal origin, beverage, herb and spice, nut, fruit, vegetable, cereal and cereal product, pulse, soy, aquatic origin, gourd, tea, snack, cocoa and cocoa product)
	Benzeneacetaldehyde (HMDB0006236)	-Biological location (excreta, cellular substructure)<br>-Exogenous (animal origin, herb and spice, vegetable, fruit, nut, cereal and cereal product, pulse, gourd, soy, dish, tea, baking good, fermented beverage, aquatic origin, milk and milk product, snack, cocoa et cocoa product, environmental)<br>-Endogenous (plant, animal)
	Nonanal (HMDB0059835)	-Biological location (non-excretory biofluid, cellular substructure, excreta)<br>-Exogenous (fermented beverage, fruit, fereal and cereal product, vegetable, herb and spice, gourd, milk and milk product, tea, dish, pulse)
	Benzaldehyde, 2-ethyl-	NA
	2-Nonenal, (E)- (HMDB0255708)	-Biological location (non-excretory biofluid)<br>-Exogenous
	Decanal (HMDB0011623)	-Biological location (non-excretory biofluid, cellular substructure, excreta)<br>-Endogenous (plant, animal)<br>-Exogenous (animal origin, beverage, vegetable, herb and spice, gourd, fruit, tea, cereal and cereal product, pulse, nut, aquatic origin, fat and oil
	Benzaldehyde, 2,4-dimethyl- (HMDB0032142)	-Biological location (cellular substructure)<br>-Endogenous<br>-Exogenous (food)

	Undecanal (HMDB0030941)	-Biological location (cellular substructure, non-excretory biofluid, excreta)<br>-Exogenous (food, vegetable, herb and spice, cereal, gourd, fruit)<br>-Endogenous (plant)
	Dodecanal (HMDB0033933)	-Biological location (cellular substructure, non-excretory biofluid, excreta)<br>-Endogenous (plant, animal)<br>-Exogenous (fermented beverage, vegetable, herb and spice, gourd, fruit, aquatic origin)
	Tetradecanal (HMDB0034283)	-Biological location (cellular substructure, non-excretory biofluid, excreta)<br>-Endogenous (plant, animal)<br>-Exogenous (vegetable, herb and spice, gourd, fruit)
	3,5-di-tert-Butyl-4-hydroxybenzaldehyde (HMDB0240643)	-Biological location (excreta)<br>-Exogenous
Aliphatic hydrocarbons	1-Octene (HMDB0032449)	-Biological location (cellular substructure)<br>-Endogenous<br>-Exogenous (cereal and cereal product, milk and milk product)
	3-Heptene, 2,2,4,6,6-pentamethyl- (HMDB0094665)	-Biological location (excreta)<br>-Exogenous
	Nonane, 2,5-dimethyl-	NA
	Octane, 2,3,6,7-tetramethyl-	NA
	Undecane (HMDB0031445)	-Biological location (cellular substructure, non-excretory biofluid, biofluid or excreta)<br>-Endogenous (plant)<br>-Exogenous (Cardamon)
	Undecane, 3-methyl-	NA
	Dodecane (HMDB0031444)	-Biological location (cellular substructure, non-excretory biofluid, biofluid or excreta)<br>-Endogenous (plant)<br>-Exogenous (pulse, soy, vegetable, fruit, nut)
	Dodecane, 2-methyl- (HMDB0031287)	-Biological location (cellular substructure)<br>-Endogenous (plant)<br>-Exogenous (pulse, beverage)
	Dodecane, 3-methyl-	NA
	Tridecane (HMDB0034284)	-Biological location (cellular substructure, excreta)<br>-Exogenous (vegetable, fruit, herb and spice, nut)<br>-Endogenous (plant)
	Dodecane, 4,6-dimethyl-	NA
	Tridecane, 3-methyl-	NA
	Tetradecane (HMDB0059907)	-Biological location (cellular substructure, non-excretory biofluid, biofluid or excreta)<br>-Exogenous (cereal and cereal product, gourd, herb and spice, fruit, vegetable, nut)
	Cyclopentane, nonyl-	NA
	5,5-Diethyltridecane	NA
	Heptadecane (HMDB0059830)	-Biological location (cellular substructure, non-excretory biofluid, excreta)<br>-Exogenous
Aromatic hydrocarbons	Toluene (HMDB0034168)	-Exogenous (fruit)
	Ethylbenzene (HMDB0059905)	-Biological location (cellular substructure, non-excretory biofluid, excreta)<br>-Exogenous (environmental, vegetable, gourd, herb and spice, nut, pulse)
	Benzene, 1,3-dimethyl-	NA
	Styrene (HMDB0034240)	-Biological location (cellular substructure, non-excretory biofluid, excreta)<br>-Exogenous (fruit, tea, vegetable, herb and spice, coffee and coffee product, cocoa and cocoa product, nut, alcoholic beverage, milk and milk product, environmental)<br>-Endogenous (plant, animal)
	Benzene, 1-methyl-3-(1-methylethenyl)- (HMDB0037051)	-Biological location (cellular substructure, excreta)<br>-Exogenous (fruit, herb and spice)<br>-Endogenous (plant)
	Benzene, 1,2,4,5-tetramethyl-	NA
	Benzene, pentyl-	NA

	Naphthalene (HMDB0029751)	-Biological location (cellular substructure, non-excretory biofluid, excreta)<br>-Endogenous (plant)<br>-Exogenous (plant, vegetable, cereal and cereal product, herb and spice, nut, synthetic, environmental)
	Benzene, 1,2,4-triethyl-	NA
	1H-Indene, 2,3-dihydro-1,1,3-trimethyl-	NA
	Benzene, 1,3-bis(1,1-dimethylethyl)-	NA
	Naphthalene, 2-methyl-	NA
	Naphthalene, 1,4-dimethyl-	NA
	Benzene, 1-(1,5-dimethyl-4-hexenyl)-4-methyl-	NA
	1H-Indene, 2,3-dihydro-1,1,3-trimethyl-3-phenyl-	NA
	2,6-Diisopropylnaphthalene (HMDB0059902)	-Biological location (cellular substructure)<br>-Exogenous
	Benzene, (1-butyloctyl)-	NA
	Benzene, (1-pentyloctyl)-	NA
Azine	1,3,5-Triazine-2,4,6(1H,3H,5H)-trione, 1,3,5-tri-2-propen-1-yl-	NA
Cyclic hydrocarbons	2-Bornene	NA
	3-Thujene (HMDB0036116)	-Biological location (cellular substructure)<br>-Endogenous (plant, animal)<br>-Exogenous (vegetable, herb and spice, fruit)
Esters	3-Methylcyclopentyl acetate	NA
	n-Butyl methacrylate (HMDB0249475)	-Biological location (non-excretory biofluid)<br>-Exogenous
	2-Propenoic acid, 2-methyl-, 2-hydroxypropyl ester	NA
	Benzoic acid, ethyl ester (HMDB0033967)	NA
	Methyl salicylate (HMDB0034172)	-Biological location (cellular substructure, excreta)<br>-Exogenous (fruit, cereal and cereal product, vegetable, tea, herb and spice, beverage, synthetic)<br>-Endogenous (plant)
	Methacrylic acid, tetrahydrofurfuryl ester	NA
	Isopentyl methacrylate	NA
	3-hydroxy-2,2,4-trimethylpentyl isobutyrate	NA
	exo-1,7,7-trimethylbicyclo[2.2.1]hept-2-yl isobutyrate	NA
	2,2,4-Trimethyl-1,3-pentanediol diisobutyrate (HMDB0059777)	-Biological location (cellular substructure, excreta)<br>-Exogenous
	2-Ethylhexyl salicylate	NA
	Isopropyl myristate	NA
	1,2-Benzenedicarboxylic acid, bis(2-methylpropyl) ester	NA
	Isopropyl palmitate (HMDB0035474)	-Biological location (cellular substructure, non-excretory biofluid, excreta)<br>-Endogenous (animal)<br>-Exogenous (food)
Hetero aromats	Pyrrole (HMDB0035924)	-Biological location (non-excretory biofluid, cellular substructure)<br>-Exogenous (beverage, pulse, soy, cereal and cereal product, fruit, snack, dish)<br>-Endogenous (plant)
	Benzofuran (HMDB0032929)	-Biological location (cellular substructure)<br>-Exogenous (tea, coffee and coffee product, herb and spice, vegetable, beverage)<br>-Endogenous (plant)
	Benzofuran, 2,3-dihydro- (HMDB0013815)	-Biological location (excreta)<br>-Endogenous (animal origin)
	Coumarin (HMDB0001218)	-Biological location (cellular substructure, non-excretory biofluid)<br>-Exogenous (herb and spice, vegetable, fruit, nut, cereal and cereal product, pulse, gourd, coffee and coffee product, soy, tea, dish, baking good, aquatic origin, cocoa and cocoa product)

Ketones	3-Penten-2-one (HMDB0001184)	-Biological location (excreta, non-excretory biofluid)<br>-Endogenous<br>-Exogenous (animal origin)
	3-Hexanone (HMDB0000753)	-Biological location (excreta)<br>-Endogenous<br>Exogenous (animal origin, nut, herb and spice, vegetable, fruit, cereal and cereal product, pulse, gourd, coffee and coffee product, soy, dish, tea, baking good, aquatic origin, cocoa
	3-Hexanone, 2-methyl- (HMDB0061896)	-Biological location (non-excretory biofluid, excreta)<br>-Exogenous
	Cyclopentanone, 3-methyl-	NA
	4-Heptanone (HMDB0004814)	-Biological location (cellular substructure, non-excretory biofluid, excreta)<br>-Endogenous<br>-Exogenous (animal origin)
	4-Cyclopentene-1,3-dione	NA
	2-Heptanone (HMDB0003671)	-Biological location (cellular substructure, non-excretory biofluid, excreta)<br>-Endogenous (plant, animal)<br>-Exogenous (animal origin, beverage, vegetable, cereal and cereal product, herb and spice, milk and milk product, fruit, tea, gourd, snack, dish, pulse, aquatic origin)
	2-Heptanone, 4-methyl-	NA
	3-Ethylcyclopentanone	NA
	2,3-Octanedione (HMDB0031293)	-Biological location (non-excretory biofluid, cellular substructure)<br>-Endogenous (plant)<br>-Exogenous (herb and spice, coffee and coffee product, fruit)
	Acetophenone (HMDB0033910)	NA
	4-Acetyl-1-methylcyclohexene (HMDB0033434)	-Biological location (cellular substructure)<br>-Exogenous (cereal and cereal product)<br>-Endogenous (plant)
	2,6,6-Trimethyl-2-cyclohexene-1,4-dione (HMDB0040216)	-Biological location (cellular substructure)<br>-Endogenous (plant)<br>-Exogenous (tea)
	Ethanone, 1-(4-methylphenyl)- (HMDB0032608)	-Biological location (cellular substructure)<br>Endogenous (plant, animal)<br>-Exogenous (vegetable, herb and spice, fruit, beverage, milk and milk product)
	2-Decanone (HMDB0031409)	-Biological location (cellular substructure, non-excretory biofluid, excreta)<br>-Endogenous (plant)<br>-Exogenous (vegetable)
	2-Methoxyphenylacetone	NA
	2-Propanone, 1-(4-methoxyphenyl)- (HMDB0032891)	-Biological location (cellular substructure)<br>-Exogenous (herb and spice)<br>-Endogenous (plant)
	5,9-Undecadien-2-one, 6,10-dimethyl- (HMDB0031846)	-Biological location (cellular substructure)<br>-Exogenous (fruit, vegetable, cereal and cereal product, gourd, herb and spice, snack)<br>-Endogenous
	Benzophenone (HMDB0032049)	-Biological location (cellular substructure, excreta, non-excretory biofluid)<br>-Exogenous (fruit, synthetic)<br>-Endogenous (plant)
	7-Pentadecanone (HMDB0302200)	-Exogenous (herb and spice)
	Ethanone, 2,2-dimethoxy-1,2-diphenyl-	NA
Lactam	Caprolactam (HMDB0062769)	-Biological location (non-excretory biofluid)<br>-Exogenous (herb and spice)
Lactones	γ-Crotonolactone (HMDB0032330)	-Biological location (cellular substructure)<br>-Endogenous<br>-Exogenous

	γ-Butyrolactone (HMDB0000549)	-Biological location (tissue, non-excretory biofluid, cell, excreta, cellular substructure)<br>-Exogenous (animal origin, beverage, pulse, soy, fruit, vegetable, tea, snack)<br>-Endogenous
	γ-Pentalactone	NA
	4-Methyl-4-vinylbutyrolactone (HMDB0032333)	-Biological location (cellular substructure)<br>-Exogenous<br>-Endogenous
	γ-Hexalactone (HMDB0003843)	-Biological location (excreta)<br>-Endogenous<br>-Exogenous (animal origin, fruit, vegetable, pulse, soy)
	δ-Hexalactone (HMDB0000453)	-Biological location (excreta, cellular substructure)<br>-Endogenous<br>-Exogenous (animal origin, fruit, milk and milk product)
	2-Coumaranone	NA
	γ-Octalactone (HMDB0035422)	-Biological location (cellular substructure)<br>-Exogenous (vegetable, fruit, snack)<br>-Endogenous (plant)
	γ-Nonalactone	NA
	Dodecanolactone	NA
Nitriles	Isoamyl cyanide	NA
	Allyl Isothiocyanate (HMDB0005843)	-Biological location (non-excretory biofluid, excreta, cellular substructure)<br>-Endogenous (plant)<br>-Exogenous (animal origin, vegetable, herb and spice, baking good)
	Benzonitrile	NA
	Cyclohexane, isothiocyanato-	NA
	Dodecanenitrile	NA
Phtalates	Diethyl Phthalate (HMDB0094660)	-Biological location (excreta, cellular substructure)<br>-Exogenous
	Dibutyl phthalate (HMDB0033244)	-Biological location (non-excretory biofluid, cellular substructure)<br>-Exogenous
Substituted aromats	Benzene, chloro- (HMDB0041855)	-Biological location (cellular substructure)<br>-Exogenous
	Phenol (HMDB0000228)	-Biological location (tissue, non-excretory biofluid, cell, tissue substructure, organ, excreta, cellular substructure)<br>-Exogenous (beverage, vegetable, pulse, soy, fruit, cocoa and cocoa product, coffee and coffee product, milk and milk product, herb and spice, synthetic, environmental)<br>-Endogenous (plant, microbe)
	p-Cresol (HMDB0001858)	-Biological location (tissue, non-excretory biofluid, cellular substructure, cell, excreta)<br>-Exogenous (animal origin, cereal and cereal product, nut, milk and milk product, egg, soy, vegetable, fruit, tea, herb and spice, coffee and coffee product, aquatic origin, synthetic, environmental,<br>Endogenous (plant, animal, microbe)
	Formamide, N-phenyl-	NA
	Phenol, m-tert-butyl-	NA
	2-Methoxy-4-vinylphenol (HMDB0013744)	-Biological location (cellular substructure, excreta)<br>-Exogenous (beverage, vegetable, coffee and coffee product, cereal and cereal product, fruit, snack, dish)<br>-Endogenous (plant)
	Phenol, 5-ethenyl-2-methoxy- (HMDB0303765)	-Exogenous (fruit)
	Benzene, (2-isothiocyanatoethyl)- (HMDB0038445)	-Biological location (cellular substructure)<br>-Exogenous (vegetable, herb and spice)
	2,4-Di-tert-butylphenol (HMDB0013816)	-Biological location (excreta, cellular substructure)<br>-Endogenous<br>-Exogenous (animal origin)
	Phenol, 3,5-bis(1,1-dimethylethyl)-	NA
Sulfone	Dimethyl sulfone (HMDB0004983)	-Exogenous (herb and spice, nut, vegetable, cereal and cereal product, pulse, soy, gourd, tea, baking good, coffee and coffee product, dish)
Terpenes	Frontaline	NA
	Camphene (HMDB0059839)	-Biological location (cellular substructure, non-excretory biofluid, excreta)<br>-Exogenous (vegetable, herb and spice, fruit)<br>-Endogenous (animal)

	3-Carene (HMDB0035619)	-Exogenous (herb and spice)
	p-Cymene (HMDB0005805)	-Biological location (cellular substructure, excreta, non-excretory biofluid)<br>-Endogenous (plant)<br>-Exogenous (fruit, vegetable, herb and spice, milk and milk product, beverage, gourd)
	D-Limonene (HMDB0003375)	-Biological location (cellular substructure, non-excretory biofluid, excreta, tissue, organ)<br>-Endogenous (plant, animal)<br>-Exogenous (animal origin, herb and spice, vegetable, fruit, nut, cereal and cereal product, pulse, gourd, coffee and coffee product, soy, dish, tea, baking good)
	Eucalyptol (HMDB0004472)	-Biological location (non-excretory biofluid, tissue, excreta, cellular substructure)<br>-Exogenous (herb and spice, vegetable, fruit, nut, cereal and cereal product, pulse, gourd, coffee and coffee product, soy, tea, baking good, dish)
	Camphor (HMDB0059838)	-Biological location (cellular substructure)<br>-Exogenous (herb and spice, milk and milk product
	Terpinen-4-ol (HMDB0035833)	-Exogenous (herb and spice, fruit, beverage)
	Carvone (HMDB0035824)	NA
	Piperitone (HMDB0034975)	-Biological location (cellular substructure, excreta, non-excretory biofluid)<br>-Endogenous (plant, animal)<br>-Exogenous (herb and spice, fruit)
	Longifolene (HMDB0302687)	-Exogenous (milk and milk product, cereal and cereal product, fruit, herb and spice)
	γ-Muurolene (HMDB0038206)	-Biological location (cellular substructure, non-excretory biofluid)<br>-Endogenous (plant, animal)<br>-Exogenous (vegetable, milk and milk product, herb and spice, fruit, cereal and cereal product)
	10,11-Epoxycalamenene	NA
	δ-Cadinene (HMDB0035084)	-Biological location (non-excretory biofluid, cellular substructure) -Exogenous (herb and spice, nut, fruit, vegetable, cereal and cereal product, pulse, soy, gourd, tea, cocoa and cocoa product
	trans-Calamenene	NA
	Cadina-1(10),6,8-triene	NA
	α-Calacorene (HMDB0036453)	-Biological location (cellular substructure, non-excretory biofluid, excreta)<br>-Endogenous (animal, plant)<br>-Exogenous (herb and spice, vegetable, beverage)
	Cadalene (HMDB0059698)	-Biological location (cellular substructure, excreta, non-excretory biofluid)<br>-Endogenous (animal)<br>-Exogenous (fruit, herb and spice)

SPE procedure optimization contour plots for compound groups

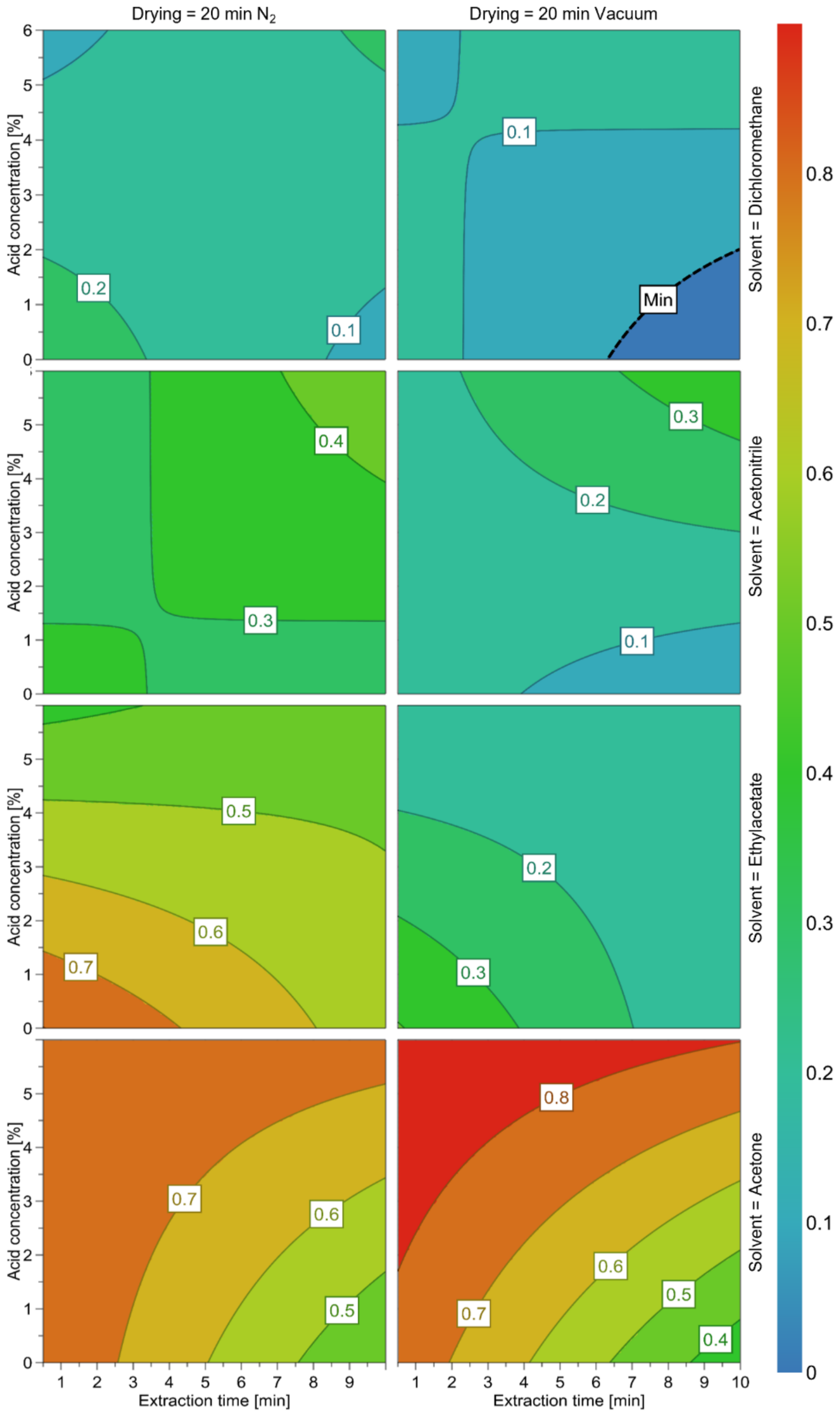

Figure S3 Contour plots for **alcohols** for the optimization of the solid-phase extraction parameters acid concentration, extraction time, solvent and drying strategy.

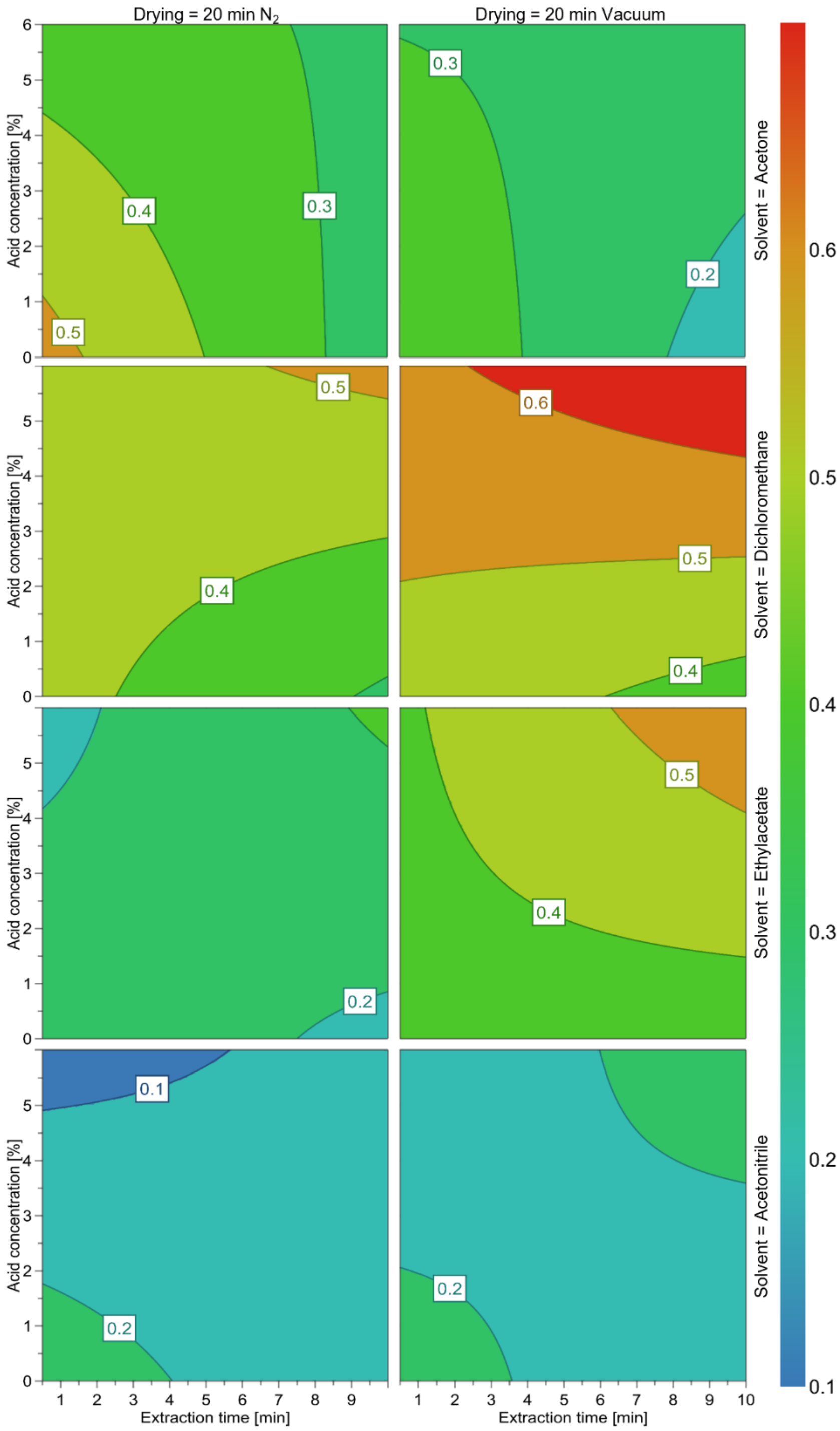

Figure S4 Contour plots for **aldehydes** for the optimization of the solid-phase extraction parameters acid concentration, extraction time, solvent and drying strategy.

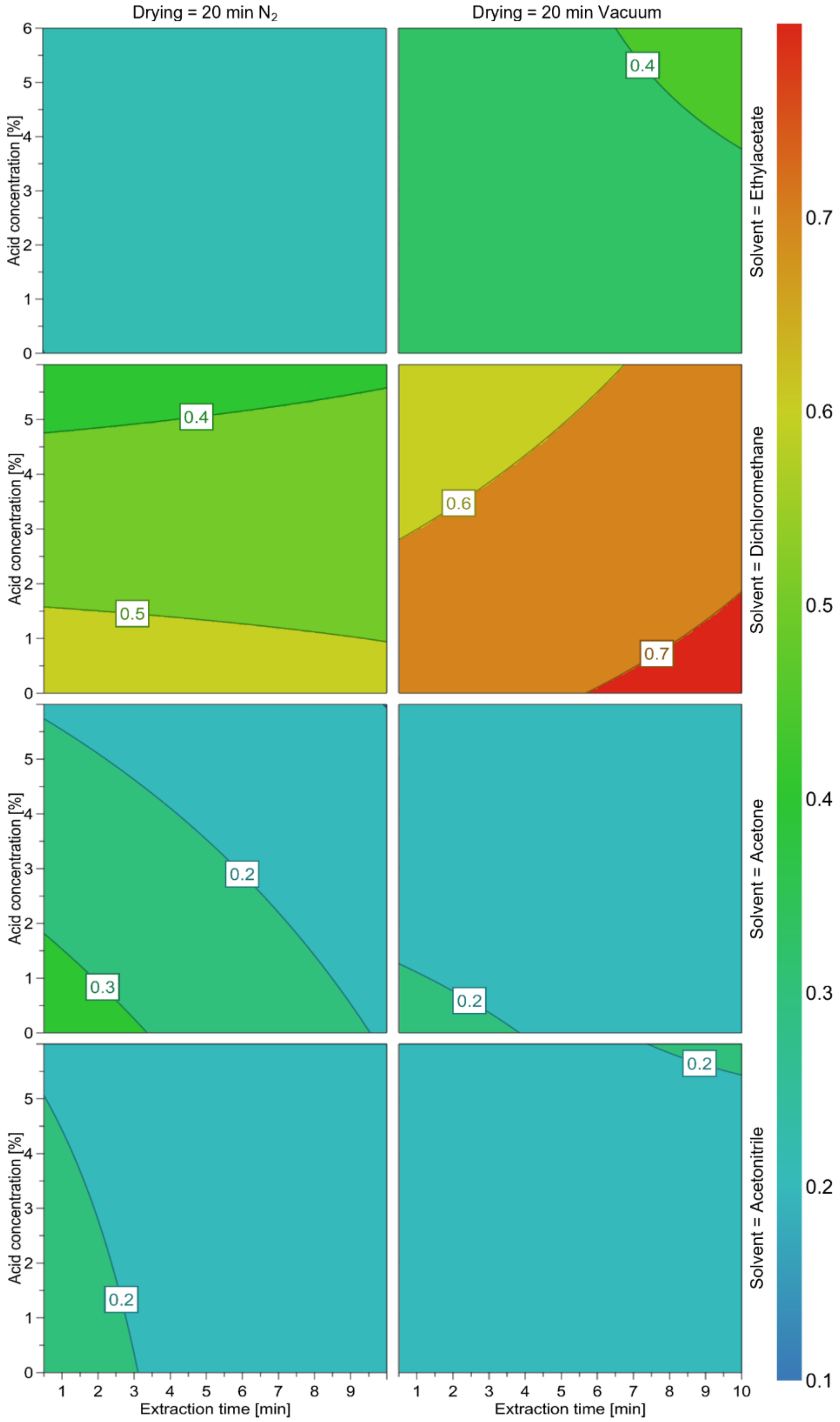

Figure S5 Contour plots for **aliphatic hydrocarbons** for the optimization of the solid-phase extraction parameters acid concentration, extraction time, solvent and drying strategy.

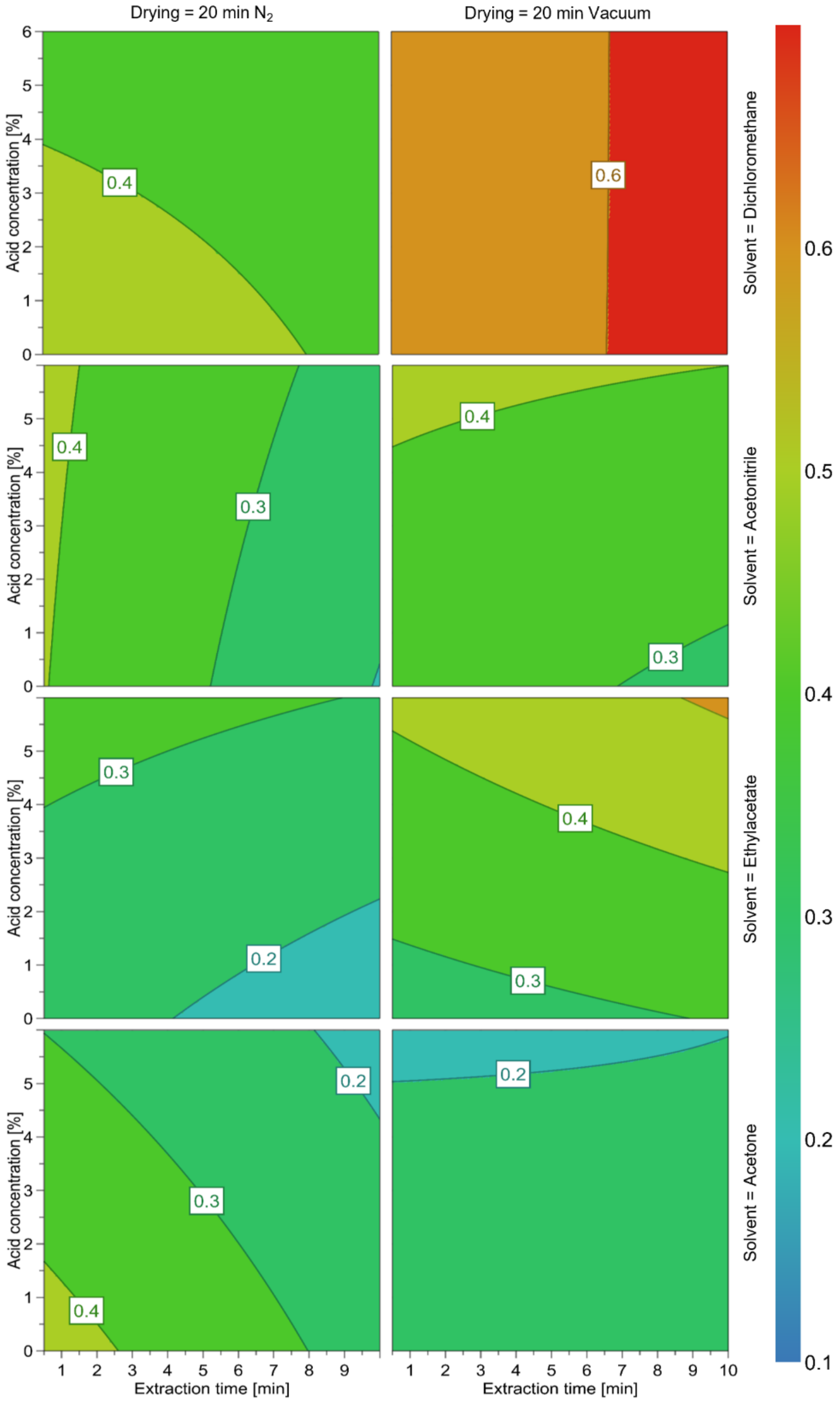

Figure S6 Contour plots for **aromatic hydrocarbons** for the optimization of the solid-phase extraction parameters acid concentration, extraction time, solvent and drying strategy.

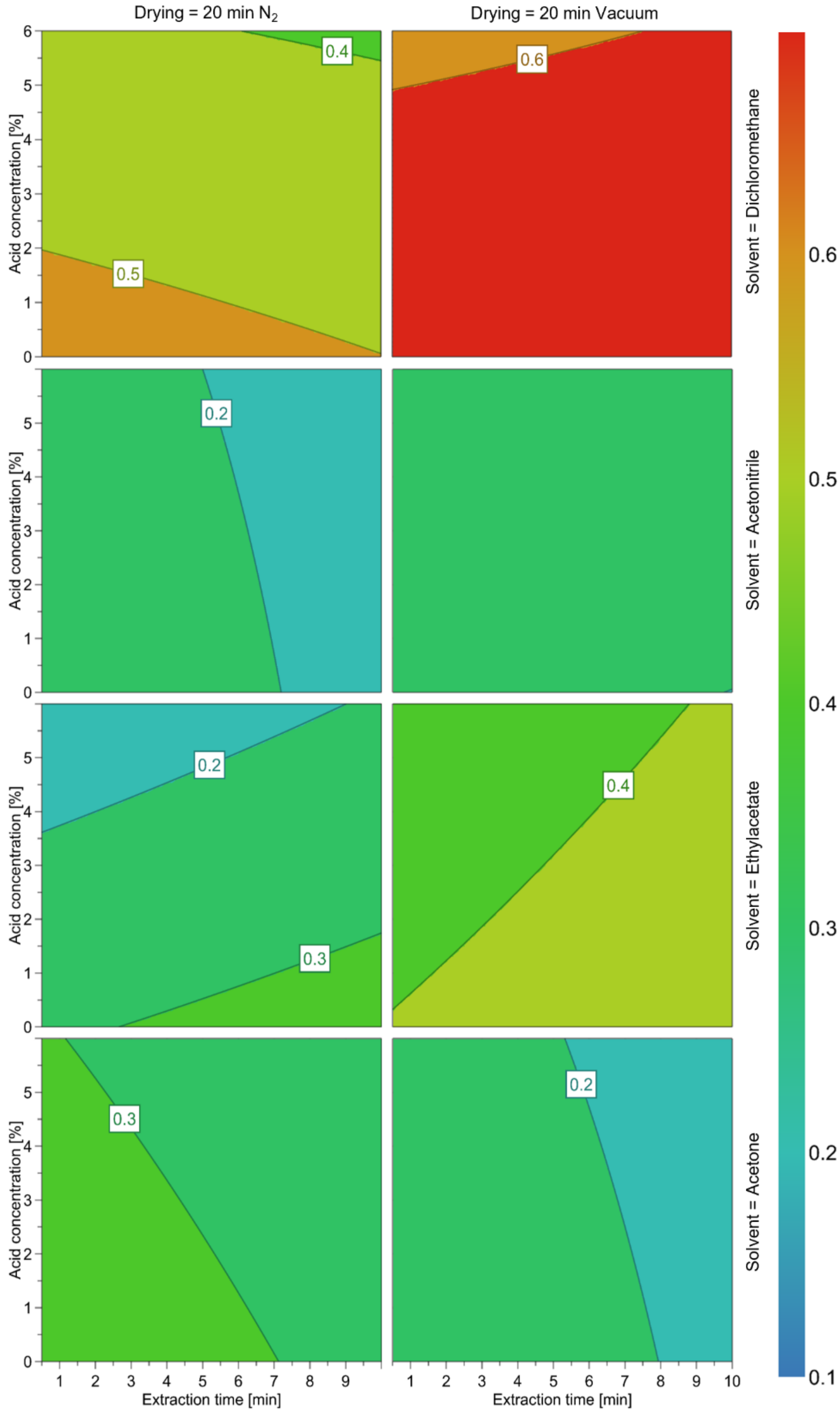

Figure S7 Contour plots for **azines** for the optimization of the solid-phase extraction parameters acid concentration, extraction time, solvent and drying strategy.

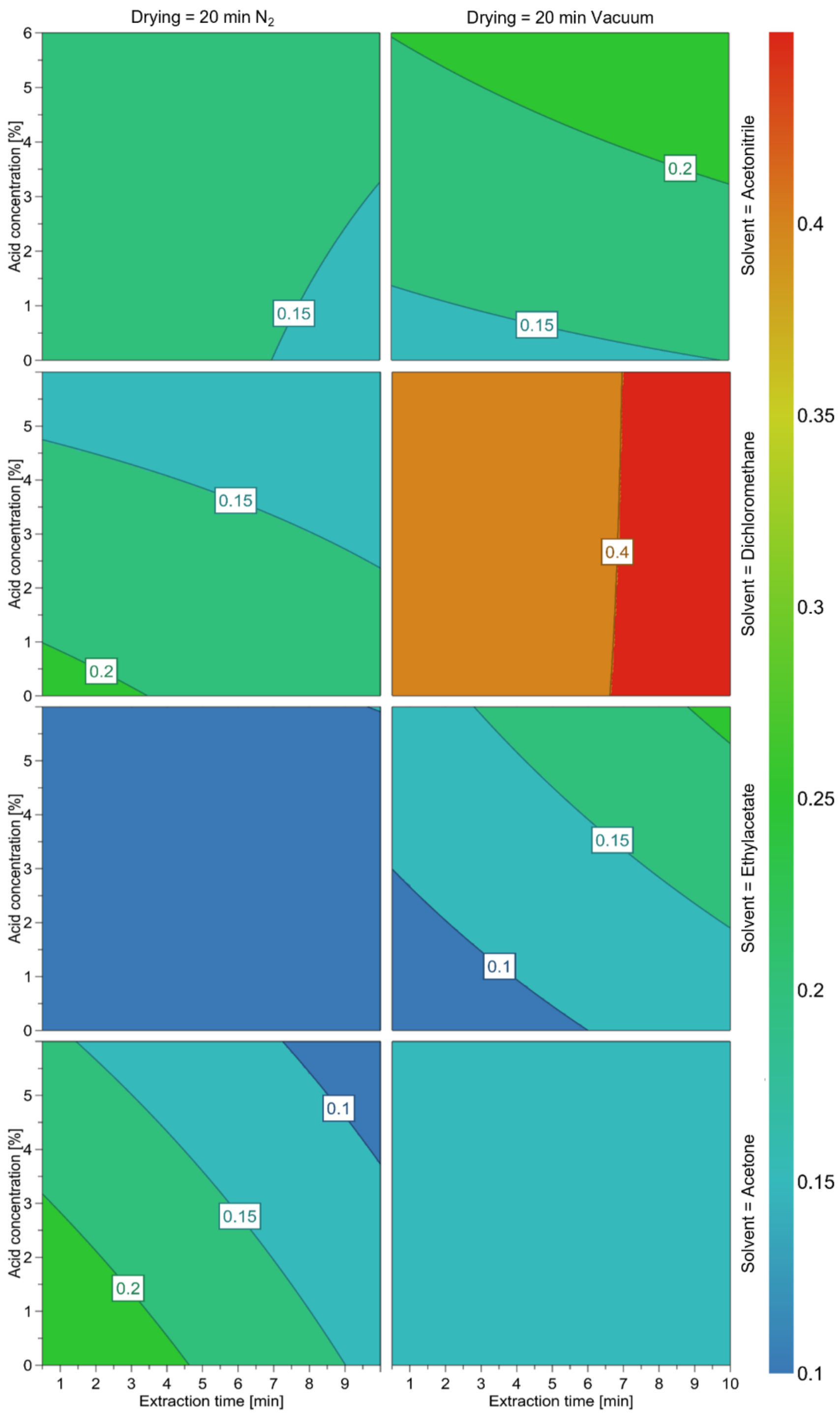

Figure S8 Contour plots for **cyclic hydrocarbons** for the optimization of the solid-phase extraction parameters acid concentration, extraction time, solvent and drying strategy.

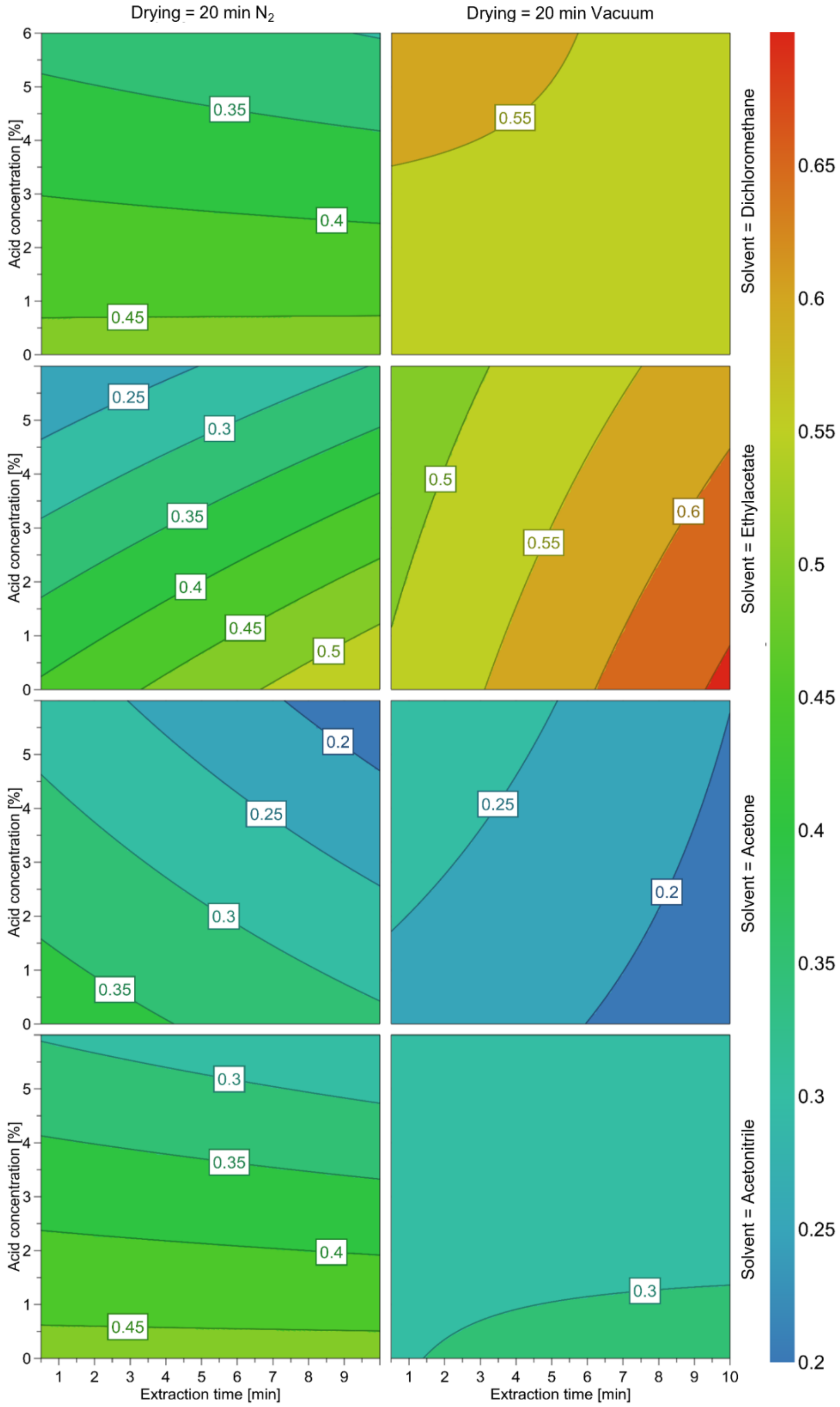

Figure S9 Contour plots for **esters** for the optimization of the solid-phase extraction parameters acid concentration, extraction time, solvent and drying strategy.

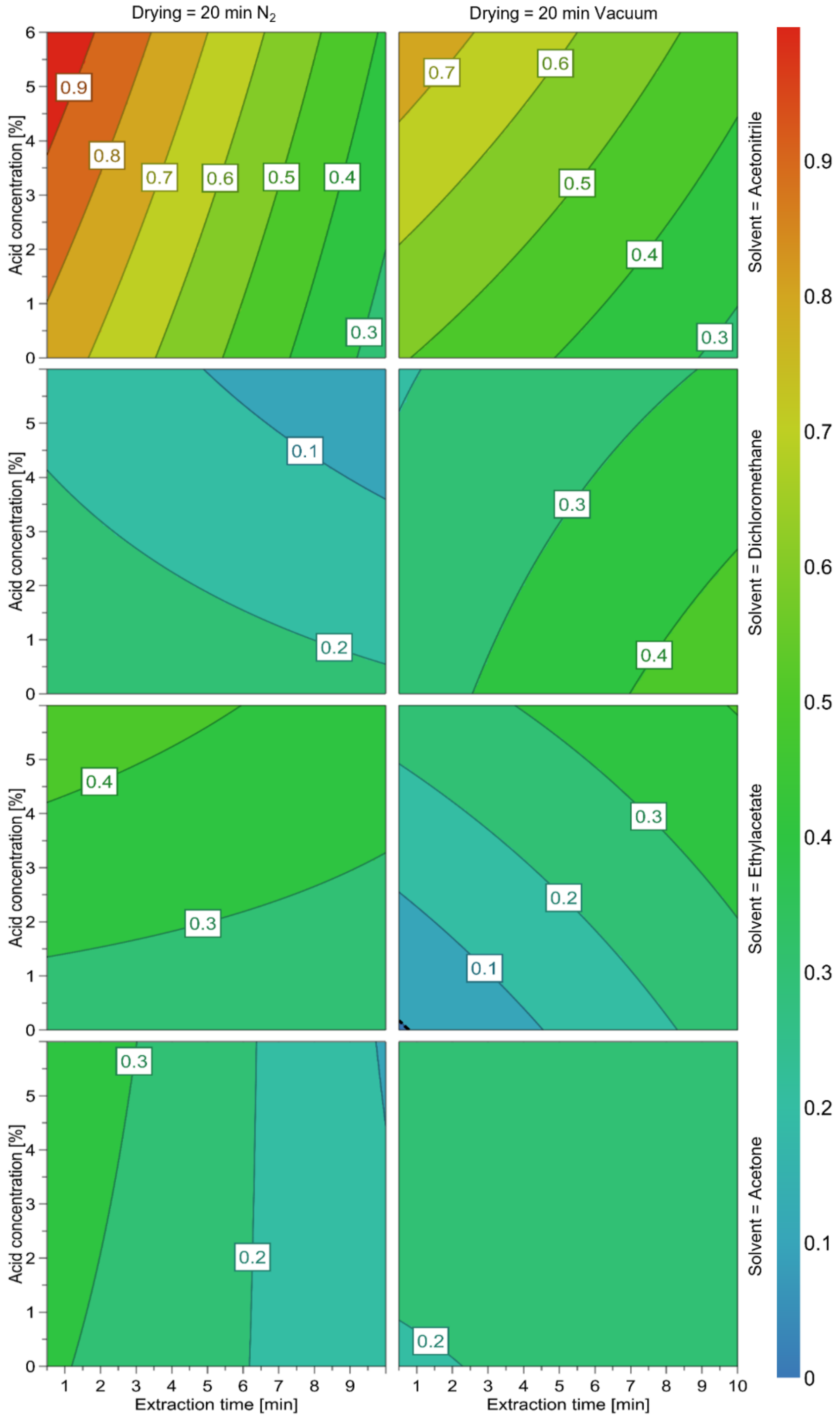

Figure S10 Contour plots for **hetero aromats** for the optimization of the solid-phase extraction parameters acid concentration, extraction time, solvent and drying strategy.

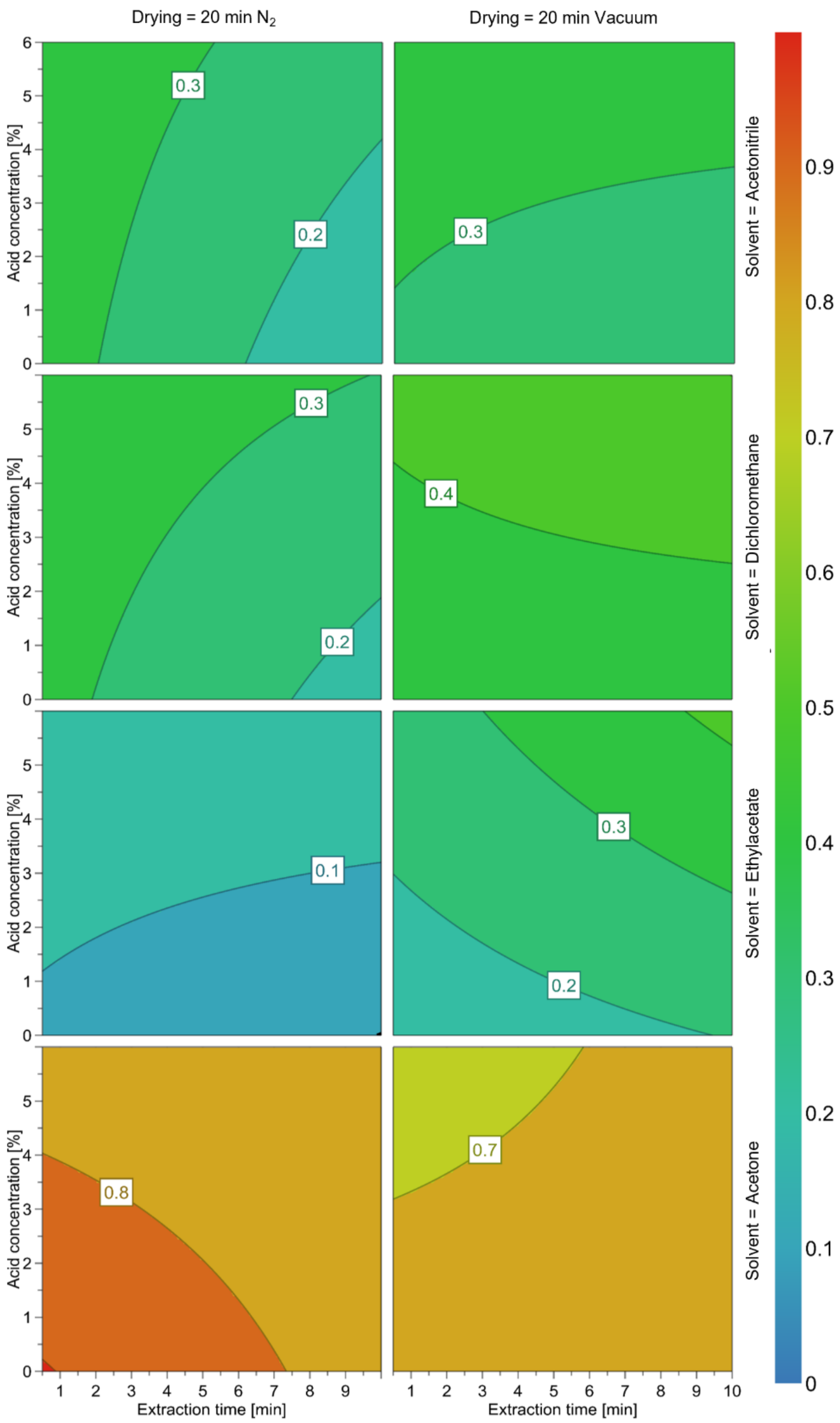

Figure S11 Contour plots for **ketones** for the optimization of the solid-phase extraction parameters acid concentration, extraction time, solvent and drying strategy.

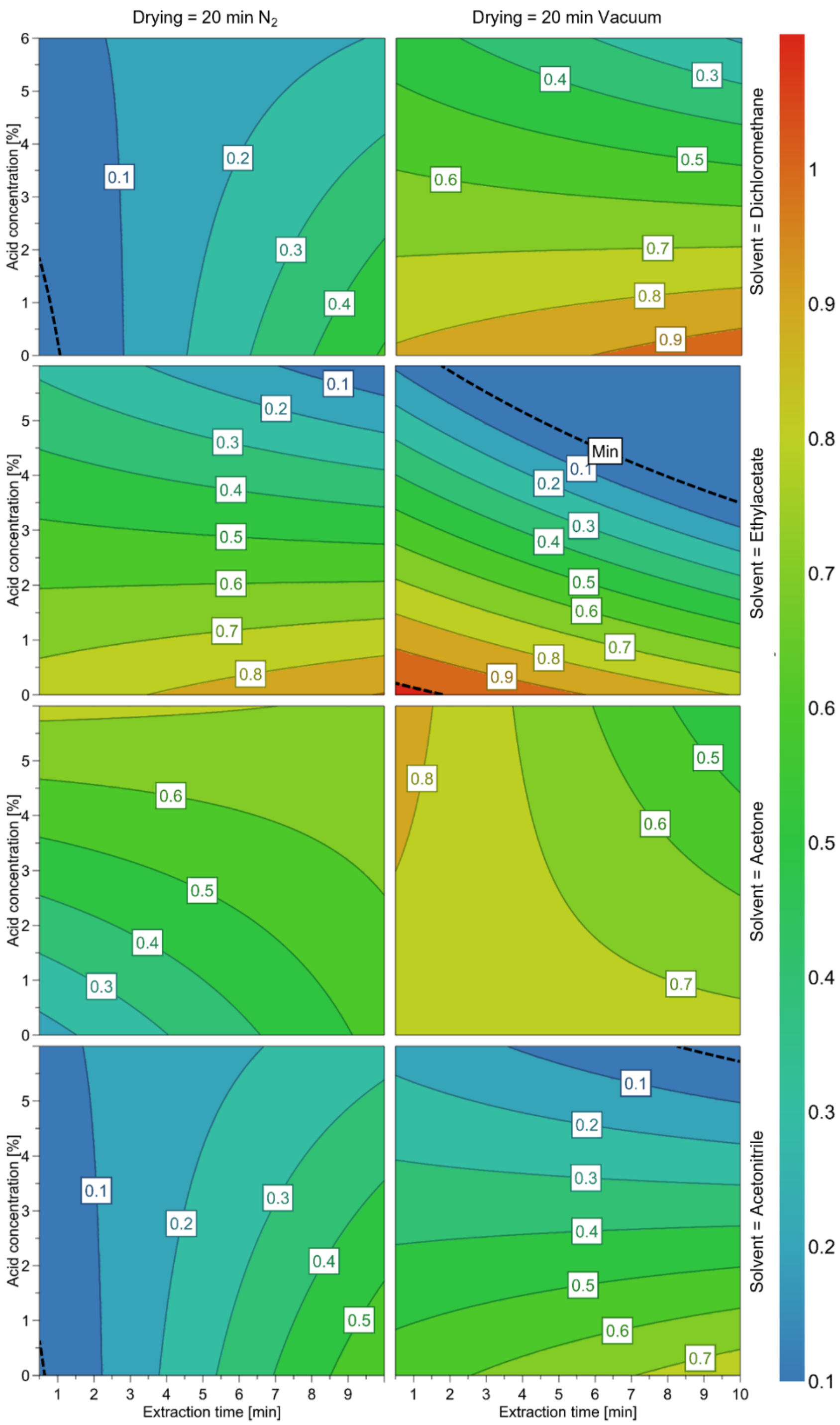

Figure S12 Contour plots for **lactams** for the optimization of the solid-phase extraction parameters acid concentration, extraction time, solvent and drying strategy.

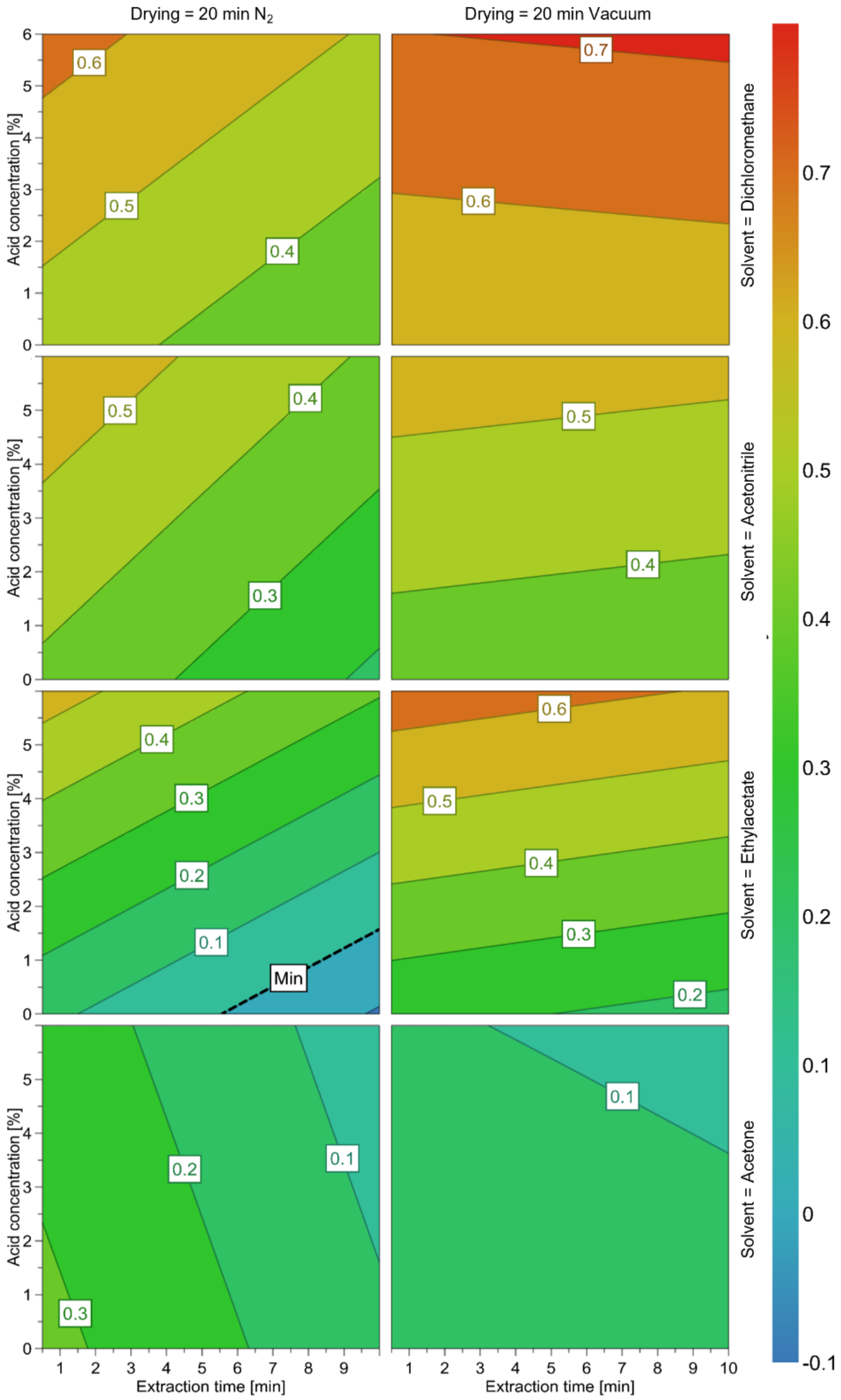

Figure S13 Contour plots for **lactones** for the optimization of the solid-phase extraction parameters acid concentration, extraction time, solvent and drying strategy.

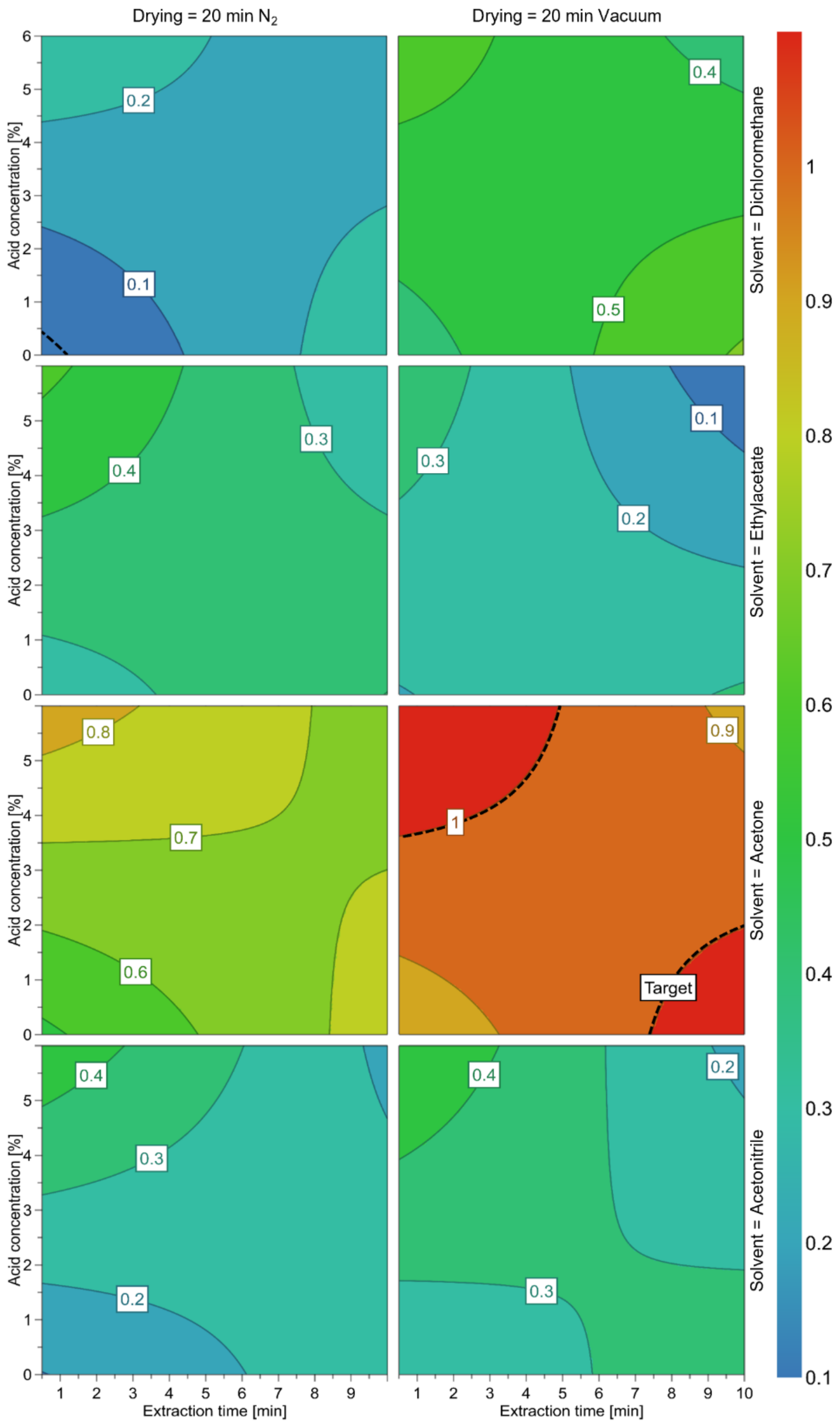

Figure S14 Contour plots for **nitriles** for the optimization of the solid-phase extraction parameters acid concentration, extraction time, solvent and drying strategy.

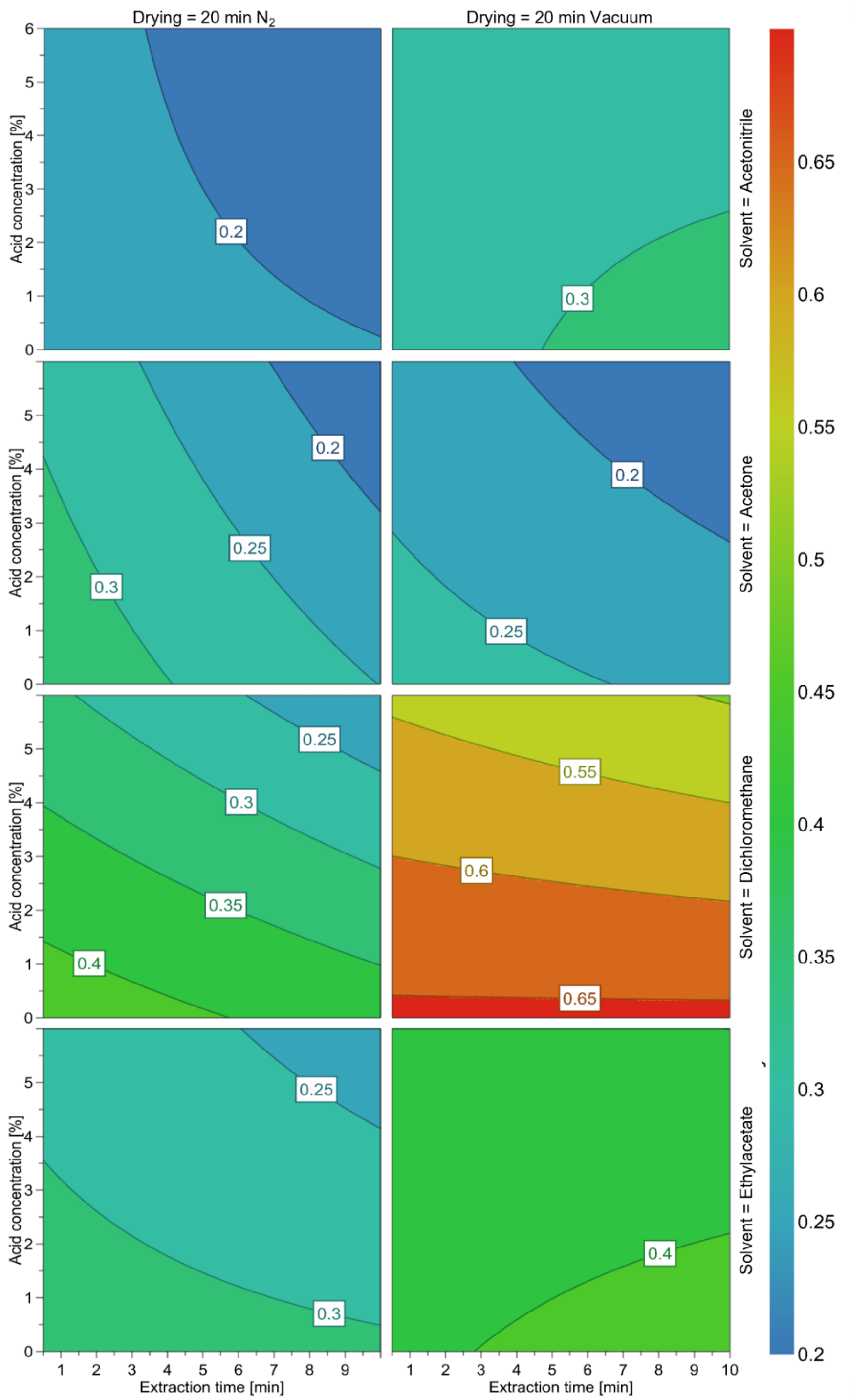

Figure S15 Contour plots for **phtalates** for the optimization of the solid-phase extraction parameters acid concentration, extraction time, solvent and drying strategy.

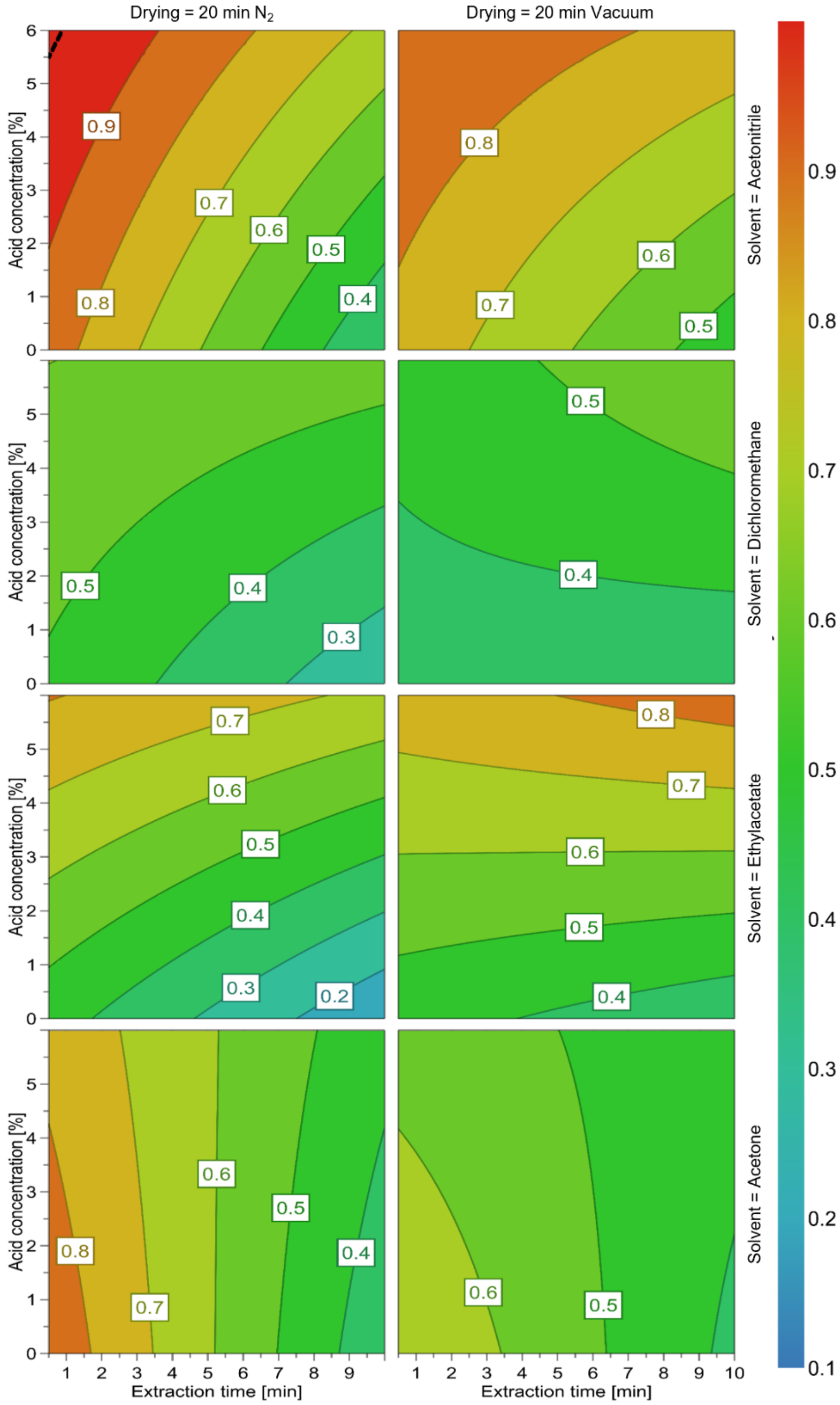

Figure S16 Contour plots for **substituted aromats** for the optimization of the solid-phase extraction parameters acid concentration, extraction time, solvent and drying strategy.

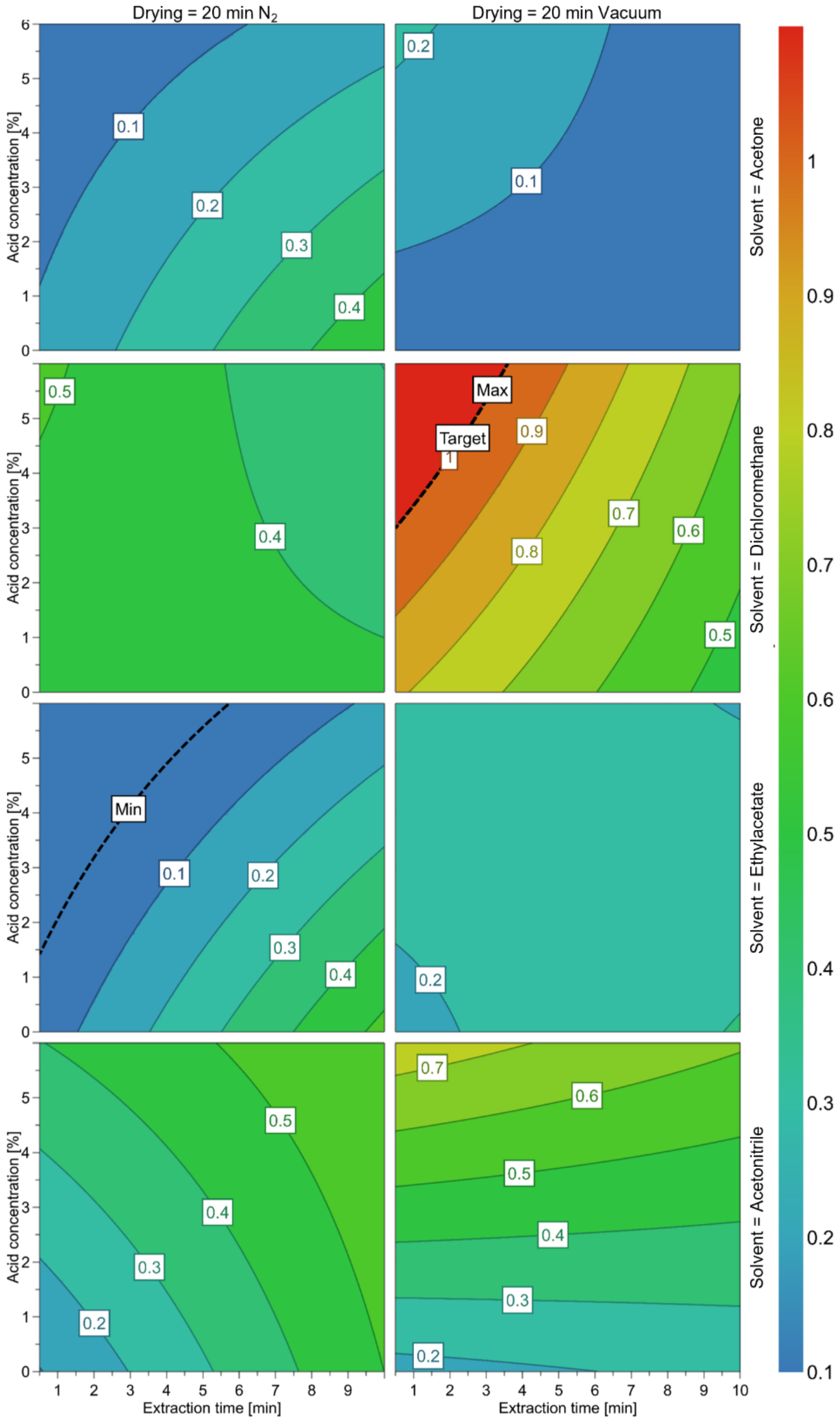

Figure S17 Contour plots for **sulfones** for the optimization of the solid-phase extraction parameters acid concentration, extraction time, solvent and drying strategy.

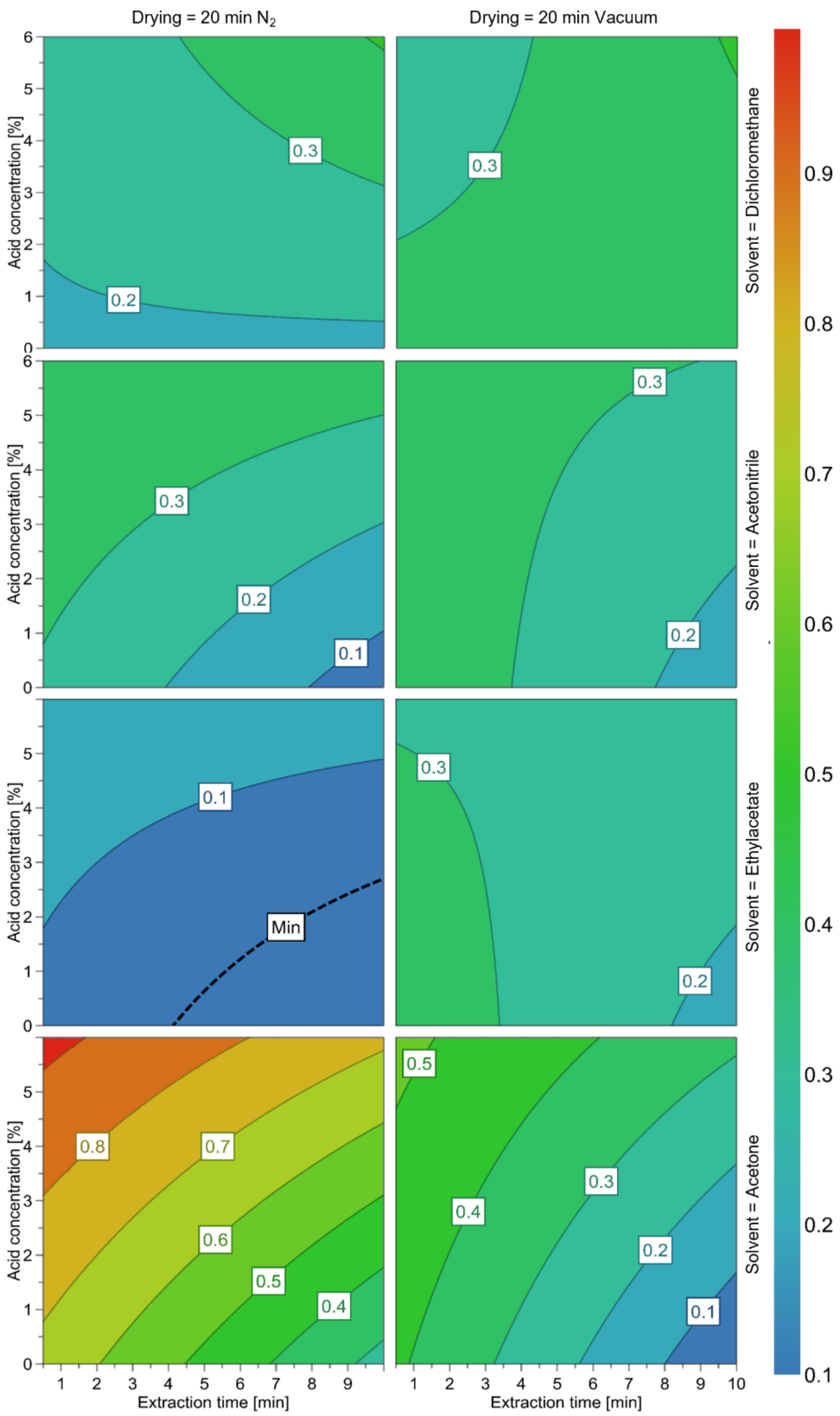

Figure S18 Contour plots for **terpenes** for the optimization of the solid-phase extraction parameters acid concentration, extraction time, solvent and drying strategy.

ITEX procedure optimization contour plots for compound groups

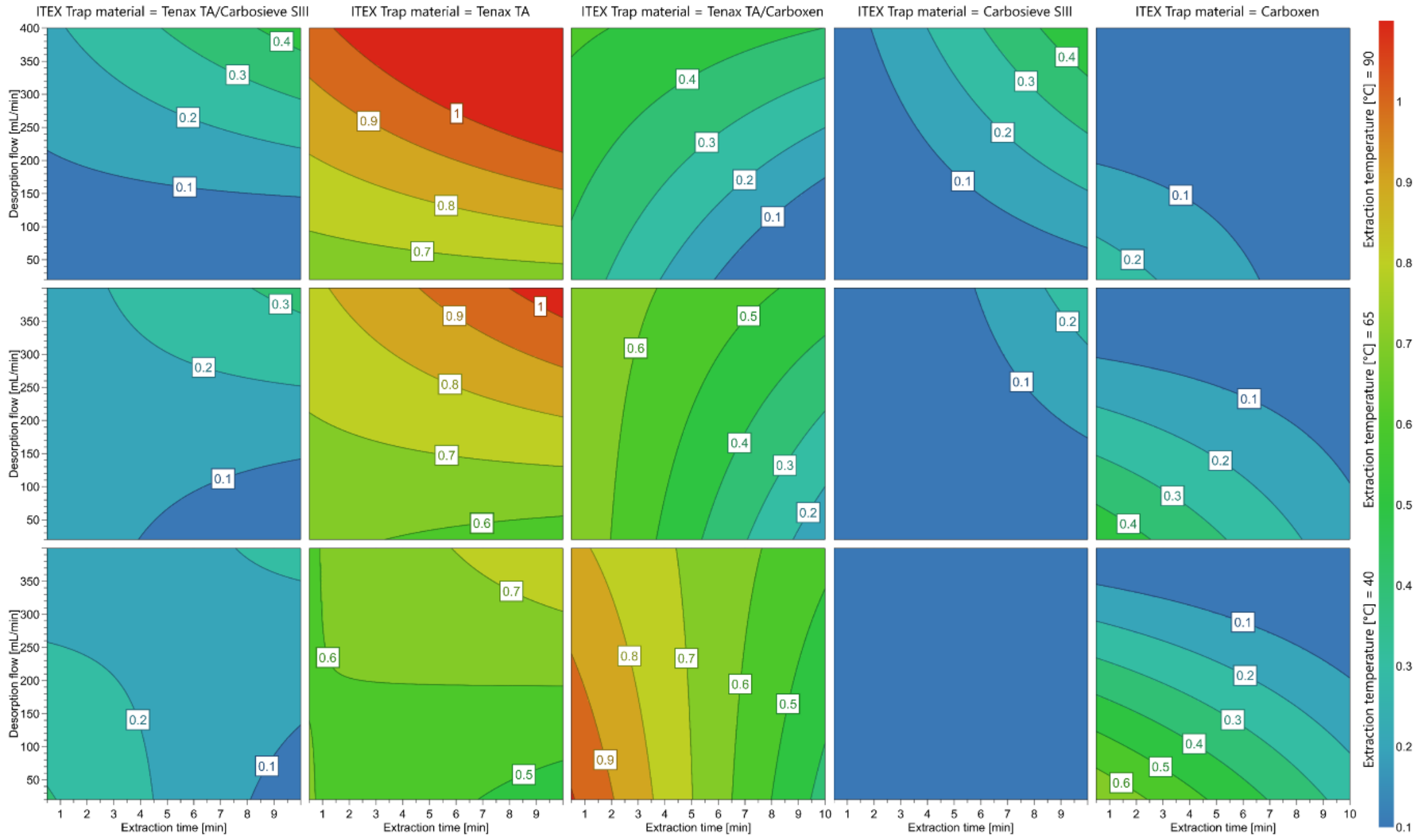

Figure S19 Contour plots for **alcohols** for the optimization of the vacuum in-tube extraction parameters desorption flow, extraction time, extraction temperature and trap material.

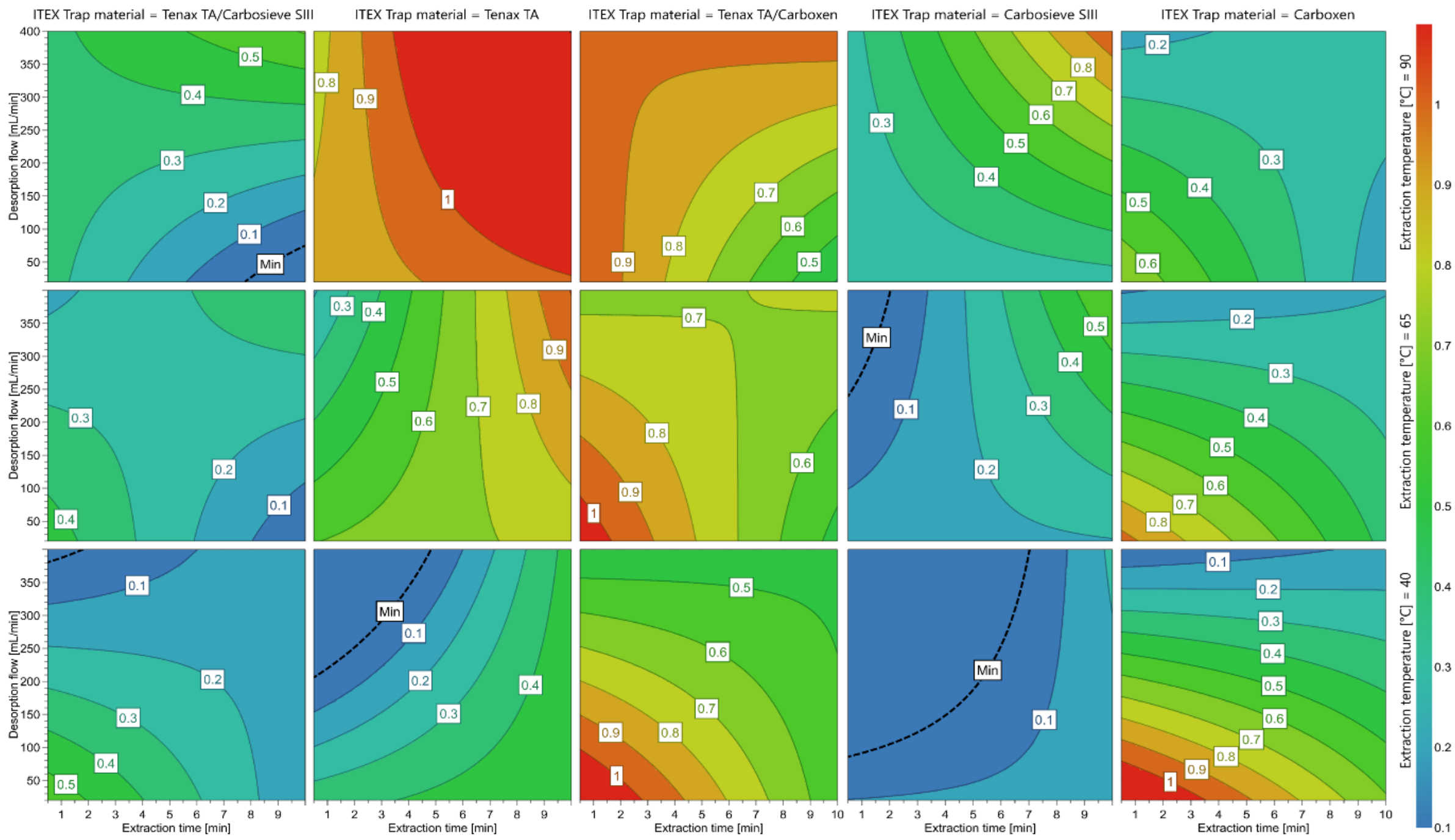

Figure S20 Contour plots for **aldehydes** for the optimization of the vacuum in-tube extraction parameters desorption flow, extraction time, extraction temperature and trap material.

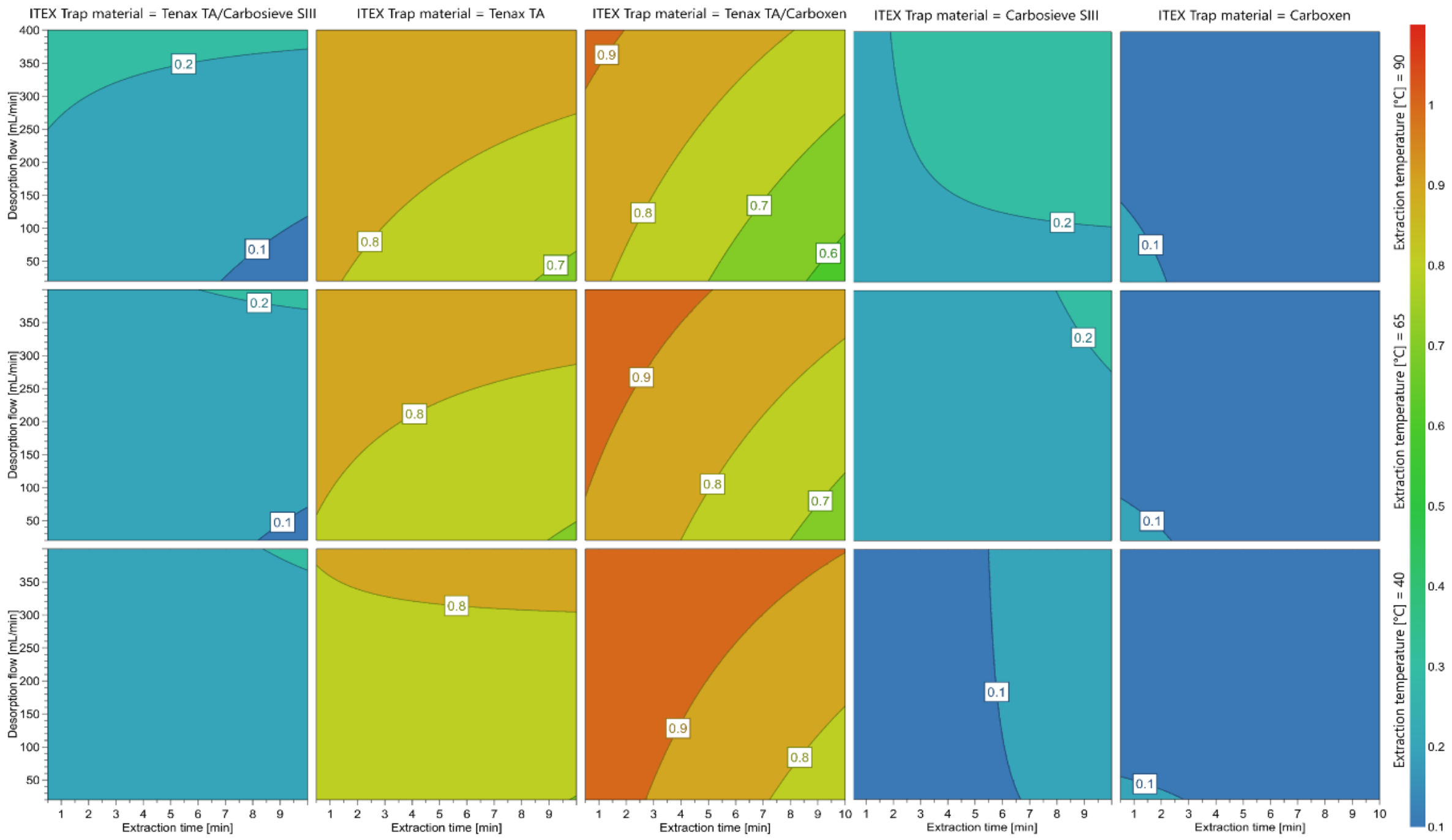

Figure S21 Contour plots for **aliphatic hydrocarbons** for the optimization of the vacuum in-tube extraction parameters desorption flow, extraction time, extraction temperature and trap material.

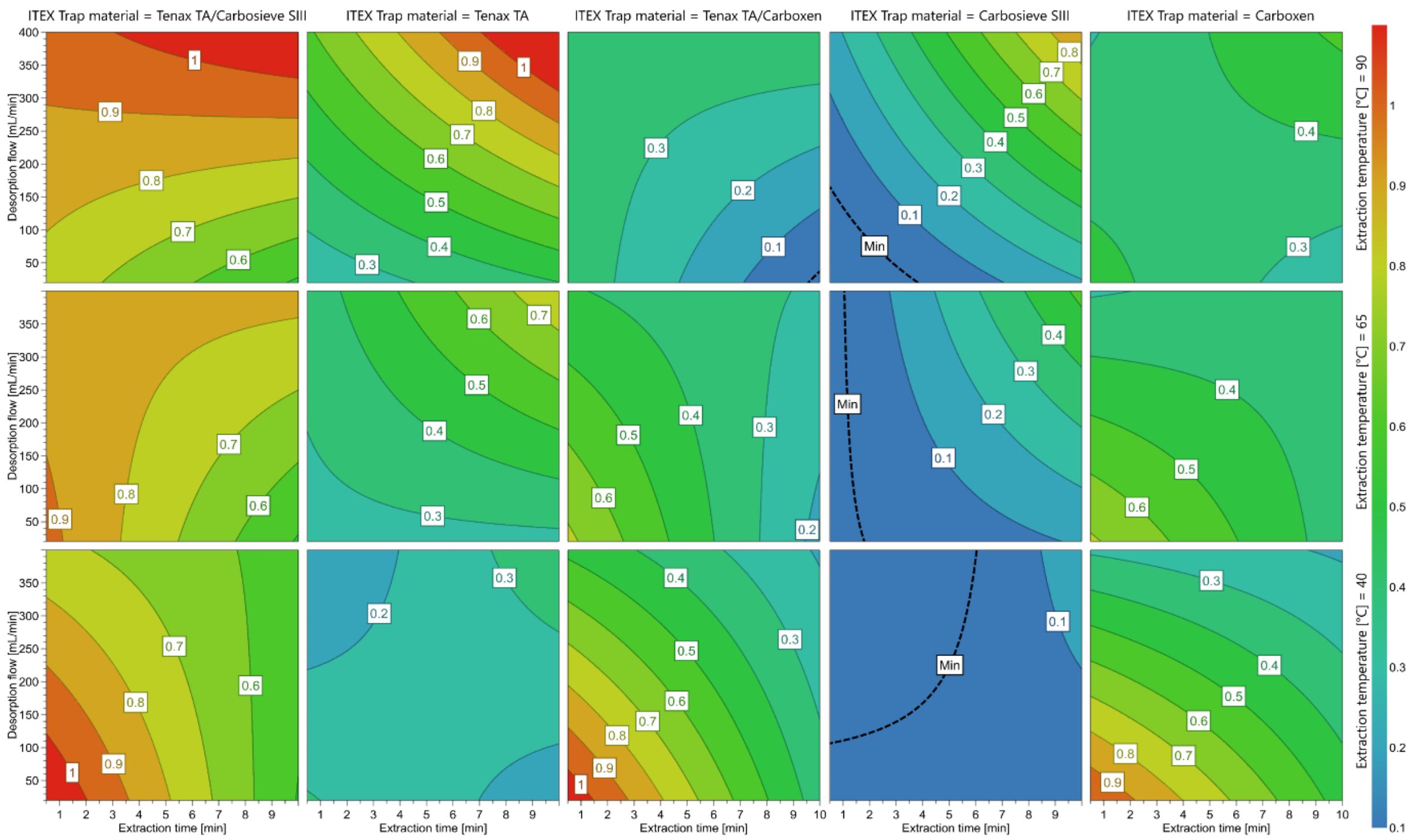

Figure S22 Contour plots for **aromatic hydrocarbons** for the optimization of the vacuum in-tube extraction parameters desorption flow, extraction time, extraction temperature and trap material.

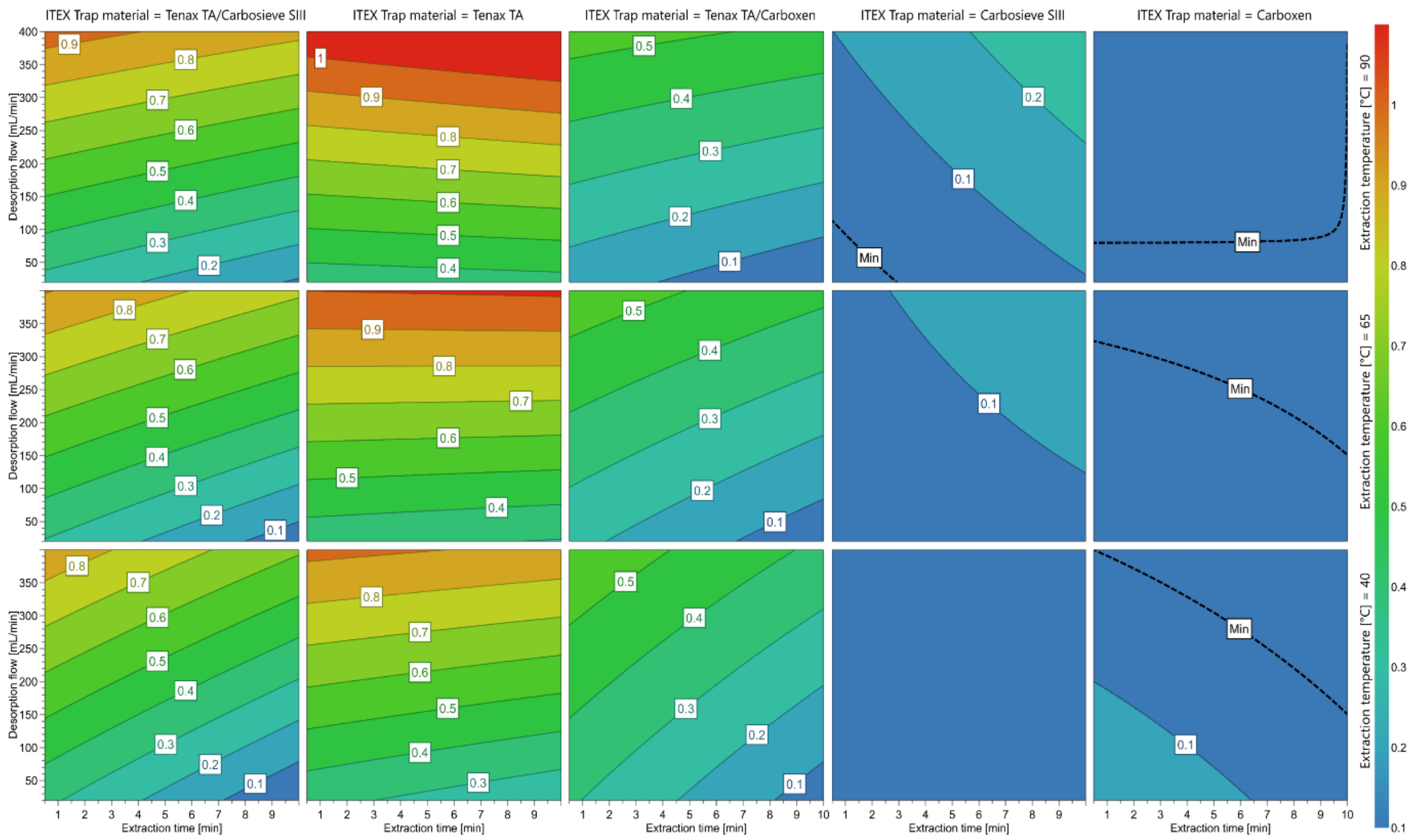

Figure S23 Contour plots for **azines** for the optimization of the vacuum in-tube extraction parameters desorption flow, extraction time, extraction temperature and trap material.

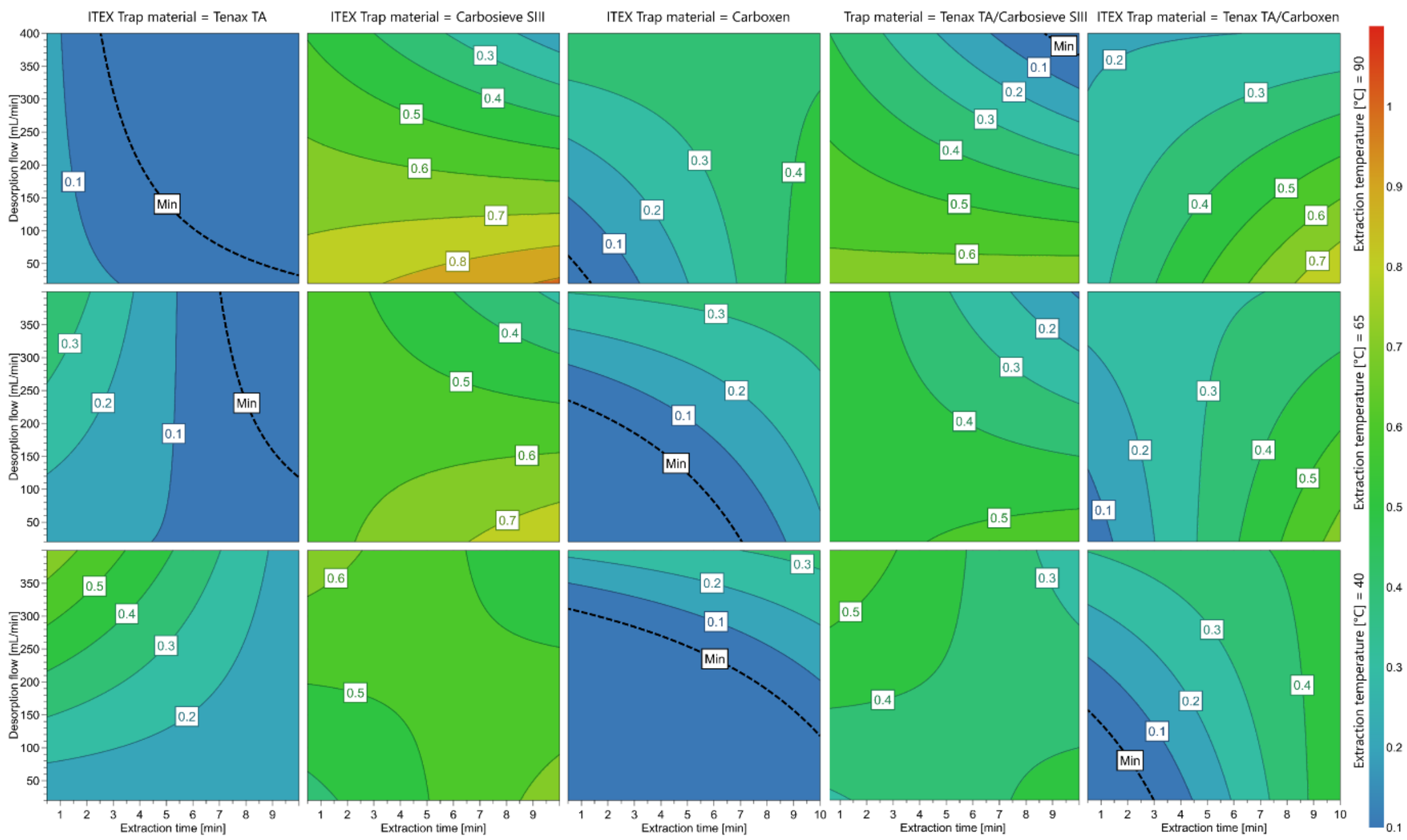

Figure S24 Contour plots for **cyclic hydrocarbons** for the optimization of the vacuum in-tube extraction parameters desorption flow, extraction time, extraction temperature and trap material.

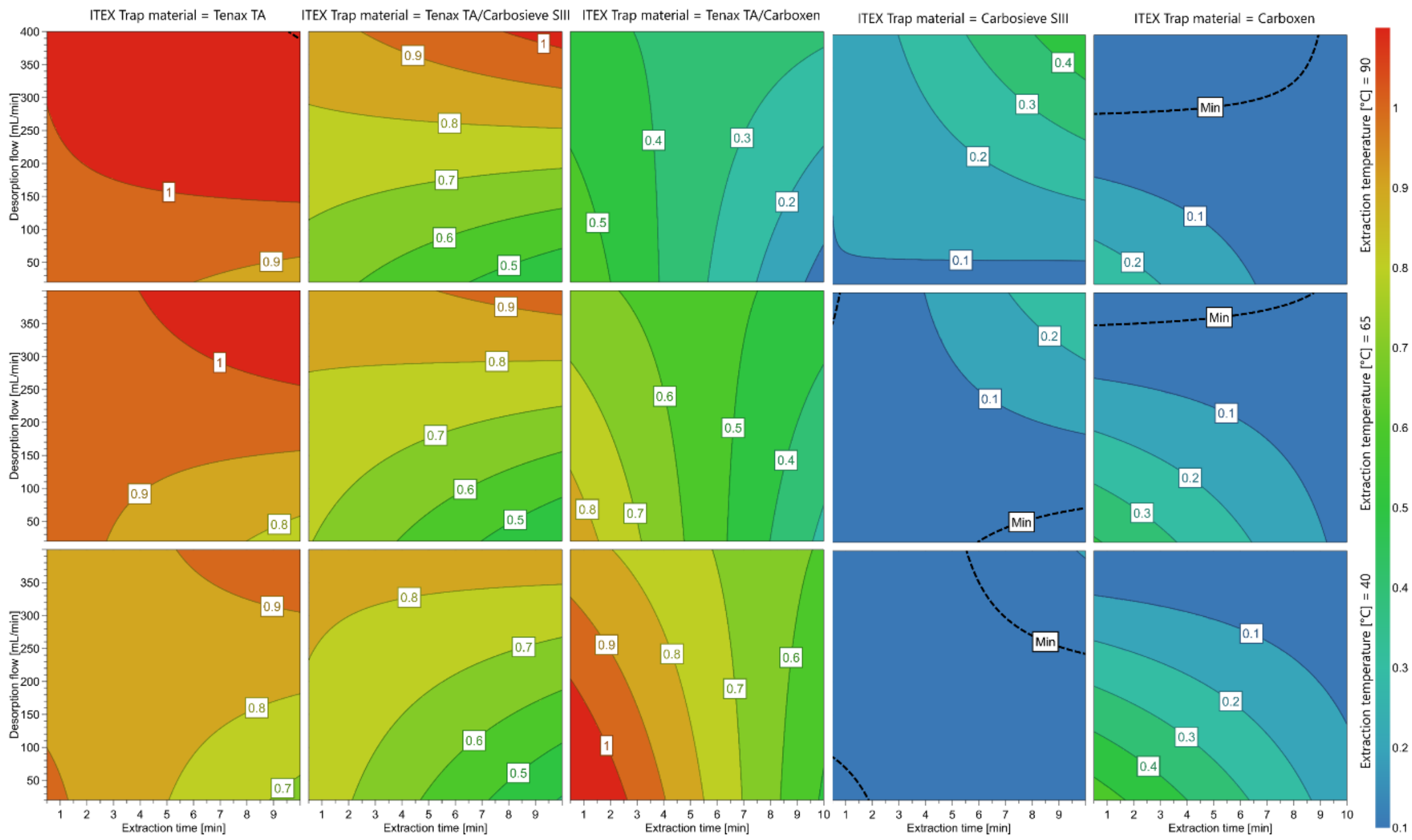

Figure S25 Contour plots for **esters** for the optimization of the vacuum in-tube extraction parameters desorption flow, extraction time, extraction temperature and trap material.

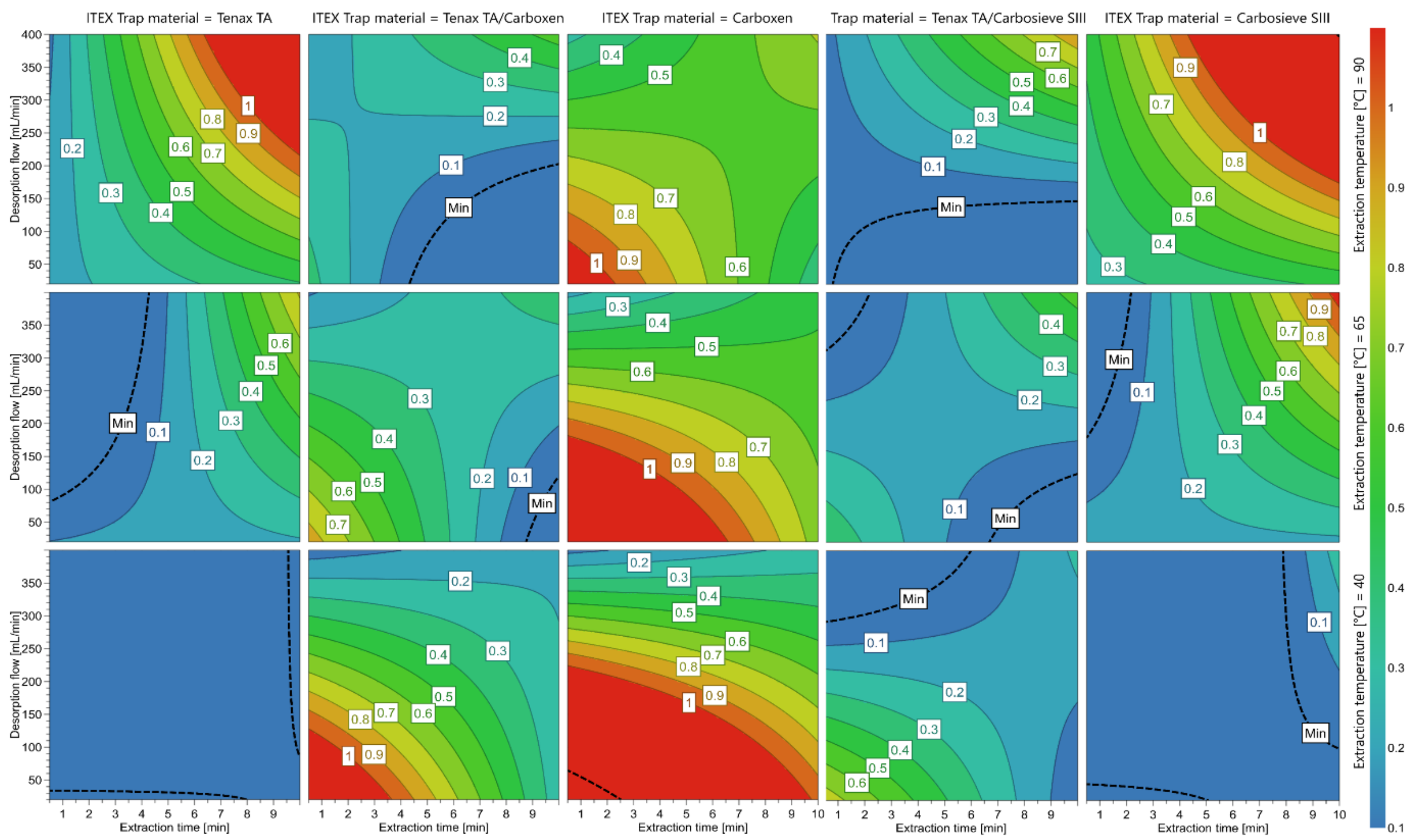

Figure S26 Contour plots for **hetero aromats** for the optimization of the vacuum in-tube extraction parameters desorption flow, extraction time, extraction temperature and trap material.

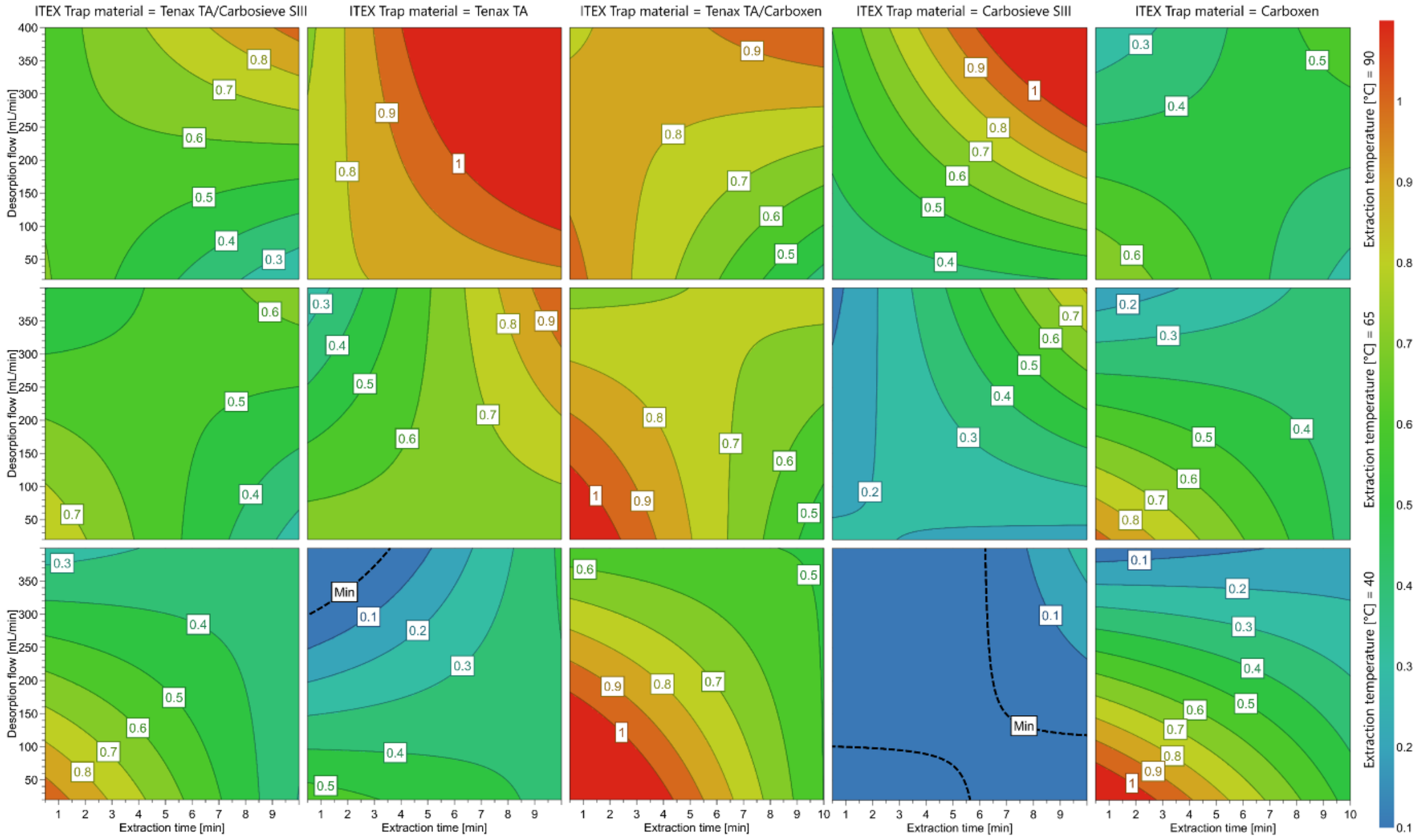

Figure S27 Contour plots for **ketones** for the optimization of the vacuum in-tube extraction parameters desorption flow, extraction time, extraction temperature and trap material.

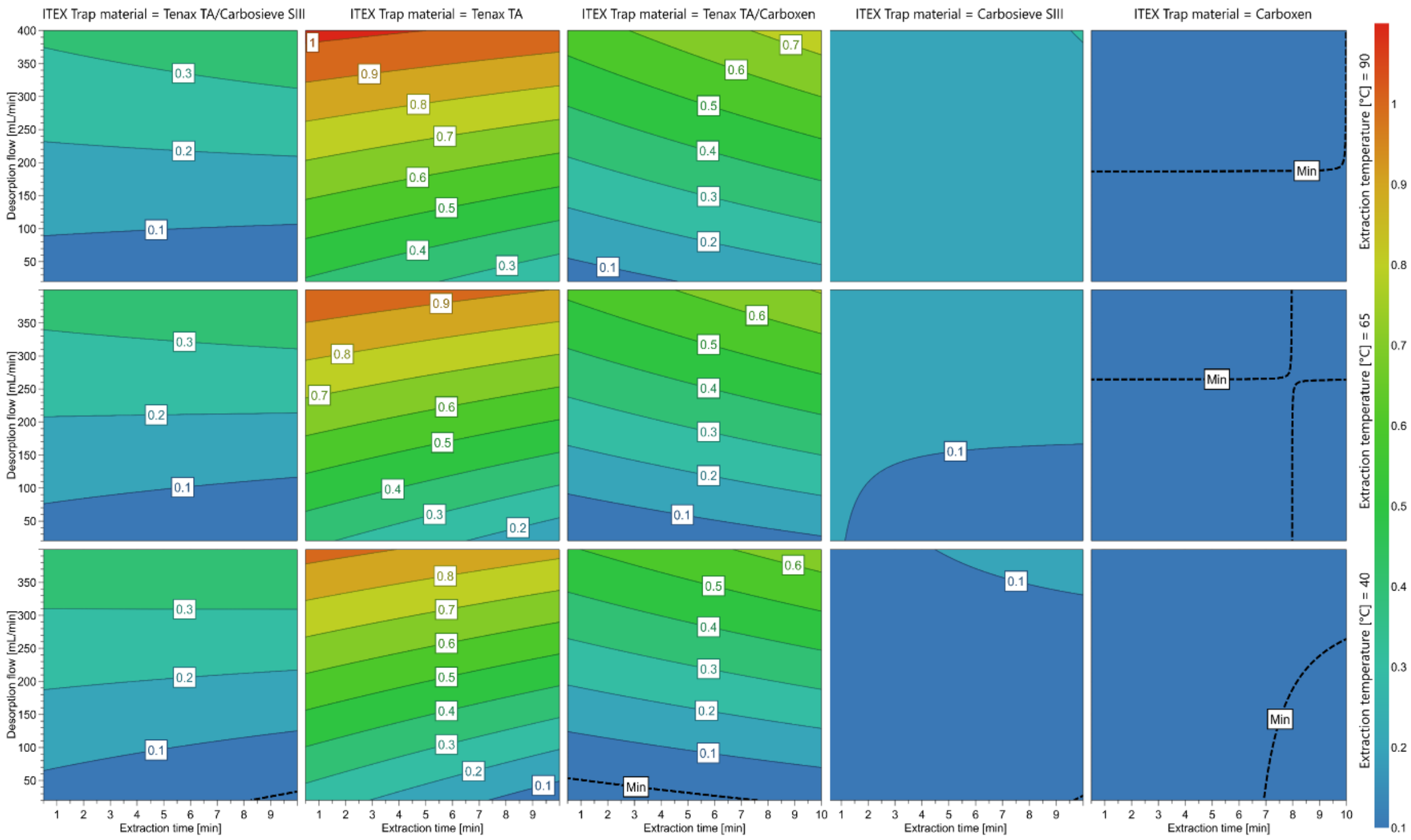

Figure S28 Contour plots for **lactams** for the optimization of the vacuum in-tube extraction parameters desorption flow, extraction time, extraction temperature and trap material.

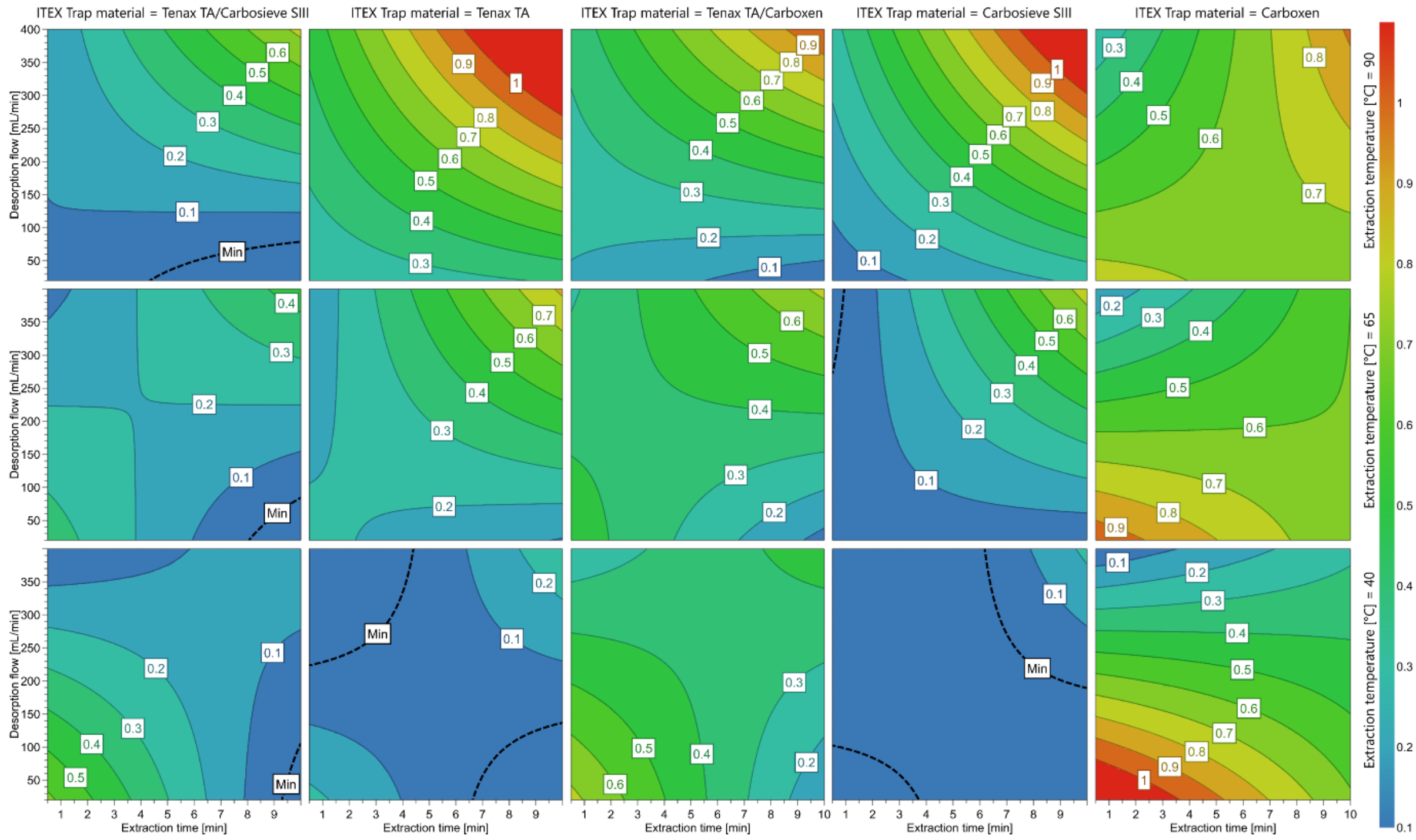

Figure S29 Contour plots for **lactones** for the optimization of the vacuum in-tube extraction parameters desorption flow, extraction time, extraction temperature and trap material.

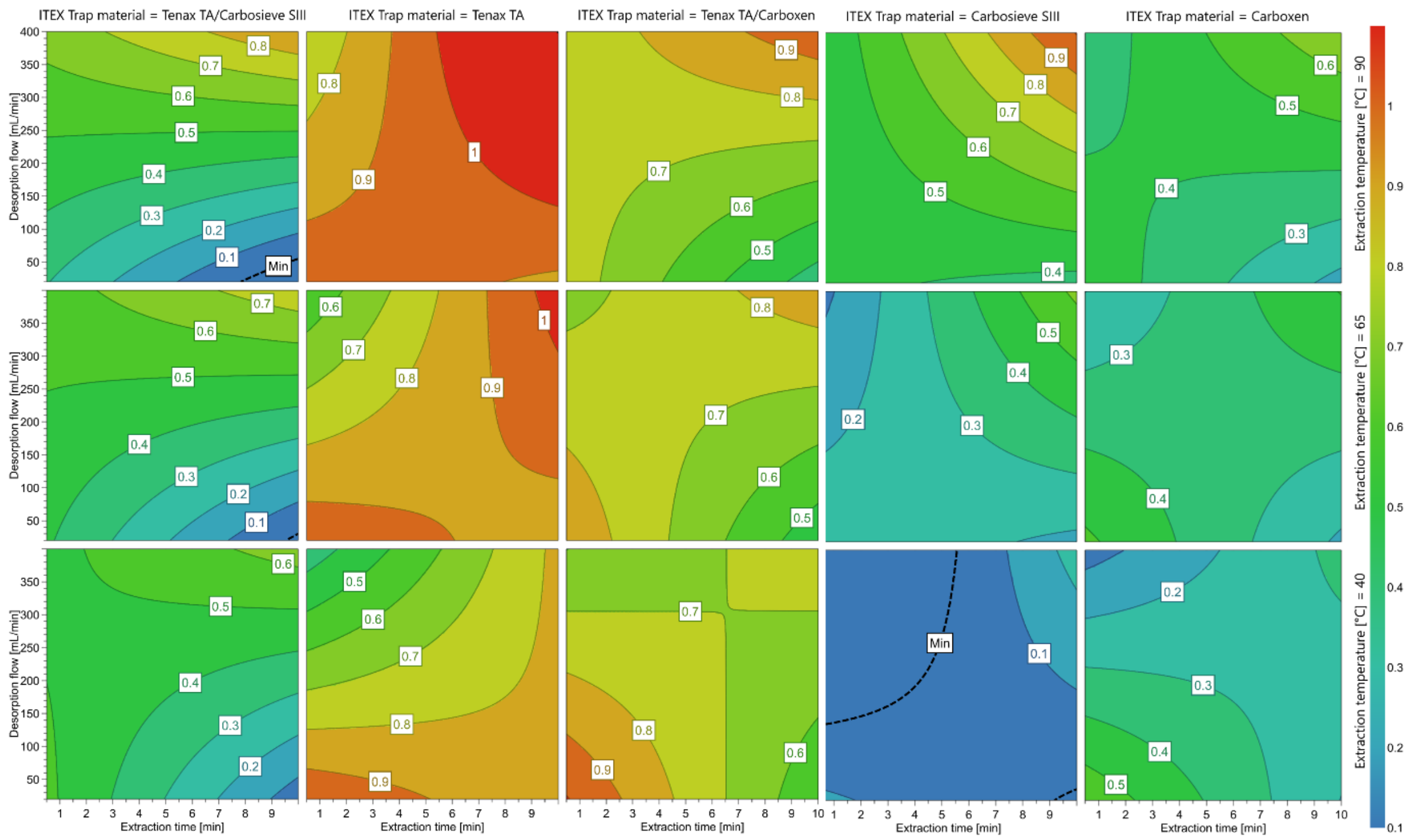

Figure S30 Contour plots for **nitriles** for the optimization of the vacuum in-tube extraction parameters desorption flow, extraction time, extraction temperature and trap material.

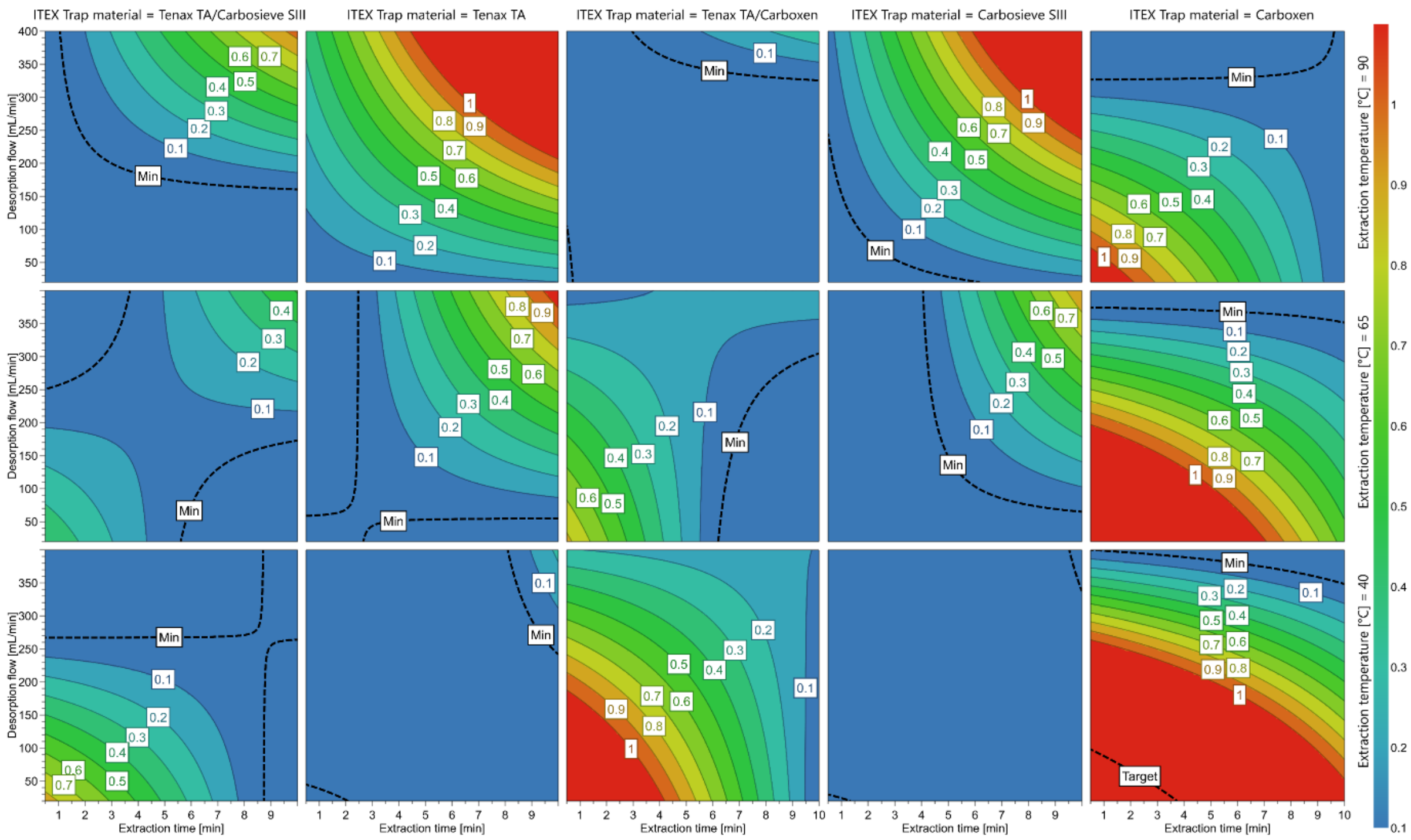

Figure S31 Contour plots for **phtalates** for the optimization of the vacuum in-tube extraction parameters desorption flow, extraction time, extraction temperature and trap material.

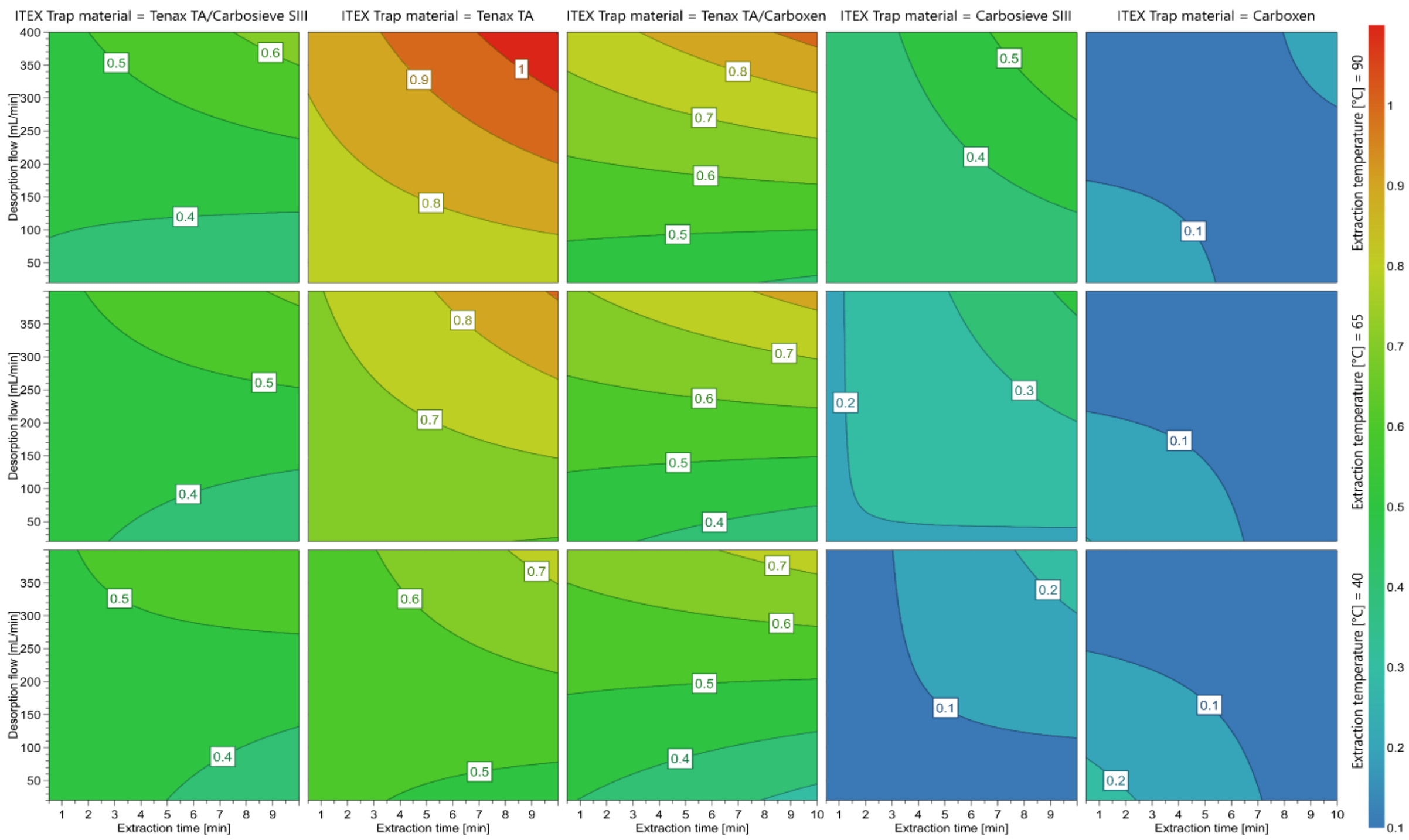

Figure S32 Contour plots for **substituted aromats** for the optimization of the vacuum in-tube extraction parameters desorption flow, extraction time, extraction temperature and trap material.

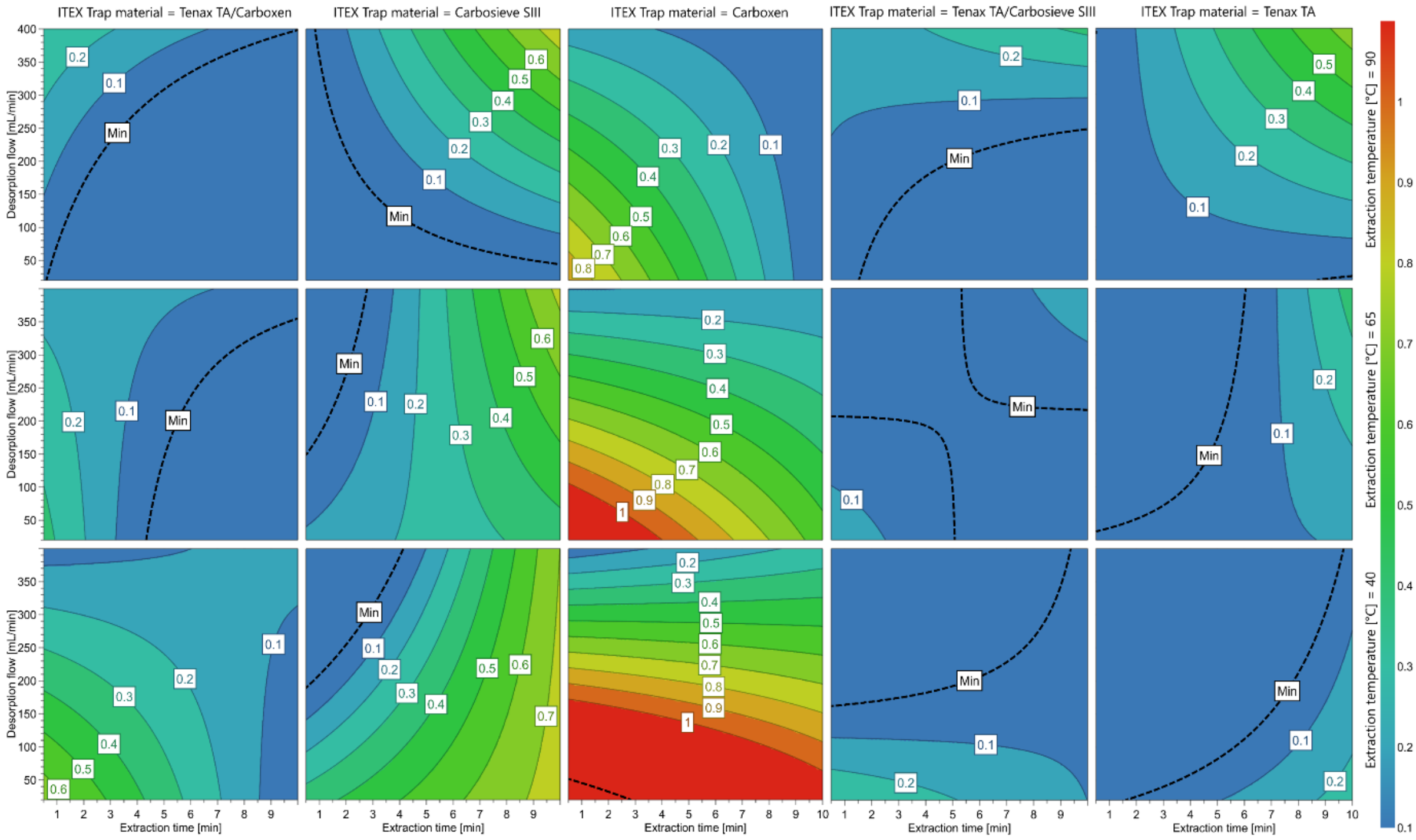

Figure S33 Contour plots for **sulfones** for the optimization of the vacuum in-tube extraction parameters desorption flow, extraction time, extraction temperature and trap material.

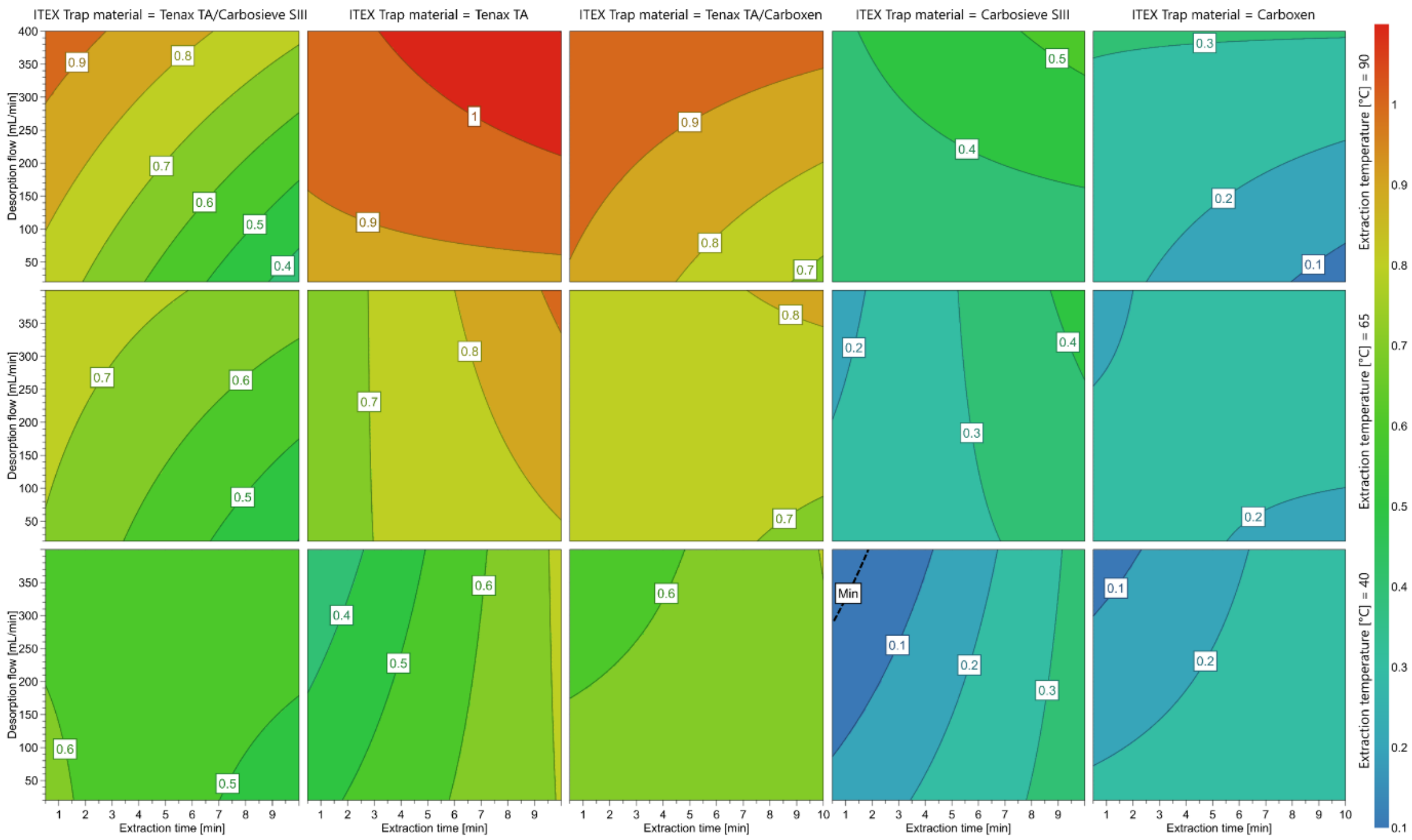

Figure S34 Contour plots for **terpenes** for the optimization of the vacuum in-tube extraction parameters desorption flow, extraction time, extraction temperature and trap material.

Correlations and significance of parameter combinations

Table S5 Table of significant parameter correlations for solid-phase extraction optimization with the Pearson correlation coefficient r. Parameters were sorted from highest correlation coefficient to lowest.

Variable 1	Variable 2	r	p-vlaue
Drying_20 min N2	Drying_20 min Vacuum	-1.0000	0.0E+00
Sum_aliphatic_hc_norm	Azines_norm	0.9753	1.4E-16
Azines_norm	Sum_phtalates_norm	0.9347	8.2E-12
Sum_aliphatic_hc_norm	Sum_phtalates_norm	0.9266	3.0E-11
Sum_aromatic_hc_norm	Sum_cyclic_hc_norm	0.9070	4.1E-10
Sum_aliphatic_hc_norm	Sum_aromatic_hc_norm	0.8940	1.7E-09
Sum_all_norm	Sum_aromatic_hc_norm	0.8864	3.7E-09
Sum_aromatic_hc_norm	Sum_phtalates_norm	0.8830	5.1E-09
Sum_aromatic_hc_norm	Azines_norm	0.8696	1.7E-08
Sum_all_norm	Sum_aldehydes_norm	0.8600	3.6E-08
Sum_cyclic_hc_norm	Sum_phtalates_norm	0.8597	3.7E-08
Sum_aromatic_hc_norm	Sum_lactones_norm	0.8596	3.7E-08
Sum_esters_norm	Sum_phtalates_norm	0.8503	7.4E-08
Sum_aldehydes_norm	Azines_norm	0.8499	7.6E-08
Sum_ketones_norm	Solvent_Acetone	0.8409	1.4E-07
Azines_norm	Sum_esters_norm	0.8407	1.4E-07
Sum_aldehydes_norm	Sum_aliphatic_hc_norm	0.8353	2.0E-07
Sum_aldehydes_norm	Sum_aromatic_hc_norm	0.8259	3.7E-07
Sum_all_norm	Sum_cyclic_hc_norm	0.8177	6.0E-07
Sum_aliphatic_hc_norm	Sum_esters_norm	0.8169	6.3E-07
Sum_aliphatic_hc_norm	Solvent_Dichloromethane	0.8055	1.2E-06
Sum_aldehydes_norm	Sum_cyclic_hc_norm	0.8022	1.4E-06
Azines_norm	Sum_cyclic_hc_norm	0.7972	1.8E-06
Sum_aldehydes_norm	Sum_phtalates_norm	0.7924	2.3E-06
Sum_all_norm	Sum_lactones_norm	0.7851	3.4E-06
Sum_aliphatic_hc_norm	Sum_cyclic_hc_norm	0.7815	4.0E-06
Azines_norm	Solvent_Dichloromethane	0.7812	4.0E-06
Sum_nitriles_norm	Solvent_Acetone	0.7799	4.3E-06
Sum_cyclic_hc_norm	Sum_esters_norm	0.7721	6.1E-06
Sum_aromatic_hc_norm	Sum_esters_norm	0.7717	6.3E-06
Sum_all_norm	Sum_phtalates_norm	0.7272	3.8E-05
Sum_aldehydes_norm	Sum_esters_norm	0.7241	4.3E-05
Sum_all_norm	Sum_aliphatic_hc_norm	0.7184	5.2E-05
Sum_cyclic_hc_norm	Sum_lactones_norm	0.7144	6.0E-05
Sum_hetero_aromats_norm	Sum_substituted_aromats_norm	0.7144	6.0E-05
Sum_all_norm	Azines_norm	0.7081	7.5E-05
Sum_ketones_norm	Sum_nitriles_norm	0.7055	8.2E-05
Sum_aliphatic_hc_norm	Sum_lactones_norm	0.6983	1.0E-04
Sum_lactones_norm	Sum_phtalates_norm	0.6933	1.2E-04
Sum_alcohols_norm	Solvent_Acetone	0.6709	2.4E-04
Sum_lactones_norm	Sulfones_norm	0.6557	3.7E-04
Sum_phtalates_norm	Solvent_Dichloromethane	0.6498	4.4E-04
Sum_aromatic_hc_norm	Sulfones_norm	0.6484	4.6E-04
Sulfones_norm	Solvent_Dichloromethane	0.6345	6.6E-04
Azines_norm	Sum_lactones_norm	0.6307	7.3E-04
Sum_ketones_norm	Sum_terpenes_norm	0.6220	9.0E-04
Sum_aliphatic_hc_norm	Sulfones_norm	0.6196	9.6E-04
Sum_aldehydes_norm	Sum_lactones_norm	0.6187	9.8E-04
Azines_norm	Sulfones_norm	0.6170	0.001
Sum_aromatic_hc_norm	Solvent_Dichloromethane	0.6150	0.001
Lactams_norm	Sum_substituted_aromats_norm	-0.6101	0.001
Sum_cyclic_hc_norm	Sulfones_norm	0.6082	0.001
Sum_all_norm	Sum_terpenes_norm	0.6062	0.001
Sum_phtalates_norm	Sulfones_norm	0.5969	0.002
Sum_all_norm	Sum_esters_norm	0.5895	0.002
Sum_hetero_aromats_norm	Solvent_Acetonitril	0.5765	0.003
Sum_esters_norm	Sum_lactones_norm	0.5732	0.003

Sum_aldehydes_norm	Solvent_Dichloromethane	0.5565	0.004
Sum_alcohols_norm	Sulfones_norm	-0.5554	0.004
Sum_all_norm	Sum_substituted_aromats_norm	0.5520	0.004
Sum_lactones_norm	Solvent_Acetone	-0.5475	0.005
Sum_alcohols_norm	Sum_ketones_norm	0.5455	0.005
Sum_lactones_norm	Solvent_Dichloromethane	0.5417	0.005
Sulfones_norm	Solvent_Acetone	-0.5314	0.006
Acid concentration	Lactams_norm	-0.5261	0.007
Sum_aldehydes_norm	Solvent_Acetonitril	-0.5191	0.008
Sum_substituted_aromats_norm	Sum_terpenes_norm	0.5176	0.008
Sum_esters_norm	Sulfones_norm	0.5070	0.010
Sum_alcohols_norm	Solvent_Dichloromethane	-0.4957	0.012
Extraction time	Sum_substituted_aromats_norm	-0.4951	0.012
Sum_esters_norm	Solvent_Dichloromethane	0.4578	0.021
Sum_cyclic_hc_norm	Solvent_Dichloromethane	0.4572	0.022
Sum_alcohols_norm	Sum_lactones_norm	-0.4479	0.025
Sum_esters_norm	Solvent_Acetone	-0.4454	0.026
Sum_aldehydes_norm	Sum_terpenes_norm	0.4435	0.026
Acid concentration	Sum_substituted_aromats_norm	0.4372	0.029
Sum_all_norm	Sum_ketones_norm	0.4363	0.029
Azines_norm	Solvent_Acetonitril	-0.4353	0.030
Sum_lactones_norm	Sum_substituted_aromats_norm	0.4344	0.030
Sum_aliphatic_hc_norm	Solvent_Acetonitril	-0.4243	0.035
Sum_alcohols_norm	Sum_terpenes_norm	0.4201	0.037
Sum_terpenes_norm	Solvent_Acetone	0.4151	0.039
Sum_ketones_norm	Solvent_Ethylacetat	-0.4101	0.042
Lactams_norm	Sum_lactones_norm	-0.3999	0.048

Table S6 Table of significant parameter correlations for vacuum in-tube extraction optimization with the Pearson correlation coefficient r. Parameters were sorted from highest correlation coefficient to lowest.

**Variable 1**	**Variable 2**	**r**	**p-vlaue**
Sum_all_norm	Sum_nitriles_norm	0.9178	2.4E-12
Sum_all_norm	Sum_ketones_norm	0.9093	8.7E-12
Sum_all_norm	Sum_substituted_aromats_norm	0.8946	6.1E-11
Sum_aldehydes_norm	Sum_ketones_norm	0.8847	1.9E-10
Sum_substituted_aromats_norm	Sum_terpenes_norm	0.8759	4.9E-10
Sum_all_norm	Sum_terpenes_norm	0.8732	6.5E-10
Azines_norm	Lactams_norm	0.8675	1.1E-09
Sum_ketones_norm	Sum_nitriles_norm	0.8605	2.2E-09
Lactams_norm	Sum_substituted_aromats_norm	0.8596	2.3E-09
Sum_alcohols_norm	Sum_aliphatic_hc_norm	0.8577	2.8E-09
Sum_all_norm	Sum_aldehydes_norm	0.8425	9.9E-09
Sum_ketones_norm	Sum_terpenes_norm	0.8411	1.1E-08
Sum_nitriles_norm	Sum_substituted_aromats_norm	0.8197	5.3E-08
Azines_norm	Sum_esters_norm	0.8161	6.8E-08
Sum_aliphatic_hc_norm	Sum_substituted_aromats_norm	0.8108	9.7E-08
Sum_aldehydes_norm	Sum_nitriles_norm	0.8040	1.5E-07
Sum_nitriles_norm	Sum_terpenes_norm	0.8035	1.5E-07
Sum_aliphatic_hc_norm	Sum_nitriles_norm	0.7964	2.4E-07
Sum_all_norm	Sum_aliphatic_hc_norm	0.7862	4.3E-07
Azines_norm	Sum_substituted_aromats_norm	0.7807	5.9E-07
Sum_hetero_aromats_norm	Sum_lactones_norm	0.7777	6.9E-07
Sum_aldehydes_norm	Sum_terpenes_norm	0.7749	8.0E-07
Sum_esters_norm	Sum_terpenes_norm	0.7721	9.3E-07
Sum_esters_norm	Sum_substituted_aromats_norm	0.7633	1.5E-06
Sum_alcohols_norm	Sum_esters_norm	0.7561	2.1E-06
Sum_all_norm	Lactams_norm	0.7527	2.5E-06
Sum_aliphatic_hc_norm	Sum_terpenes_norm	0.7508	2.7E-06
Sum_alcohols_norm	ITEX Trap material_Tenax TA	0.7498	2.8E-06
Sum_ketones_norm	Sum_substituted_aromats_norm	0.7483	3.1E-06
Sum_aromatic_hc_norm	ITEX Trap material_Tenax TA/Carbosieve SIII	0.7364	5.3E-06

Sum_hetero_aromats_norm	Sum_phtalates_norm	0.7317	6.5E-06
Sum_alcohols_norm	Lactams_norm	0.7239	9.1E-06
Sum_aliphatic_hc_norm	Lactams_norm	0.7236	9.2E-06
Sum_aldehydes_norm	Sum_aliphatic_hc_norm	0.7148	1.3E-05
Azines_norm	Sum_terpenes_norm	0.7093	1.6E-05
Sum_aliphatic_hc_norm	Sum_esters_norm	0.6961	2.7E-05
Sum_alcohols_norm	Sum_nitriles_norm	0.6947	2.9E-05
Sum_all_norm	Sum_alcohols_norm	0.6945	2.9E-05
Sum_alcohols_norm	Sum_substituted_aromats_norm	0.6934	3.0E-05
Sum_lactones_norm	Sum_phtalates_norm	0.6897	3.5E-05
Sum_all_norm	Sum_esters_norm	0.6762	5.7E-05
Lactams_norm	Sum_terpenes_norm	0.6741	6.1E-05
Sum_aldehydes_norm	Sum_substituted_aromats_norm	0.6722	6.5E-05
Sum_aliphatic_hc_norm	Sum_ketones_norm	0.6675	7.6E-05
Sum_all_norm	Azines_norm	0.6668	7.8E-05
Sum_esters_norm	Sum_nitriles_norm	0.6540	1.2E-04
Lactams_norm	Sum_nitriles_norm	0.6516	1.3E-04
Sum_esters_norm	Lactams_norm	0.6480	1.4E-04
Sum_alcohols_norm	Azines_norm	0.6379	2.0E-04
Sum_esters_norm	ITEX Trap material_Tenax TA	0.6346	2.2E-04
Sum_alcohols_norm	Sum_aldehydes_norm	0.6216	3.2E-04
Sum_hetero_aromats_norm	ITEX Trap material_Carboxen	0.6166	3.7E-04
Sum_alcohols_norm	Sum_terpenes_norm	0.6165	3.7E-04
Sum_aliphatic_hc_norm	ITEX Trap material_Tenax TA	0.6069	4.8E-04
Sum_aromatic_hc_norm	Azines_norm	0.5998	5.8E-04
Sum_all_norm	Sum_lactones_norm	0.5901	7.5E-04
Sum_aromatic_hc_norm	ITEX Trap material_Carbosieve SIII	-0.5874	8.1E-04
Sum_substituted_aromats_norm	ITEX Trap material_Carboxen	-0.5856	8.5E-04
Sum_ketones_norm	Sum_lactones_norm	0.5820	9.3E-04
Sum_esters_norm	ITEX Trap material_Carboxen	-0.5811	9.5E-04
Sum_aliphatic_hc_norm	Azines_norm	0.5763	0.001
Sum_aliphatic_hc_norm	ITEX Trap material_Tenax TA/Carboxen	0.5735	0.001
Azines_norm	Sum_nitriles_norm	0.5640	0.001
Sum_nitriles_norm	ITEX Trap material_Tenax TA	0.5569	0.002
Sum_aldehydes_norm	Sum_lactones_norm	0.5529	0.002
Lactams_norm	ITEX Trap material_Tenax TA	0.5466	0.002
Sum_aromatic_hc_norm	Sum_esters_norm	0.5439	0.002
Sum_terpenes_norm	ITEX Trap material_Carboxen	-0.5424	0.002
Sum_substituted_aromats_norm	ITEX Trap material_Tenax TA	0.5196	0.004
Sum_esters_norm	Sum_ketones_norm	0.5186	0.004
Sum_alcohols_norm	Sum_ketones_norm	0.5073	0.005
Sum_lactones_norm	Sum_nitriles_norm	0.5044	0.005
Sum_aldehydes_norm	Sum_cyclic_hc_norm	-0.5011	0.006
Sum_aromatic_hc_norm	Sum_terpenes_norm	0.4993	0.006
Sum_cyclic_hc_norm	ITEX Trap material_Carbosieve SIII	0.4963	0.006
Azines_norm	ITEX Trap material_Carboxen	-0.4958	0.006
Sum_cyclic_hc_norm	Sum_phtalates_norm	-0.4926	0.007
Extraction temperature	Sum_ketones_norm	0.4862	0.007
Sum_aldehydes_norm	Lactams_norm	0.4796	0.008
Sum_aliphatic_hc_norm	ITEX Trap material_Carboxen	-0.4779	0.009
Sum_cyclic_hc_norm	Sum_nitriles_norm	-0.4775	0.009
Sum_ketones_norm	Lactams_norm	0.4746	0.009
Sum_all_norm	Sulfones_norm	-0.4742	0.009
Sum_esters_norm	ITEX Trap material_Carbosieve SIII	-0.4739	0.009
Azines_norm	ITEX Trap material_Tenax TA	0.4709	0.010
Sum_alcohols_norm	Sum_cyclic_hc_norm	-0.4677	0.011
Sum_all_norm	Sum_cyclic_hc_norm	-0.4646	0.011
Sum_substituted_aromats_norm	Sulfones_norm	-0.4561	0.013
Sum_all_norm	ITEX Trap material_Tenax TA	0.4552	0.013
Sum_nitriles_norm	Sulfones_norm	-0.4458	0.015
Sulfones_norm	Sum_terpenes_norm	-0.4456	0.015
Sum_ketones_norm	Sulfones_norm	-0.4431	0.016
Sum_esters_norm	Sulfones_norm	-0.4412	0.017

Sum_cyclic_hc_norm	ITEX Trap material_Tenax TA	-0.4409	0.017
Sum_all_norm	Sum_aromatic_hc_norm	0.4365	0.018
Desorption flow	Lactams_norm	0.4315	0.019
Lactams_norm	ITEX Trap material_Carboxen	-0.4304	0.020
Sum_esters_norm	Sum_hetero_aromats_norm	-0.4253	0.021
Sum_terpenes_norm	ITEX Trap material_Tenax TA	0.4238	0.022
Sum_aldehydes_norm	Sum_esters_norm	0.4226	0.022
Sum_phtalates_norm	ITEX Trap material_Carboxen	0.4185	0.024
Sum_all_norm	ITEX Trap material_Carbosieve SIII	-0.4175	0.024
Desorption flow	Azines_norm	0.4138	0.026
Azines_norm	Sum_ketones_norm	0.4054	0.029
Sum_aldehydes_norm	ITEX Trap material_Tenax TA/Carboxen	0.4015	0.031
Sum_esters_norm	ITEX Trap material_Tenax TA/Carbosieve SIII	0.3994	0.032
Sum_cyclic_hc_norm	Sum_lactones_norm	-0.3982	0.032
Sum_lactones_norm	ITEX Trap material_Carboxen	0.3877	0.038
Sum_alcohols_norm	ITEX Trap material_Carbosieve SIII	-0.3869	0.038
Sum_ketones_norm	ITEX Trap material_Tenax TA/Carboxen	0.3819	0.041
Sum_aldehydes_norm	ITEX Trap material_Tenax TA	0.3814	0.041
Sum_aliphatic_hc_norm	Sulfones_norm	-0.3797	0.042
Sum_aromatic_hc_norm	Sum_ketones_norm	0.3787	0.043
Sum_aliphatic_hc_norm	Sum_cyclic_hc_norm	-0.3773	0.044
Sum_aldehydes_norm	Sum_phtalates_norm	0.3738	0.046
Azines_norm	Sum_hetero_aromats_norm	-0.3710	0.048
Sum_ketones_norm	ITEX Trap material_Carbosieve SIII	-0.3695	0.049
Sulfones_norm	ITEX Trap material_Carboxen	0.3691	0.049
Sum_alcohols_norm	ITEX Trap material_Carboxen	-0.3674	0.050
